# Genome-Wide Analysis Using Exon Arrays Demonstrates an Important Role for Expression of Extra-Cellular Matrix, Fibrotic Control and Tissue Remodelling Genes in Dupuytren's Disease

**DOI:** 10.1371/journal.pone.0059056

**Published:** 2013-03-12

**Authors:** Helen B. Forrester, Peter Temple-Smith, Seungmin Ham, David de Kretser, Graeme Southwick, Carl N. Sprung

**Affiliations:** 1 Centre for Innate Immunology and Infectious Disease, Monash Institute of Medical Research, Monash University, Clayton, Victoria, Australia; 2 Department of Obstetrics and Gynaecology, Southern Clinical School, Monash University, Monash Medical Centre, Clayton, Victoria, Australia; 3 Prince Henry's Institute, Clayton, Victoria, Australia; 4 Centre for Reproduction and Development, Monash Institute of Medical Research, Clayton, Victoria, Australia; 5 Department of Anatomy and Developmental Biology, Monash University, Clayton, Victoria, Australia; 6 Melbourne Institute of Plastic Surgery, Malvern, Victoria, Australia; University Hospital S. Maria della Misericordia, Udine, Italy

## Abstract

Dupuytren's disease (DD) is a classic example of pathological fibrosis which results in a debilitating disorder affecting a large sector of the human population. It is characterized by excessive local proliferation of fibroblasts and over-production of collagen and other components of extracellular matrix (ECM) in the palmar fascia. The fibrosis progressively results in contracture of elements between the palmar fascia and skin causing flexion deformity or clawing of the fingers and a severe reduction in hand function. While much is known about the pathogenesis and surgical treatment of DD, little is known about the factors that cause its onset and progression, despite many years of research. Gene expression patterns in DD patients now offers the potential to identify genes that direct the pathogenesis of DD. In this study we used primary cultures of fibroblasts derived from excisional biopsies of fibrotic tissue from DD patients to compare the gene expression profiles on a genome-wide basis with normal control fibroblasts. Our investigations have identified genes that may be involved with DD pathogenesis including some which are directly relevant to fibrosis. In particular, these include significantly reduced expression levels of three matrix metallopeptidases (*MMP1*, *MMP3*, *MMP16*), follistatin, and *STAT1*, and significantly increased expression levels of fibroblast growth factors (*FGF9*, *FGF11*), a number of collagen genes and other ECM genes in DD patient samples. Many of these gene products are known to be involved in fibrosis, tumour formation and in the normal processes of tissue remodelling. In addition, alternative splicing was identified in some DD associated genes. These highly sensitive genomic investigations provide new insight into the molecular mechanisms that may underpin the development and progression of DD.

## Introduction

Dupuytren's disease (DD) is a classic example of a pathological fibrotic disease characterized by excessive proliferation of fibroblasts and over-production of collagen and other components of extracellular matrix (ECM) in the hand [1], [2], [3]. DD typically starts in individuals between the age of 35 and 50 with the formation of one or more subcutaneous nodules in the palmar fascia that develop cords of fibrotic tissue impacting on the function of the metacarpophalangeal and proximal interphalangeal joints [1], [2], [3]. With time the developing fibrosis causes contracture of the palmar fascia resulting in flexion deformity of particularly the 4^th^ and 5^th^ digits with a severe reduction in hand function [4], [5], [6], [7], [8]. DD occurs in all racial groups but has a higher prevalence in populations with Caucasian ancestry [9], [10], [11]. Evidence from the pattern of inheritance observed in different populations suggests that it is heritable as an autosomal dominant or autosomal recessive with variable penetrance [12], [13], [14].

Males are three times more likely to develop DD and are also more likely to have greater disease severity [15], [16]. DD affects more than 2% of individuals in a population with the incidence in some cohorts, for example, Belgian and German men over 50 and men over 60 in Australia and Scotland, exceeding 20% (http:www.dupuytren-online.info/dupuytren_age_distribution.html) [12], [13], [17], [18]. The predominance of DD in males may be related to expression of androgen receptors in Dupuytren fascia [16]. Other risk factors include manual labor with vibration exposure, prior hand trauma, alcoholism, smoking, diabetes mellitus, hyperlipidemia, Peyronie's disease of the penis, and complex regional pain syndrome [19].

Currently, the most effective treatment is palmar fasciectomy, which involves surgical resection of fibrotic bands and rearrangement of skin using flaps or interrupting normal skin topography by full thickness grafts. Unfortunately, with time, the recurrence rate is high and disease progression is inevitable, requiring further surgery. Other surgical options include simple transcutaneous division of a Dupuytren's band by needle or knife fasciotomy [20], [21], with a high recurrence rate of about 3% in the first year and more than 50% by year 4. More recently, a nonsurgical treatment using subcutaneous injections of Clostridium-derived collagenase has been used [22], [23], [24].

While the descriptive pathogenesis of DD is understood [1], [16], the factors causing its onset and progression remain unclear [16], [25]. Fibrogenic cytokines that cause growth and differentiation of fibroblasts and production of ECM, such as epidermal growth factor (EGF), transforming growth factor-alpha and –beta (TGF-α, TGF-β) and platelet-derived growth factor (PDGF), have been implicated in DD [26], [27]. Gene expression has also been investigated in various tissues or primary cells from DD patients using various experimental approaches and designs, and different gene expression platforms and methods of analysis [28]. Most have identified candidate genes, but many of the older gene expression studies did not have the sensitivity or depth of coverage [29]. Recent use of higher density arrays has confirmed and extended some gene expression profiles in DD patient tissues and identified additional candidate genes [28], [30], [31], [32]. These include genes associated with tissue remodelling such as matrix metalloproteinases [28], [29], [31], [33], [34], those which have roles in the ECM, including fibronectin (*FB1*) laminins (*LAMB1*), integrins and thrombospondin (*THDS2*) [32] and others in which the link between the altered expression at both the transcriptional and translational levels like myoglobin and the tyrosine kinase orphan receptor 2 (*ROR2*) is less clear in terms of pathogenesis [30]. Some collagen genes show higher transcript levels in DD cord or nodal tissue [28], [29], [31], [32], [35] while other genes that have been implicated in DD include *ADAMT* (A disintergrin and metalloproteinase with thrombospondin motifs) genes [33], [34], [36], proteoglycans 4 (*PRG4*), A disintegrin and metalloproteinase domain 12 (*ADAM12*), fibulin-1 (*FBLN1*), and tenascin C (*TNC*) genes [36], [37].

DD development and progression has been linked to genes associated with various signalling pathways (e.g., the sonic hedgehog pathway) [28] and others involved in the TGFβ signalling pathway (*TGF*β*1, KLF6, SMADs*), known to be involved in proliferation, differentiation and fibrosis [32], [38], [39]. In contrast, known inhibitors of the TGFβ signalling pathway have been negatively associated with DD [40]. Genes in the AKT (*POSTN, RACK1, VCP*) and β-catenin and Wnt signalling pathways (*CTNNB1*, *WNT2, WNT4, RSPO2, SFRP1, SFRP4, ZIC1*) have also been implicated in DD [41], [42]. *MAFB*, a tissue development and cellular differentiation transcription factor, was increased in 50% of DD tissue but not in controls [35]. While most of these expression studies have used biopsied primary tissue, some investigations have narrowed their focus to a specific fibrogenic cell type involved, namely fibroblasts [36], [37].

Increased collagen expression has been found in DD patients [31], [35], [43] and recently collagenase has been used to treat DD [4], [25], [44] with initial studies showing that collagenase injections, though initially painful, are generally effective in reducing the effects of DD.

In previous studies, the use of tissue biopsies containing multiple cell types or short term culturing prior to processing raises the issue of heterogeneity in samples leading to increased noise and decreased assay sensitivity. In this study, we have use DD patient-derived cultured primary fibroblast cells to examine gene expression to identify new genes linked to the development of DD. The use of homogeneous populations of fibroblasts from DD patients provide an unique tissue source to identify genes linking to the development of DD as it removes the potential “noise” associated with the use of biopsies containing multiple cell types and effectively eliminates background and improves gene expression profiles. These data from genome-wide gene expression profiles were then compared to those from a complementary series of control primary fibroblast cells. Use of these control samples eliminated any DD genetic effect that may occur when adjacent tissue is used as a control. The novel use of exon arrays has identified new candidate genes associated with DD that provide further insights into this complex debilitating disorder.

## Materials and Methods

### Ethics statement

The study was approved by the Cabrini Human Research Ethics Committee (Approval #08-29-01-08) and confirmed by the Monash University Human Monash University Ethics Committee.

### Primary cells

Primary fibroblasts were obtained from cord biopsies taken from five DD patients with extensive fibrosis of the palmar fascia during radical palmar fasciectomy. Of the five DD patients, four were male aged 37 to 75 years old and were stage 2 or 4, and one was female aged 54 and stage 1. Control primary fibroblast cells were derived from thigh skin punch biopsies from 6 individual females unaffected by DD [45]. Biopsies from the DD patients were minced finely, plated out into T25 Falcon flasks (BD Biosciences) containing 4–5 ml of DMEM/F12 medium (Invitrogen) with 5% serum and grown to confluence at 37°C in 4.5–5% CO_2_. Fibroblasts from these cultures were harvested using standard procedures, washed in medium, and replated in 12 ml of medium at a concentration of 5×10^5^ cells/ml in T75 Falcon flasks. Culture medium in each flask was changed once or twice weekly and cultures were grown to ∼80% confluency before harvesting. For most patient samples, the cell cultures had undergone less than five passages since initiation, but had been grown to confluence in culture for several months. Most control cell samples had been grown in culture for less than ten passages. All patients gave written informed consent for their tissues to be used in this study.

### RNA Isolation

Ten million fibroblasts from each DD and control cell line were pelleted, resuspended in 3 ml PBS with an equal volume of Trizol (Invitrogen, Carlsbad, CA, USA), mixed and incubated at room temperature for 15 minutes. Chloroform was then added and the mixture was centrifuged to separate the aqueous from the organic layer. The aqueous layer was mixed with an equal volume of 70 percent ethanol and loaded on to an RNeasy column (Qiagen, Venlo, The Netherlands). The RNA extraction was continued using the RNeasy method as per the manufacture's recommendation except that the procedure was started at the step where Buffer RW1 is added. RNA concentration and integrity was determined using a bioanalyzer (Agilent, Santa Clara, CA, USA). RNA was determined to be high enough quality for exon array analysis if a minimum RIN of 8.5 was obtained.

### Exon arrays

GeneChip Human Exon 1.0 ST Array analysis was performed on samples from 4 DD patients and 6 control patient (10 arrays) as per the GeneChip Whole Transcript (WT) Sense Target labelling assay instructions (Affymetrix, Santa Clara, CA, USA). The rRNA from 1 µg of total RNA was reduced using a RiboMinus Human/Mouse Transcriptome Isolation Kit (Invitrogen, Carlsbad, CA, USA). For this investigation we analysed the ‘core set’ that is defined by over 228,000 probe set regions (Affymetrix.com). Array quality was assessed using Expression Console (Affymetrix.com). For differential gene expression, all exon arrays were normalized with robust multi-array average (RMA) background correction and quantile normalization, and overall transcript expression was estimated using an exon RMA linear model [46]. Gene expression levels were determined using AS ANOVA or ANOVA algorithms provided in the Partek Genomics Suite statistical analysis package (Partek, St Louis, MO, USA). Array data and normalized expression values have been deposited in the gene expression omnibus database: accession number GSE41524.

### Transcriptional validation

All primer sequences for candidate exons or genes (see Table S1) were designed using NCBI primer blast (ncbi.nih). cDNA was prepared from total RNA using Superscript III as per manufacturer's recommendation (Invitrogen, San Diego, USA). Normal PCR amplification for each sample was carried out using GoTaq Green Master Mix containing GoTaq DNA polymerase (Promega, Wisconsin, USA), 200 nM primers, 8 ng cDNA with a cycling protocol of 94°C: 3 min; ((94°C: 30 sec; 60°C: 30 sec; 72°C: 30 sec)×35); 72°C: 7 min. Products were run on a 2% agarose gel to determine amplification of the proper sized product. Real-time PCR was performed using these primers under the following conditions: Power SYBR Green Master Mix (Applied Biosystems, United Kingdom) was mixed with 5 to 10 ng of cDNA (per sample) and 2 pmol of each primer; the cycling steps used in the PCR were 95°C: 10 min; ((95°C: 15 sec; 60°C: 60 sec)×40); with a melting curve temperature ramp following.

### Alpha smooth muscle actin staining

DD and control primary fibroblasts were plated on glass cover slips in 6 well plates. Attached cells were washed twice with PBS and fixed with cold methanol for 20 mins at 4°C and washed twice with PBS. Cells were incubated with Image-iT ™ FX Signal Enhancer (Invitrogen #I36933) for 1 hour at room temperature and then washed twice with PBS. Cell preparations were incubated overnight at 4°C with anti-αSMA antibody (abcam #ab5694) diluted with 5% BSA in PBS (1∶100), washed twice with PBS and incubated for 1 hour at room temperature with Alexa Fluor 488 goat anti-rabbit (1∶1000; Invitrogen #A11006) and Hoechst 33342 (1∶2000) diluted in 5% BSA in PBS. Cells were washed twice with PBS and mounted with Fluorsave (Calbiochem #345789). Images were taken with a Nikon C1 inverted microscope.

## Results

### Genome-wide exon expression profiles

Whole genome Affymetrix exon arrays were used to determine gene expression profiles in DD patient primary fibroblasts. For these studies, the filtered ‘core’ set of probe selection regions (PSRs; RefSeq transcripts and full length mRNAs; Affymetrix.com), which include well-documented exon regions, were used for analysis.

**Figure 1:**
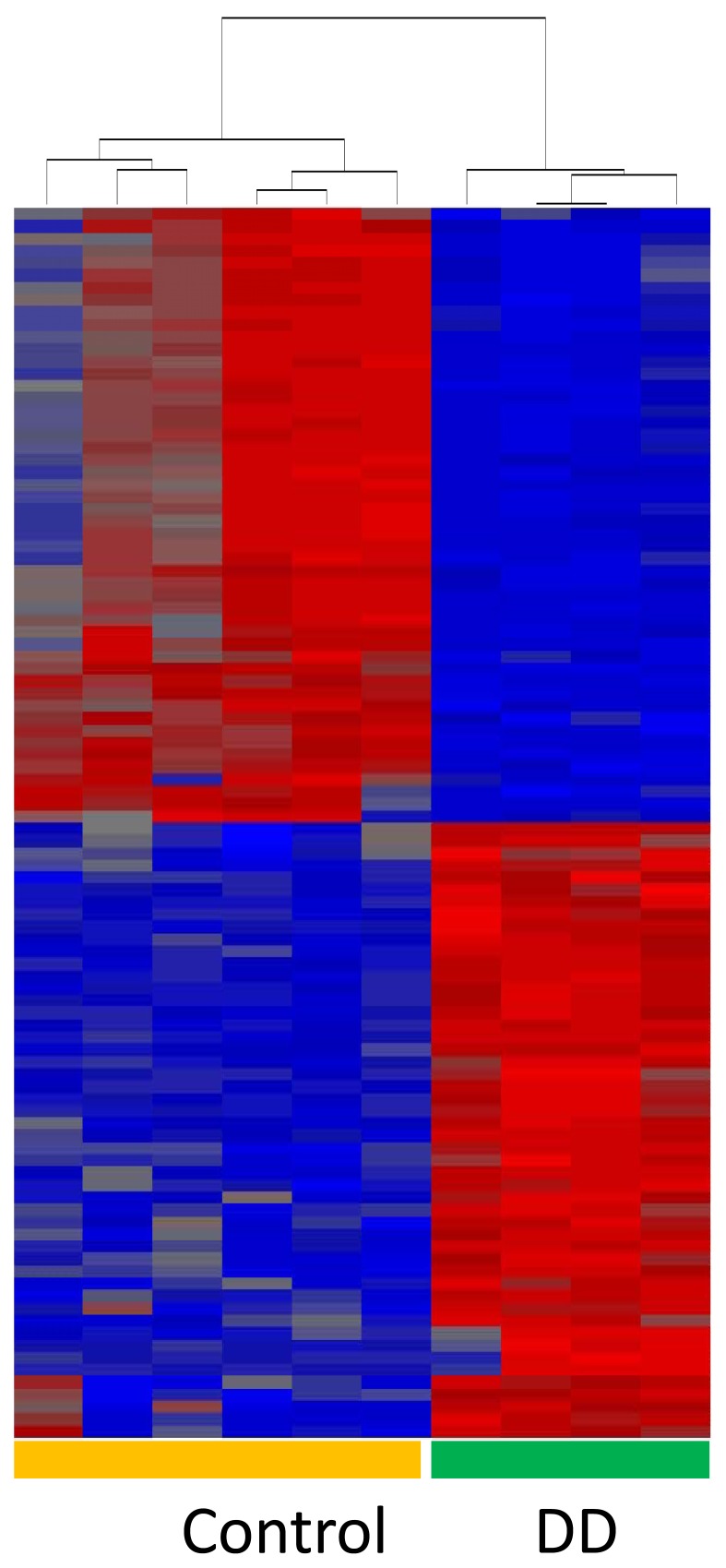
Dupuytren's disease (DD) samples show many genes that are differentially expressed compared to control samples. Cluster analysis and heat map of the top 50 genes showing highest or lowest gene expression in DD patient fibroblasts compared to controls. Genes are represented on individual rows while columns represent control or DD patient samples. Transcript levels that are relatively higher (red) or lower (blue) are color coded.

Comparison of expression profiles from DD patient-derived primary fibroblast cells with those of the six control primary fibroblast cells from patients with no history of DD identified 307 genes with significantly higher, and 1288 genes with significantly lower, transcript levels (ANOVA p-values <0.05 after multiple test correction) and fold changes exceeding 2.0 in DD (Tables 1, 2: top 50 and Tables S1, S2: full list). Cluster analysis and heat maps for individual samples for the top genes (p-value <0.05 after multiple test correction and top 50 increased and decreased genes) showed a clear difference in Dupuytren's disease fibroblast gene expression when compared to normal fibroblasts (Figure 1). Many of these expression differences were associated with ECM genes (e.g., *COMP*, collagen) but other genes showing differences encoded for proteins that influence fibrosis (e.g., follistatin), tissue remodelling (e.g., collagenases and matrix metallopeptidase proteins) and signalling pathways (e.g., *STAT1, WNT2* and *WNT4*), growth factors (e.g., *FGF9*) and cell movement (e.g., *KIF* genes). Genes that showed differential expression and were located on the Y chromosome were eliminated from analysis. Furthermore, validated genes with differential expression were found to have no gender bias.

**Table 1:** Top 50 genes that show higher gene expression in DD samples based on largest expression level difference from controls.

Gene Symbol	Gene ID (RefSeq)	p-value	Fold-Change(Control vs. DD)	Fold change direction
VCAM1	NM_001078	5.07E-11	−35.1342	CL down vs DD
COMP	NM_000095	8.61E-21	−34.3401	CL down vs DD
SFRP4	NM_003014	1.10E-02	−30.7416	CL down vs DD
CHI3L1	NM_001276	2.86E-16	−20.9531	CL down vs DD
DDIT4	NM_019058	7.80E-10	−17.4606	CL down vs DD
SCRG1	NM_007281	3.50E-19	−14.576	CL down vs DD
C10orf10	NM_007021	4.36E-04	−13.2329	CL down vs DD
FGF9	NM_002010	4.57E-03	−11.8243	CL down vs DD
FMOD	NM_002023	1.91E-03	−9.95692	CL down vs DD
KRT34	NM_021013	1.53E-06	−9.59421	CL down vs DD
CHAC1	NM_024111	7.00E-03	−9.54938	CL down vs DD
CADM1	NM_014333	2.71E-10	−9.35959	CL down vs DD
THBS4	NM_003248	1.40E-30	−8.74083	CL down vs DD
PTPRD	NM_002839	5.86E-29	−8.59582	CL down vs DD
CRLF1	NM_004750	1.98E-27	−8.46392	CL down vs DD
SPON1	NM_006108	4.48E-09	−7.64994	CL down vs DD
PAPPA2	NM_020318	1.11E-36	−7.16121	CL down vs DD
ANGPTL4	NM_139314	2.96E-11	−6.89301	CL down vs DD
PFKFB4	NM_004567	6.37E-23	−6.27251	CL down vs DD
DACT1	NM_016651	1.06E-15	−6.24109	CL down vs DD
C7orf68	NM_013332	9.42E-04	−5.78641	CL down vs DD
NRCAM	NM_001193582	6.68E-23	−5.72873	CL down vs DD
SLC2A5	NM_003039	1.97E-25	−5.64975	CL down vs DD
NDUFA4L2	NM_020142	2.48E-03	−5.64615	CL down vs DD
IL26	NM_018402	1.15E-04	−5.58784	CL down vs DD
CILP2	NM_153221	5.12E-24	−5.41481	CL down vs DD
ANKRD37	NM_181726	0.000196107	−5.39884	CL down vs DD
DAPK1	NM_004938	3.24E-09	−5.29651	CL down vs DD
CPA4	NM_016352	3.85E-02	−5.18093	CL down vs DD
RBP4	NM_006744	2.47E-04	−5.17563	CL down vs DD
LOXL3	NM_032603	2.70E-03	−5.08977	CL down vs DD
MOCOS	NM_017947	4.29E-08	−5.00414	CL down vs DD
ASPHD2	NM_020437	1.16E-06	−4.91587	CL down vs DD
UNC5B	NM_170744	1.68E-08	−4.90931	CL down vs DD
WNT2	NM_003391	1.39E-05	−4.82891	CL down vs DD
SPAG4	NM_003116	7.90E-23	−4.60329	CL down vs DD
RDH10	NM_172037	1.31E-02	−4.58245	CL down vs DD
TRIB3	NM_021158	3.67E-09	−4.46002	CL down vs DD
APLN	NM_017413	2.06E-08	−4.34215	CL down vs DD
MSC	NM_005098	5.40E-04	−4.33854	CL down vs DD
MTHFD2	NR_027405	2.00E-03	−4.3355	CL down vs DD
CPZ	NM_001014448	6.94E-11	−4.28206	CL down vs DD
COL15A1	NM_001855	1.18E-03	−4.26303	CL down vs DD
SLC7A5	NM_003486	2.08E-06	−4.25905	CL down vs DD
PDLIM3	NM_014476	4.79E-05	−4.22887	CL down vs DD
TFAP2B	NM_003221	3.30E-09	−4.17167	CL down vs DD
CXCL16	NM_022059	1.19E-07	−4.15755	CL down vs DD
PRG4	NM_005807	1.76E-10	−4.15362	CL down vs DD
SLC2A1	NM_006516	4.14E-09	−4.13188	CL down vs DD
CBS	NM_000071	1.10E-07	−4.1273	CL down vs DD

DD: Dupuytren's disease; CL: control.

**Table 2:** Top 50 genes that show lower gene expression in DD samples based on largest expression level difference from controls.

Gene Symbol	Gene ID (RefSeq)	p-value	Fold-Change(Control vs. DD)	Fold change direction
MMP1	NM_002421	8.63E-14	56.6277	CL up vs DD
SEMA3A	NM_006080	1.63E-24	32.9807	CL up vs DD
KIT	NM_000222	0	19.8159	CL up vs DD
MMP3	NM_002422	2.75E-14	18.5218	CL up vs DD
PBK	NM_018492	1.02E-04	14.2813	CL up vs DD
TFPI	NM_006287	1.47E-18	14.109	CL up vs DD
SERPINB2	NM_001143818	1.32E-08	14.0724	CL up vs DD
TFPI2	NM_006528	9.40E-04	13.4744	CL up vs DD
KIF11	NM_004523	2.38E-05	13.0349	CL up vs DD
TOP2A	NM_001067	4.95E-36	12.6288	CL up vs DD
CTSK	NM_000396	1.26E-07	12.4257	CL up vs DD
TRPC4	NM_016179	6.46E-05	11.8229	CL up vs DD
IL13RA2	NM_000640	1.40E-04	11.7517	CL up vs DD
ANLN	NM_018685	2.06E-23	11.0621	CL up vs DD
DLGAP5	NM_001146015	3.47E-23	10.8337	CL up vs DD
LPHN2	NM_012302	2.54E-38	10.8311	CL up vs DD
CENPF	NM_016343	1.35E-22	9.51298	CL up vs DD
EEA1	NM_003566	3.44E-16	9.30826	CL up vs DD
KIF20B	NM_016195	9.28E-30	9.06	CL up vs DD
SMC2	NM_001042550	4.04E-18	8.26822	CL up vs DD
HMCN1	NM_031935	0.00E+00	8.08127	CL up vs DD
ROCK1	NM_005406	4.43E-20	8.07519	CL up vs DD
NCAPG	NM_022346	9.35E-26	7.93099	CL up vs DD
KIF23	NM_138555	3.38E-27	7.87122	CL up vs DD
GALNT5	NM_014568	4.63E-02	7.74798	CL up vs DD
MKI67	NM_002417	7.71E-16	7.68071	CL up vs DD
CASC5	NM_170589	0.00E+00	7.52791	CL up vs DD
CKAP2	NM_018204	1.64E-30	7.5254	CL up vs DD
ASPM	NM_018136	0.00E+00	7.37628	CL up vs DD
KIF18A	NM_031217	9.13E-06	7.28334	CL up vs DD
SCIN	NM_001112706	2.34E-11	7.28039	CL up vs DD
MELK	NM_014791	2.78E-22	7.11972	CL up vs DD
VPS13C	NM_020821	0.00E+00	7.1057	CL up vs DD
TTK	NM_003318	2.62E-16	7.00696	CL up vs DD
CENPK	NM_022145	1.49E-08	6.94789	CL up vs DD
HELLS	NM_018063	2.60E-27	6.94526	CL up vs DD
SHCBP1	NM_024745	2.66E-21	6.74075	CL up vs DD
CEP55	NM_018131	5.33E-15	6.70633	CL up vs DD
KITLG	NM_000899	3.62E-03	6.60831	CL up vs DD
RECQL	NM_002907	1.09E-21	6.56328	CL up vs DD
PODXL	NM_001018111	1.32E-20	6.47796	CL up vs DD
HMMR	NM_001142556	9.34E-16	6.46258	CL up vs DD
CD109	NM_133493	2.08E-02	6.42551	CL up vs DD
ECT2	NM_018098	2.02E-16	6.42206	CL up vs DD
NUF2	NM_145697	3.60E-21	6.40957	CL up vs DD
KIF5B	NM_004521	2.43E-27	6.31599	CL up vs DD
ITGA2	NM_002203	1.59E-16	6.27779	CL up vs DD
GAS2L3	NM_174942	1.09E-09	6.27478	CL up vs DD
GGH	NM_003878	5.16E-04	6.25643	CL up vs DD
DST	NM_015548	0.00E+00	6.25435	CL up vs DD
FAM111B	NM_198947	3.78E-06	6.22711	CL up vs DD

DD: Dupuytren's disease; CL: control.

### Metalloproteinase and Collagen Genes

**Figure 2:**
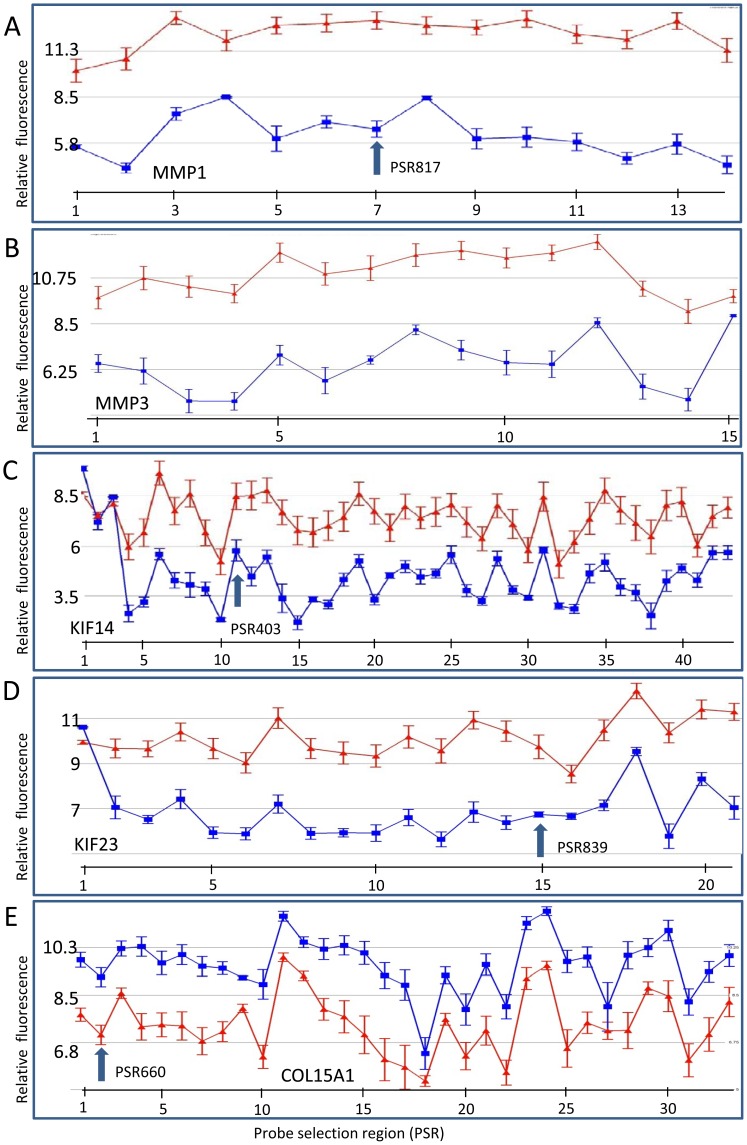
The gene expression for several genes associated with collagen metabolism are modulated differently in DD patient fibroblasts compared to control fibroblasts. (A) *MMP1*, (B) *MMP3*, (C) *KIF14*, (D) *KIF23* and (E) *COL15A1*. Relative PSR fluorescence (y-axis) is plotted for each PSR (points along x-axis). Samples were either from DD fibroblast (blue) or skin fibroblasts from patients without DD (red). PSRs are oriented 5′ to 3′ across the gene from left to right on the x-axis. Relative expression levels are plotted on a log_2_ scale. Arrow represents a PSR region that was used for subsequent PCR validation. Four separate samples from different individuals were used for the DD cohort and six different individuals' samples for control fibroblasts. Error bars  =  SEM. Analysis was performed as described in the Materials and Methods section. Actual PSR numbers are shown in Figure S1.

**Figure 3:**
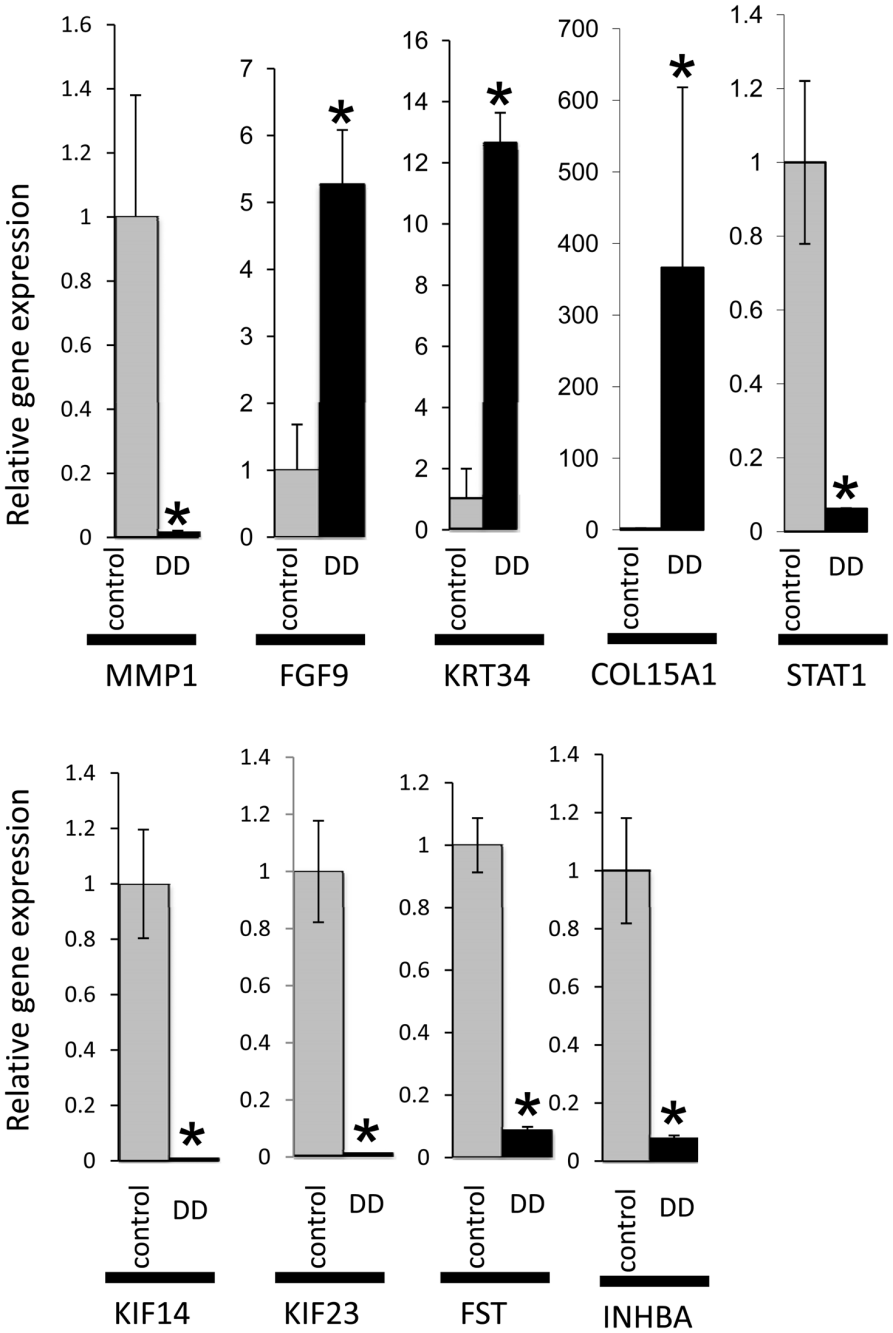
qRT-PCR validation of gene expression in fibroblasts from DD and control cells. The relative fold change in *MMP1*, *FGF9*, *KRT34*, *COL15A1*, *STAT1*, *KIF14*, *KIF23*, *FST* and *INHBA* gene expression in DD fibroblasts compared to control fibroblasts. The p-values using an unpaired t-test are: 0.019, 0.001, 0.03, 0.012, 0.03, 0.02, 0.00003, and 0.0058 for *MMP1*, *FGF9*, *KRT34*, STAT1, *KIF14*, *KIF23*, *FST* and *INHBA* respectively (n = 5 for both control and DD, except for *STAT1* where n = 3 for DD). The p-value using Mann-Whitney U test is 0.008 for *COL15A1*. Error bars represent the SEM.

**Figure 4:**
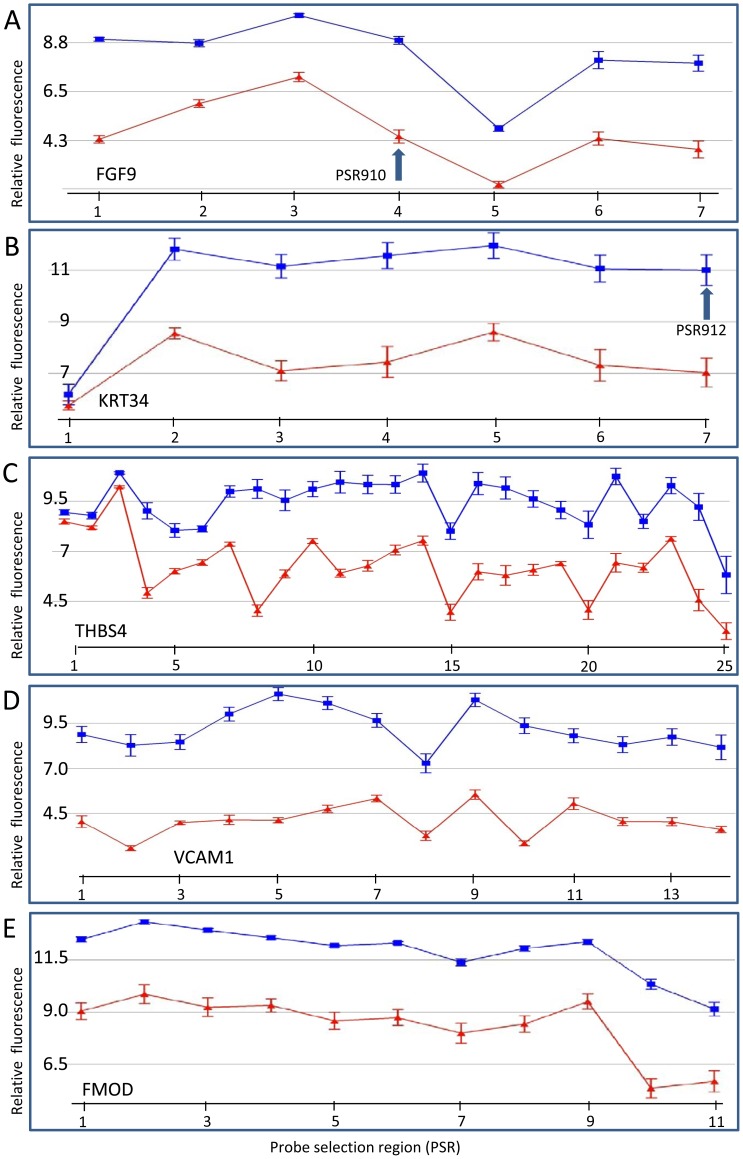
The gene expression of several genes that are modulated differently in DD patient fibroblasts compared to control fibroblasts. (A) *FGF9*, (B) *KRT34*, (C) *THBS4*, (D) *VCAM1* and (E) *FMOD*. Relative PSR fluorescence (y-axis) is plotted for each PSR (points along x-axis). Samples were either from DD fibroblast (blue) or skin fibroblasts from patients without DD (red). PSRs are oriented 5′ to 3′ across the gene from left to right on the x-axis. Relative expression levels are plotted on a log_2_ scale. Arrow represents a PSR region that was used for subsequent PCR validation. Four separate samples from different individuals were used for the DD cohort and six different individuals' samples for control fibroblasts. Error bars  =  SEM. Actual PSR numbers are shown in Figure S1.

The top gene identified in these gene expression studies was matrix metallopeptidase protein 1 (*MMP1*). This interstitial collagenase had a 56 fold reduction in expression levels in DD fibroblasts (Figure 2, Table 2). qRT-PCR using five DD and five control samples confirmed that *MMP1* expression was significantly lower in DD samples (p<0.05; Figure 3). Microarray gene expression analysis of other MMPs, (e.g. *MMP3*: Table 2, and *MMP16*: Table S2) also showed lower expression levels in the DD group. Conversely, 24 collagen genes, including *COL15A1* (Figure 2 and 3, Table 1), *COL5A1, COL5A2*, *COL4A1, COL4A2, COL4A4* (Table S1), *COL1A1*, and *COL3A1* (Figure S1) showed significantly higher level of expression in the DD patient-derived fibroblasts. There was also an increase in expression of fibromodulin (*FMOD*) (Figure 4 and Table 1), a gene product that interacts with collagen fibrils and is involved in the formation of the ECM, the collagen chaperone, *SERPINH1* (Table S2), and *LOXL3*, a catalyst for crosslinks in collagen and elastin (Table 1). RT-PCR analysis showed that the *COL15A1* levels varied greatly between the individual DD patients (between 12.5 and 1450) and this was reflected in the large SEM (Figure 3). However, there was a clear difference between the two groups with the lowest DD *COL15A1* expression levels being 3.2 fold higher than the highest control patient expression levels and a p-value of 0.008 (Mann-Whitney U-test). This variation was not associated with differences in age, stage or gender of the DD patients. Other metalloproteinases, *ADAM15, ADAMST10, ADAMST2* and *ADAMTS3,* had increased transcription levels (approximately 2.3 to 2.5 fold) in DD fibroblasts (Figure S1). However, these genes are also involved in increasing cell adhesion and decreasing cell mobility (see “Cell adhesion, cell-to-cell, and cell-to-matrix interaction genes” below).

### Follistatin and TGFβ Super Family Genes

**Figure 5:**
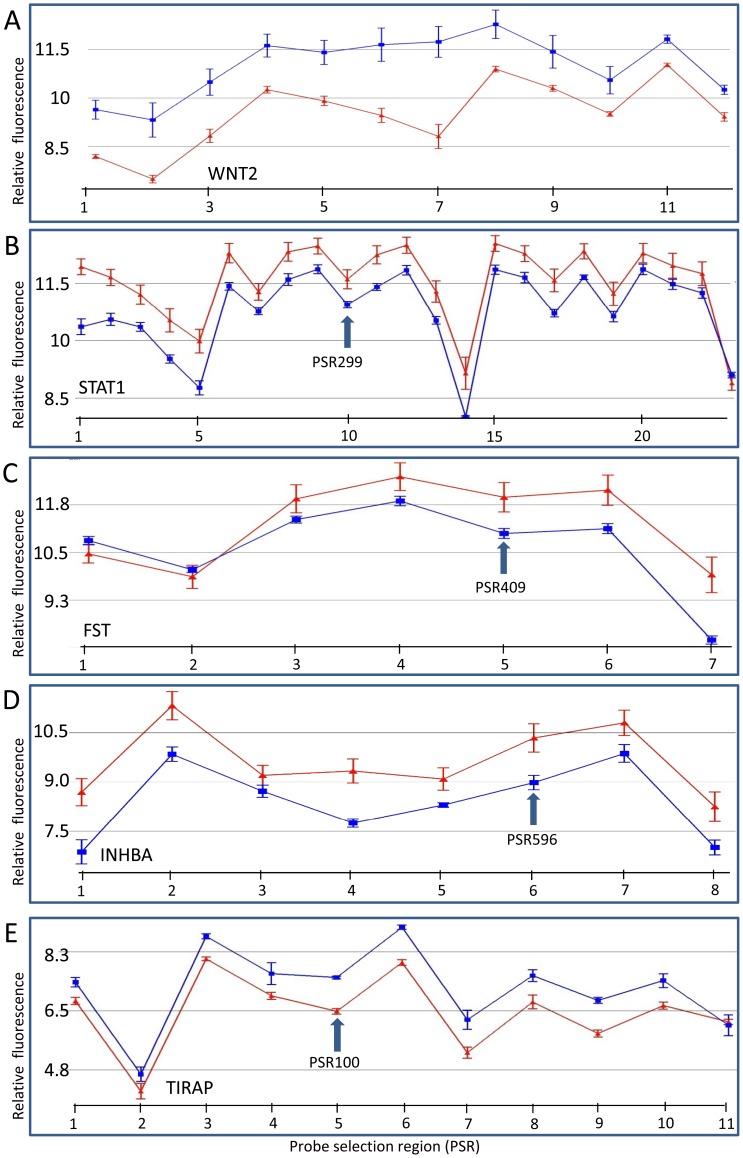
The gene expression of genes that are modulated differently in DD patient fibroblasts compared to control fibroblasts including those involved in the inflammatory response and tissue remodelling. (A) *WNT2*, (B) *STAT1*, (C) *FST*, (D) *INHBA* and (E) *TIRAP*. Relative PSR fluorescence (y-axis) is plotted for each PSR (points along x-axis). Samples were either from DD fibroblast (blue) or skin fibroblasts from patients without DD (red). PSRs are oriented 5′ to 3′ across the gene from left to right on the x-axis. Relative expression levels are plotted on a log_2_ scale. Arrow represents a PSR region that was used for subsequent PCR validation. Four separate samples from different individuals were used for the DD cohort and six different individuals' samples for control fibroblasts. Error bars  =  SEM. Actual PSR numbers are shown in Figure S1.

Array analysis showed that follistatin (*FST*) mRNA levels were significantly lower in DD fibroblasts (Figure 5) compared to controls. These results were verified using qRT-PCR (Figure 3) which showed that DD fibroblasts had a relative follistatin gene expression that was ∼10% of controls. The gene expression of *INHBA,* which codes for the β_A_ subunit component of activin and inhibin proteins, also showed lower levels in the DD fibroblasts compared to controls (Figures 3 and 5). However, qRT-PCR results indicated that the expression levels of *INHBB,* which codes for the β_B_ subunit of activin and inhibin proteins, varied greatly between individuals in both DD and control patient cells, so the difference between the groups was not significant (data not shown). *BMP4, a*nother member of the transforming growth factor family which codes for bone morphogenetic protein 4 had increased levels in DD (Figure S1).

### Other ECM and tissue remodelling genes

Transcripts from other genes involved in ECM and tissue remodelling also had different gene expression levels in DD compared to control fibroblasts. For example, cartilage oligomeric matrix protein (*COMP*), spondin 1 ECM protein (*SPON1*), cartilage intermediate layer proteins (*CLIP* and *CLIP2*), sarcoglycan (*SGCG*), elastin (*ELN*) and ficolin collagen/fibrinogen domain containing lectin 2 (hucolin: *FCN2*), and tumour necrosis factor, alpha-induced protein 6 (*TNFAIP6*), all had significantly higher levels of gene transcripts in DD compared to control fibroblasts (Figure S1). Angiopoietin-like 4 (*ANGPTL4*), a glycosylated, secreted protein that has a fibrinogen C-terminal domain and has many functions including inhibition of proliferation, migration and tubule formation in endothelial cells had higher levels of gene transcripts in DD (Figure S1). In addition, several genes involved in the re-absorption or re-modelling of ECM were significantly lower in DD. For example, the level of gene expression for cathepsin K (*CTSK*), a lysosomal cysteine proteinase involved in bone remodelling and resorption and possibly involved in ECM re-absorption, was significantly lower in DD samples. Plasmin-mediated matrix remodelling protein, tissue factor pathway inhibitor 2 (*TFPI2*), and *TFPI* transcripts were expressed at significantly lower levels in DD (Figure S1). There are also examples of ECM gene transcripts that were significantly lower in DD compared to control fibroblasts including laminin alpha 4 (*LAMA4*), fibronectin type III domain containing 3A (*FNDC3A*), and fibronectin leucine rich transmembrane protein (*FLRT2*) (Figure S1).

### Cell adhesion, cell-to-cell, and cell-to-matrix interaction genes

Genes coding for protein products that are involved in cell adhesion, cell-to-cell, and cell-to-matrix interactions had higher gene expression in DD fibroblasts than in controls (Figure 4, Table 1). These included vascular cell adhesion gene (*VCAM1*), which showed a 30-fold increase (Figure 4), cell adhesion molecule 1 (*CADM1*), chitinase 3 like 1 (*CHI3L1*), thrombospondin 4 (*THBS4)* (Figure 4), and neuronal cell adhesion molecule (NRCAM). In contrast, transcription levels of podocalyxin-like (*PODXL*) gene, a negative regulator of cell adhesion, was significantly lower in DD samples (Figure S1). Transcripts of several membrane glycoproteins involved in cell adhesion, integrin (ITG), alpha 11 gene (*ITGA11*), were significantly higher in DD (up 4.5 fold). However, the expression levels of some other integrin genes, for example, *ITGA2*, *ITGA6*, and *ITGA4,* were reduced 7.6–, 5.8- and 5.4-fold, respectively, in DD fibroblasts (Figure S1). Several metalloproteinases, *ADAM15, ADAMST10, ADAMST2* and *ADAMTS3* had increased transcription levels in DD fibroblasts (Figure S1).

### Rho-associated genes

Rho-kinases are involved in cytoskeletal rearrangement and cell motility. Several Rho-associated genes showed lower levels of transcripts in DD compared to controls. These included Rho-associated, coiled-coil containing protein kinase 1 (*ROCK1*) which stabilises actin in cells, Rho GTPase activating protein 11A (*ARHGAP11A*), sema domain immunoglobulin domain (Ig) short basic domain, secreted (semaphorin) 3A (*SEMA3A:* found in human tumour cell lines) and DEP domain containing 1 (*DEPDC1*), a transcriptional corepressor (Figure S1).

### Microtubule-based movement (KIFs) genes

Six genes in the kinesin family (*KIF11*, *KIF13A*, *KIF14*, *KIF18A*, *KIF20B*, and *KIF23*) that encode proteins involved in microtubule-based cellular movement showed significantly lower levels of expression in the DD patient fibroblasts (Figure S1). Reduced expression of several KIF genes detailed in the microarray profiles (Figure 2) have also been confirmed using qRT-PCR (Figure 3).

### Fibroblast growth factor genes

Higher expression levels (12-fold) of the fibroblast growth factor 9 gene (*FGF9*; glia-activating factor) (Figure 4), which is involved in growth stimulation and tissue repair, was observed in DD samples compared with controls and confirmed by qRT-PCR (Figure 3). *FGF11* gene expression was also increased significantly in DD fibroblasts (Figure S1).

### *KRT34* and other KRT genes

Expression levels of KRT34, a keratin gene for which the protein product is a major structural component of hair and nails, were significantly higher in DD patient samples compared to controls (Figure 4) and these increased expression levels were confirmed using qRT-PCR (Figure 3). Other keratin family genes that also showed significantly higher expression levels in DD patient fibroblasts included *KRT7*, *KRT16*, *KRT18*, *KRT19*, *KRT33A*, *KRT33B*, and *KRT81* (Table 1, Table S2, Figure S1).

### Wnt signalling genes

*WNT2* had a higher level of gene expression in DD fibroblasts than in controls (Figure 5). Secreted frizzled-related protein 4 (*SFRP4*), a potential modulator (inhibitor) of Wnt signalling, was another gene that showed a significantly higher level of gene transcripts in DD, but a related gene in this pathway (*SFRP1*) showed decreased expression in DD patients. There was also a significant reduction in transcripts of the ribonucleotide reductase M2 (*RRM2*) and *RSP03* genes in DD patient fibroblasts compared with controls (Figure S1). *RSPO3* is a member of the thrombospondin type 1 repeat supergene family and an activator of the β-catenin signalling cascade and *RRM*2 is an inhibitor of the Wnt pathway. DNA-damage-inducible transcript 4 (*DDIT4*), an inhibitor of cell growth, also shows an increase in expression in DD fibroblasts.

### Cell cycle genes

A large number of cell cycle genes had significantly reduced levels of transcripts in DD fibroblasts (Table S1). These included genes associated with mitosis, such as cyclin-dependent kinase 1 (*CDK1* or *CDC2*), *DLGAP5* (M phase), *CENPK*, *CENPF* (G2 phase), *MK167*, *NCAPG*, *CEP55*, *ASPM*, *SMC2*, *TPX2*, *CCDC99*, *RRM2* (DNA replication), *NUF2*, *NDC80* (mitotic sister chromatid segregation), *GAS2L3* (cell cycle arrest), *TOPO2A*, epithelial cell transforming sequence 2 oncogene (*ECT2*; G2 and M phase), and TTK protein kinase (TTK) and cancer susceptibility candidate 5 (*CASC5*), both essential for spindle-assembly checkpoint signalling and for correct chromosome alignment. However, one cell cycle gene, *CCND2* (G1/S transition), was expressed at significantly higher levels in DD than in control fibroblasts (Figure S1).

### Innate immune response genes

Several genes involved in the innate immune response had lower levels of transcripts in DD fibroblasts. The transcription factor, STAT1, had a lower number of transcripts in the DD patient samples and this was confirmed by qRT-PCR (Figures 3 and 5).

### Alternative splicing

Exon arrays identified transcript variants and provided a comparison of the expression of these variants between control and DD fibroblasts. Genes likely to have alternative transcripts include *THBS4*, *KRT34*, *TIRAP*, *KIF23* and *KIF14* and others (Figures 2, 4, 5 and S1). Some exons in these variants showed very little difference in expression between the samples which was especially common at the beginning of the transcript. For example, in the first region (located in exon 1) of the *THBS4* gene, there was little difference between control and DD fibroblasts, whereas the remaining exons in the gene showed a very distinct and large expression difference (Figure 4). In DD patients the *THBS4* transcript that was increased would code for a protein missing approximately the first 92 amino acids (aa) from the N-terminus. In the *KRT34* gene, the first PSR remained unchanged (within exon 1) and there was an increase in the other PSRs in DD (Figure 4). This increased *KRT34* transcript codes for a protein missing approximately the first 43 aa of the N-terminal. The *TIRAP* transcript in DD patients increased after the first two PSRs which were located in the first two exons (Figure 5). However, only the 5′ untranslated region (5′UTR) of the gene was involved, so the resulting protein was predicted to be unchanged. The expression profiles for both *KIF14* and *KIF23* indicated that the regions of the first three and one PSR, respectively (exon 1 in both genes), were unchanged but all the following PSRs were decreased in DD (Figure 2). Similar examples for other genes are shown in Figure S1.

### Alpha smooth muscle actin analysis

Both control and DD fibroblasts were positive for anti-αSMA staining and there was no noticeable difference in the intensity between the samples (Figure S2). *ACTA2* gene expression was the same for both cell type and *ACTA1* gene expression was only slightly higher in the DD patient cells than in the controls.

## Discussion

This study examined gene transcription at the exon level on a genome wide scale in DD patient fibroblasts. Each exon array provided extensive whole genome transcript coverage and allowed robust data acquisition for gene expression analysis superior to gene expression platforms used in earlier publications [29], [32], [35], [37], [47]. Of the more than 15,000 genes tested, 302 had significantly higher transcript levels (>2 fold) in DD and 1276 had lower transcript levels (>−2 fold) in DD when compared with controls.

Most of the previous studies have compared expression levels in tissue biopsies between diseased regions and unaffected regions from the same patients [28], [29], [31], [32], [33], [35], [36], [47] and a few of these studies also included comparisons with tissue samples from carpal tunnel release patients [28], [31], [35], another soft tissue hand disease associated with diabetes [48]. Few previous studies have compared expression levels in primary fibroblasts derived from the affected and unaffected regions of DD patients [49] or from the two patient types [37], [43]. One compared the tissue between DD patients and hand trauma patients [30]. Variation in the expression levels found can be accounted for by the differences in the cells contained in the tissue samples that were compared. In the tissue biopsies, the cell population consists of other cell types as well as fibroblasts. In addition, fibroblasts present in the DD tissue samples are in the diseased environment and expression levels may be influenced by the extensive cellular matrix and cell density. Differences in gene expression levels of primary fibroblast cells from diseased and non-diseased areas have been shown to decrease after 4 to 6 passages [50]. These different comparisons provide information about different aspects of the disease. The primary fibroblast cells used as controls in our study were derived from skin punch biopsies taken from the thigh and may elicit some fibroblast specific expression differences due to cell derivation from skin of a different region [51], but these cells are derived from cancer patients with no history of Dupuytren's disease or other soft tissue diseases and had no major scarring after radiation therapy. Variation between our findings and others may reflect genetic susceptibility to DD in addition to the disease state.

The major collagen found in the palmar fascia tissue is type I but in Dupuytren's nodules it has been reported that there is an increase in collagen and in particular, a higher proportion of type III compared to type I [52]. However, Murrell et al., 1991 found that the fibroblast cells (passage 3) from DD tissue and carpal tunnel control tissue did not show any noticeable difference in their collagen production and suggested that the increase in collagen type III to type I ratio found in the tissue samples was due to inhibition of collagen type I production in the fibroblasts growing in higher density as found in the DD tissue [53]. They demonstrated that an increase in fibroblast density resulted in an increase in the ratio of collagen type III to type I due to a decrease in collagen type I production. The fibroblasts used in the present study were grown to approximately the same density to avoid issues in expression differences associated with cell density.

Compelling evidence shows that the collagen-associated transcripts are a key component of progression of DD [28], [31], [32], [35], [43], [47]. Satish et al (2008) [37] found *COL15A1* transcripts were lower in DD samples which is the opposite to our results. However, Satish et al (2008) were comparing DD and carpal tunnel syndrome derived fibroblasts and the differences between our studies may be due to the differences in our controls. Some past studies have also shown a higher level of expression of various collagen genes in DD [28], [31], [32], [35], [43], [47]. Our analysis indicates a number of other collagens that showed higher levels in DD samples compared to controls. However, it is possible that some of the highly expressed collagen transcripts (e.g. *COL15A1*) are binding to the similar PSR sets for other collagen genes which would then also manifest as increased in DD patients.

Modulated expression levels in matrix metalloproteinase (MMP) genes is a common finding in previous studies although results vary depending on the experimental design [29], [31], [33], [36], [49]. Various MMP genes have been shown to have a higher expression level in DD and a few others have been shown to have lower expression. For example, *MMP2* has previously been shown to have a higher expression in DD [28], [29]. We found a decrease in the expression level of *MMP16* in DD. MMP16 protein activates MMP2 protein which in turn degrades type III collagen. We also found a substantial decrease in *MMP1* gene expression in DD compared to the gene expression of control fibroblasts obtained from patients with no signs of DD. However, Johnston et. al. (2007) [33] found a higher expression level of *MMP1* in DD compared to carpal tunnel syndrome tissue samples. MMP1 protein functions as an interstitial collagenase to break down interstitial collagen types I, II and III. The expression levels of the *MMP3* (stromelysin) gene, which codes for a protein that is able to activate the MMP1 protein [54] was also down in DD cells. Rehmen et al (2008) [31] who compared DD and carpal fascia tissue (from patients with carpal tunnel syndrome), also found a decrease in expression level of MMP3 in DD. We speculate that a low level of activated MMP1 proteins in DD may cause an accumulation of type I, II, and III collagens in the ECM due to an inability to break them down. In addition, low levels of MMP16 protein may decrease the activation of MMP2 and increase the build-up of collagen type III.

These findings provide compelling evidence that the development and progression of DD is closely associated with significant up-regulation of a broad group of collagen genes and down-regulation of matrix metalloproteinase and other collagenase genes which are required in remodelling the ECM. They also extend the understanding of the likely genetic origins of DD and provided the experimental rationale for the recent use of injectible collagenase from *Clostridium histolyticum* in the non-surgical treatment of DD which has been found to be effective in controlling DD despite the associated pain tolerated by patients [44]. A twelve month follow-up study of this treatment indicated that some patients had debilitating pain and deep tissue scarring and adhesion [55]. Longer term studies are now required with this treatment to examine its effectiveness in preventing recurrence of DD and also to assess any negative consequences or non-specific effects of the treatment. More specific collagenases such as active MMP1 and MMP2 proteins may be better candidates for therapeutic treatment.

Other ECM components may also be involved in DD. Our findings show that transcripts of cathepsin K (*CTSK*), a lysosomal cysteine proteinase involved in bone and possibly ECM remodelling and resorption, are also lower in DD samples. Other matrix remodelling genes such as plasmin-mediated matrix remodelling protein, tissue factor pathway inhibitor 2 (*TFPI2*) and *TFPI* transcripts were also expressed at significantly lower levels in DD samples. BMP4, which has increased transcription levels in DD fibroblasts, induces cartilage and bone formation but also has been shown to regulate tissue remodelling and fibrosis [56]. The proteoglycan gene *PRG4* was found to have higher expression levels in DD compared to controls which is consistent with other studies that have investigated fibroblasts from DD patients [36], [37]. This proteoglycan prevents protein deposition on to cartilage but its function in DD remains unclear.

Not all gene transcripts associated with the ECM showed higher levels in DD. For example, two fibronectin genes, fibronectin type III domain containing 3A (*FNDC3A*), and fibronectin leucine rich transmembrane protein (*FLRT2)* showed lower levels in DD fibroblasts. There was also a down-regulation of transcripts from laminin 4 alpha gene (*LAMA4*). Laminin 4 alpha is part of laminin 411 which is found in endothelial basal laminae and is believed to up-regulate insulin gene expression [57]. This gene may reflect the high incidence of diabetes in DD patients [58]. Another gene that has been associated with type II diabetes and shows higher gene transcript levels in DD, is angiopoietin-like 4 (*ANGPTL4*), a glycosylated, secreted protein with a fibrinogen C-terminal domain involved in glucose homeostasis, lipid metabolism and insulin sensitivity [59]. It inhibits proliferation, migration and tubule formation in endothelial cells and is induced and accumulates in the ECM in response to hypoxia.

Our data indicate an increase in cell-to-cell interaction and dysfunction in the regulation of cytoskeletal structure. Many transcripts from genes involved in cell adhesion are found at a higher level in DD. For example, the vascular cell adhesion protein, (*VCAM1*) cell adhesion molecule 1 (*CADM1*), chitinase 3 like 1 (*CHI3L1*), neuronal cell adhesion molecule (*NRCAM*), and thrombospondin 4 (*THBS4*). As vascular cell adhesion gene (*VCAM1*) has an important function in cell-cell recognition and thrombospondin 4 is an adhesive glycoprotein that can bind fibrogen, fibronectin, laminin and type V collagen, an increase in these proteins would increase the amount of adhesion between the cells as well as with the ECM. There are also lower transcription levels in DD of the podocalyxin-like gene (*PODXL*), a negative regulator of the cell adhesion. These finding suggest that genes promoting cell adhesion are increased in the development and progression of DD.

ADAMs proteins are active metalloproteinases with gelatinolytic and collagenolytic activity. They inhibit beta-1 integrin mediated cell adhesion and migration. The ADAMs suppress cell mobility, cleave E-cadherin in response to growth factor depletion and may be active in cartilage remodelling. We found gene transcripts of these proteins, which increase cell adhesion and decrease cell mobility, are increased in DD as in previous studies [34]. Integrins are also involved in cell adhesion and participate in cell-surface mediated signalling [60]. The integrins (ITG) gene transcripts were found to be both higher and lower in DD. For example, *ITGA11* transcripts were found at higher levels in DD whereas *ITGA2*, *ITGA6*, and *ITGA4* gene transcripts were lower in DD. Integrin alpha 11 cell surface adhesion receptor is involved in cell adhesion to the ECM and to other cells. The levels of this gene are increased in DD possibly increasing adhesion of cells and ECM in DD. Integrin alpha-2/beta-1 is a receptor for laminin, collagen, fibronectin and E-cadherin and is responsible for adhesion of cells to collagen, modulation of collagen and collagen gene expression, and organization of newly synthesized ECM. The levels of *ITGA2* transcript, which encodes for integrin alpha 2, are down in DD which may cause a disorganisation of collagen.

Many of the gene transcripts that are lower in DD are involved in cytoskeletal structures (cytoskeleton associated protein 2 (*CKAP2*)), microtubule-based movement (KIF family), spindle formation (e.g., TPX2), centromere proteins such as kinetochores (*NUF2*, *CENPF*), and chromosome condensing (*NCAPG*). Lower levels of transcripts in these genes may reflect differences in proliferation between the two sample groups, however, there is also a lower level of Rho-associated genes that are involved in cytoskeletal rearrangement and cell motility. This includes SEMA3A, a protein possibly involved in cytoskeletal organisation, and indicates a possible association between DD and cytoskeletal structure.

We were interested to determine if follistatin and activin were involved in DD disease. Follistatin has been shown to antagonise fibrosis by complexing with activin [61] and to modulate the proinflammatory and profibrotic actions of activin during wound healing, tumourigenesis [62], [63] and in rats treated with bleomycin, an agent that causes DNA double-strand breaks [64]. When we analysed the differences in follistatin and activin subunit gene expression, we observed that levels of follistatin were much lower in the DD samples, which is consistent with the proposed anti-fibrotic actions of follistatin [61]. Our study found unexpectedly that the levels of *INHBA*, which codes for the βA subunit of activin and is known to be involved with fibrosis, were also down-regulated. In contrast, expression levels of *INHBB*, which encodes for the activin βB subunit, were elevated in DD fibroblasts when compare with controls. However, the levels of *INHBB* transcript varied greatly between individuals, for both DD and control patients. Further, there is little data on the role of activin B in the modulation of fibrosis as assays for this protein have only become available recently [65]. *In vitro* studies are required now to further explore the relationship between follistatin, activins and collagen synthesis in DD fibroblasts because of the potential for follistatin to be used as a novel treatment for DD.

Two fibroblast growth factor genes (*FGF9 and FGF11)* were significantly up-regulated in DD fibroblasts. The FGF family of genes encode for mitogens and proteins involved in cell survival and various cell processes. Up-regulation of these growth factors in DD fibroblasts links with the increase in fibroblast proliferation and fibromatosis in DD. The proteins encoded by these genes are members of the fibroblast growth factor (FGF) family and are implicated in the stimulation of cell growth and tissue repair. The protein encoded by *FGF9* was isolated as a secreted factor that stimulates growth in cultured glial cell but the exact functions of both *FGF9* and *FGF11* on fibroblasts, and particularly those from DD patients, have yet to be determined (http://www.ncbi.nlm.nih.gov/gene/2256). Platelet-derived growth factors have been recently implicated in DD [66], [67] and have a specific effect on angiogenesis. However none of the three PDGF genes (*PDGFB, PDGFC, PDGFD*) examined in this study were up-regulated in DD fibroblasts suggesting that enhanced angiogenesis is not a critical factor in the establishment of DD.

Other genes up-regulated in DD are also involved in inflammatory diseases. For example, tumour necrosis factor, alpha-induced protein 6 (*TNFAIP6*), which is found in the synovial fluid of patients with osteoarthritis and rheumatoid arthritis was up-regulated in DD. Vascular cell adhesion gene (*VCAM1*), which may play a role in atherosclerosis and rheumatoid arthritis, showed a 30-fold increase in expression in DD. VCAM1 also has an important function in cell-cell recognition. However another gene that codes for a protein involved in the innate immune response, STAT1, had a lower expression level in DD patients which may reflect the lack of inflammation observed in DD.

There is also up-regulation of a suite of keratin genes, particularly *KRT34,* in DD fibroblasts. Keratin is a protein usually involved in the formation stratified squamous epithelium and hair and is particularly associated with keratinocytes in the skin. Currently the nature of this relationship between up-regulated expression of *KRT* genes in DD fibroblast and DD is unclear.

Application of an alternative splicing algorithm to our exon array data revealed a number of gene isoforms that appear to have different levels between DD and control fibroblasts. These included *THBS4*, *KRT34*, *TIRAP*, *KIF14* and *KIF23*. There is approximately a 12 fold increase in the *THBS4* gene transcript in DD patients but only for part of the gene. The data suggests an increase in a transcript that codes for a protein that is missing approximately the first 92 aa of the N-terminus. As the N-terminus of this protein binds to heparin (possible binding site between aa 102 and 105) [68]; this protein may have an altered heparin binding capability but would probably still be involved in cell-matrix interactions. As there is an increase in KRT34 gene in all but the first PSR (first exon) in DD, the increased *KRT34* transcript codes for a protein missing approximately the first 43 aa of the N-terminus. The *TIRAP* transcript increased in DD patients is missing the 5′UTR of the gene. Although the resulting protein would be unchanged, the stability of the transcript may be altered. The expression profiles of both *KIF14* and *KIF23* indicate that the regions corresponding to exon 1 in both genes are unchanged, but all the following PSRs are decreased in DD fibroblasts. Transcripts in these unchanged regions may be protected from RNA degradation that is occurring in DD or premature termination of transcription is occurring at this point. A similar profile of decreased transcript expression with protection of the first exon was found 4 hours following 10 Gy ionizing radiation of fibroblasts in both *KIF14* and *KIF23* [69]. Recently published papers examining the response of fibroblasts to ionizing radiation found that a common general response mechanism to that stress is the use of alternative start sites [69], [70]. The disease state in DD could reflect a defect in a stress response leading to alternative isoforms that have substantial effects on the normal production of the ECM. Alternatively, lower oxygen levels may be present in the zone of the affected region of the hand, which may induce alternative transcripts.

Recently, a large study that looked at SNPs in 1365 DD patient bloods identified a number of WNT gene SNPs as being associated with DD patients [41]. One of the SNPs was in *SFRP4*, a frizzle-related gene which was at the top of our list for genes that are more highly expressed in DD compared to normal samples. However, this SNP was more closely associated with the gene *EPDR1*, a gene involved in cell adhesion, which we found had an increased expression in DD. These results provide intriguing clues to the cause of DD disease.

An advantage of our study was the use of control samples from donors with no DD genetic background. This avoided the potential complication of associated genetics in control samples from DD-affected tissue donor which have been used as controls in some previous investigations. We also had the advantage that highly sensitive exon arrays were used to obtain quality results. In this study we have not only identified transcripts which are precursors to known fibrotic components, but also have identified a large number of potential DD treatment candidates, some of which have also been identified in other genomic studies. Some of our findings, however, also contrast with and contradict those of other studies and require further examination to discovery how they relate to the onset and progression of DD.

In conclusion, we have comprehensively characterized the transcription profile differences between DD and normal primary fibroblasts. Our data indicate that in DD there is an excess of collagen and other ECM that is not controlled due to a reduction in matrix metalloproteinases and other matrix remodelling proteins. A reduction in the fibrotic control protein, follistatin, may also contribute to DD. In addition, the fibroblasts lack expression of genes involved in cell movement and cytoskeletal organisation and an increase in genes involved in cell adhesion. These indicate a lack of organisation of both extra- and intra-cellular matrix as well as a lack of cellular movement in DD. Alternative transcripts have also been identified which are expressed at different levels in the DD patients compared to the controls and may reflect cell stress such as hypoxia. These conclusions will be the basis for future experimentation. Many of the identified genes are potential candidates for the treatment of DD. There was a close correlation between expression levels in some genes from our study and data from previous studies using DD tissue samples providing reason to pursue investigations into potential therapeutic development strategies using *in vitro* studies on DD fibroblasts. It is likely some of these candidate genes for treating DD will also be effective for fibrotic diseases in general, including injury-related and radiotherapy-induced fibrosis.

## Supporting Information

Figure S1 **Gene expression graphs across each exon for selected genes.** Graphs are as depicted in figure 2.
(PDF)

Figure S2 **αSMA staining of primary fibroblast cells.** Two control primary fibroblast cell lines, Control 1 and control 2, and 5 primary fibroblasts from DD patients were stained with αSMA (green) and counterstained with chromatin staining Hoechst (blue). Bar in picture is 50 micrometers long.
(PDF)

Table S1 **PCR primer sequences.**
(XLSX)

Table S2 **Genes that show higher gene expression in DD samples with a p-value of <0.05 and a fold change of >2.**
(XLSX)

Table S3 **Genes that show lower gene expression in DD samples with a p-value of <0.05 and a fold change of >2.**
(XLSX)
