# Supplementary material for: Genome-Wide Analysis Using Exon Arrays Demonstrates an Important Role for Expression of Extra-Cellular Matrix, Fibrotic Control and Tissue Remodelling Genes in Dupuytren's Disease
Source: PLoS One. 2013 Mar 12;8(3):e59056. doi: 10.1371/journal.pone.0059056 (PMC3595223; doi:10.1371/journal.pone.0059056)
Supplement: Figure S1 — Gene expression graphs across each exon for selected genes. Graphs are as depicted in figure 2. (PDF) [file pone.0059056.s001.pdf]

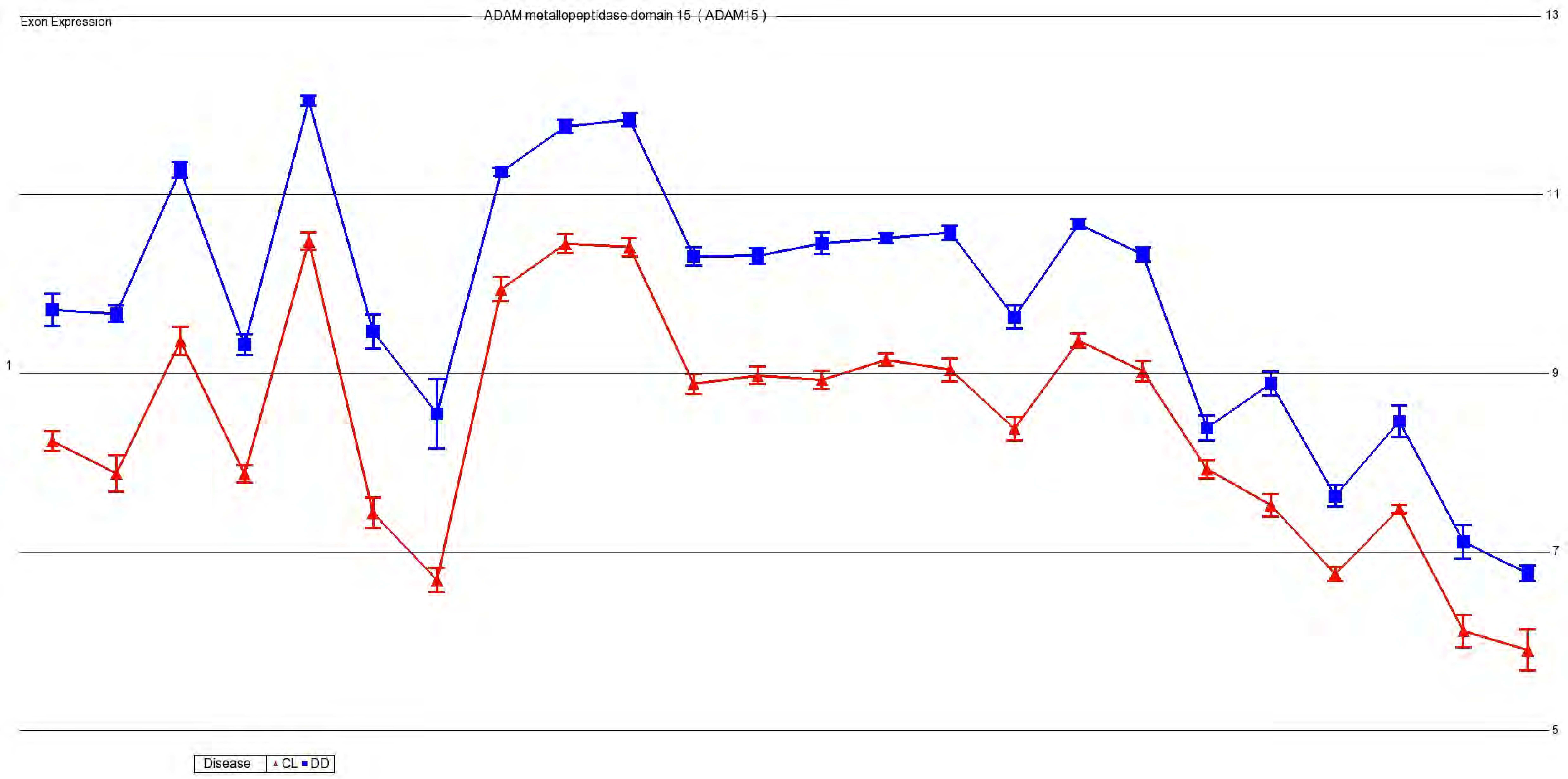

ADAM metallopeptidase domain 15 (ADAM15)
Exon Expression
13
11
9
7
5
1
Disease
CL
DD

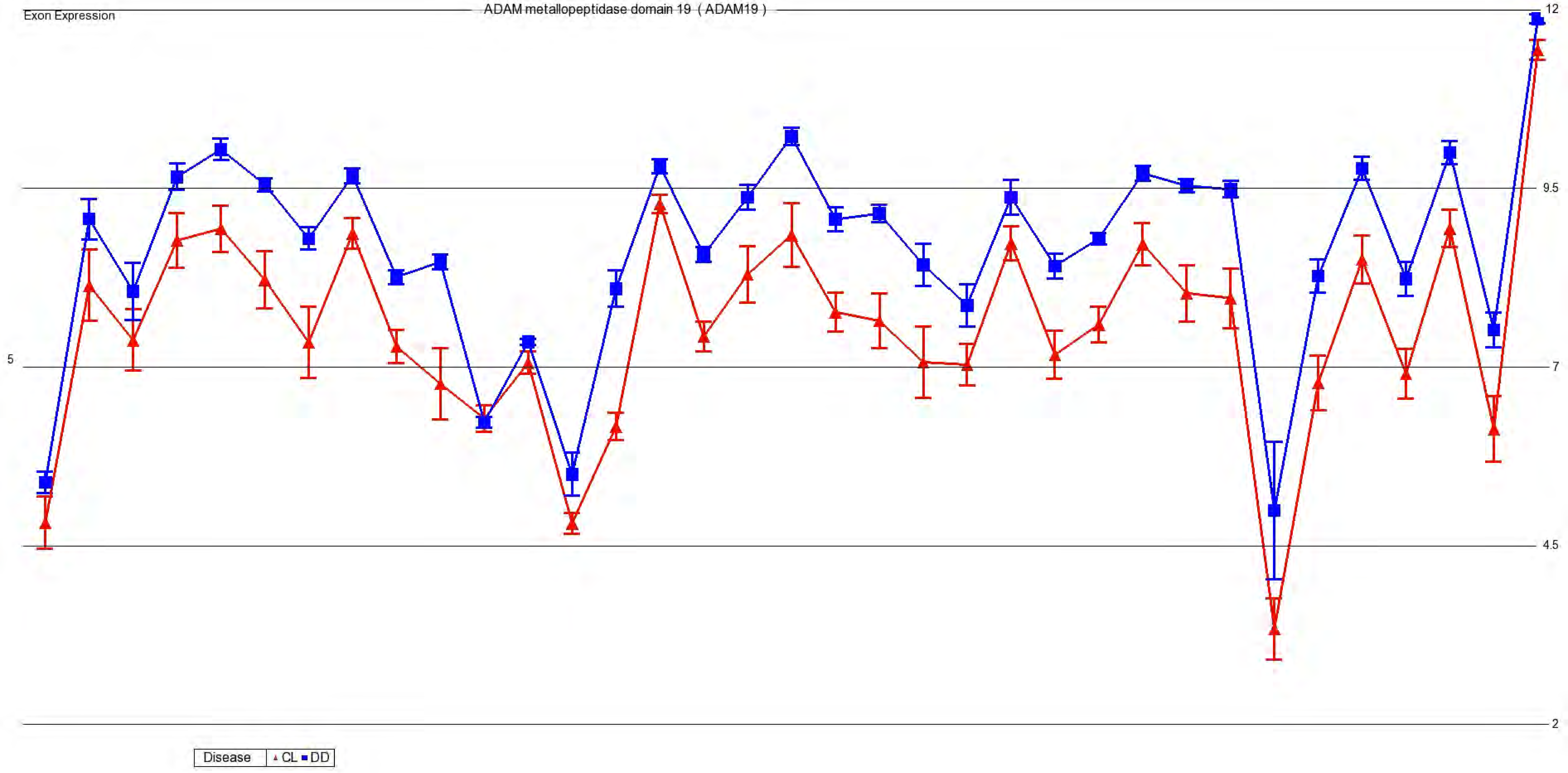

ADAM metallopeptidase domain 19 (ADAM19)
Exon Expression
12
9.5
7
4.5
2
5
Disease
CL
DD

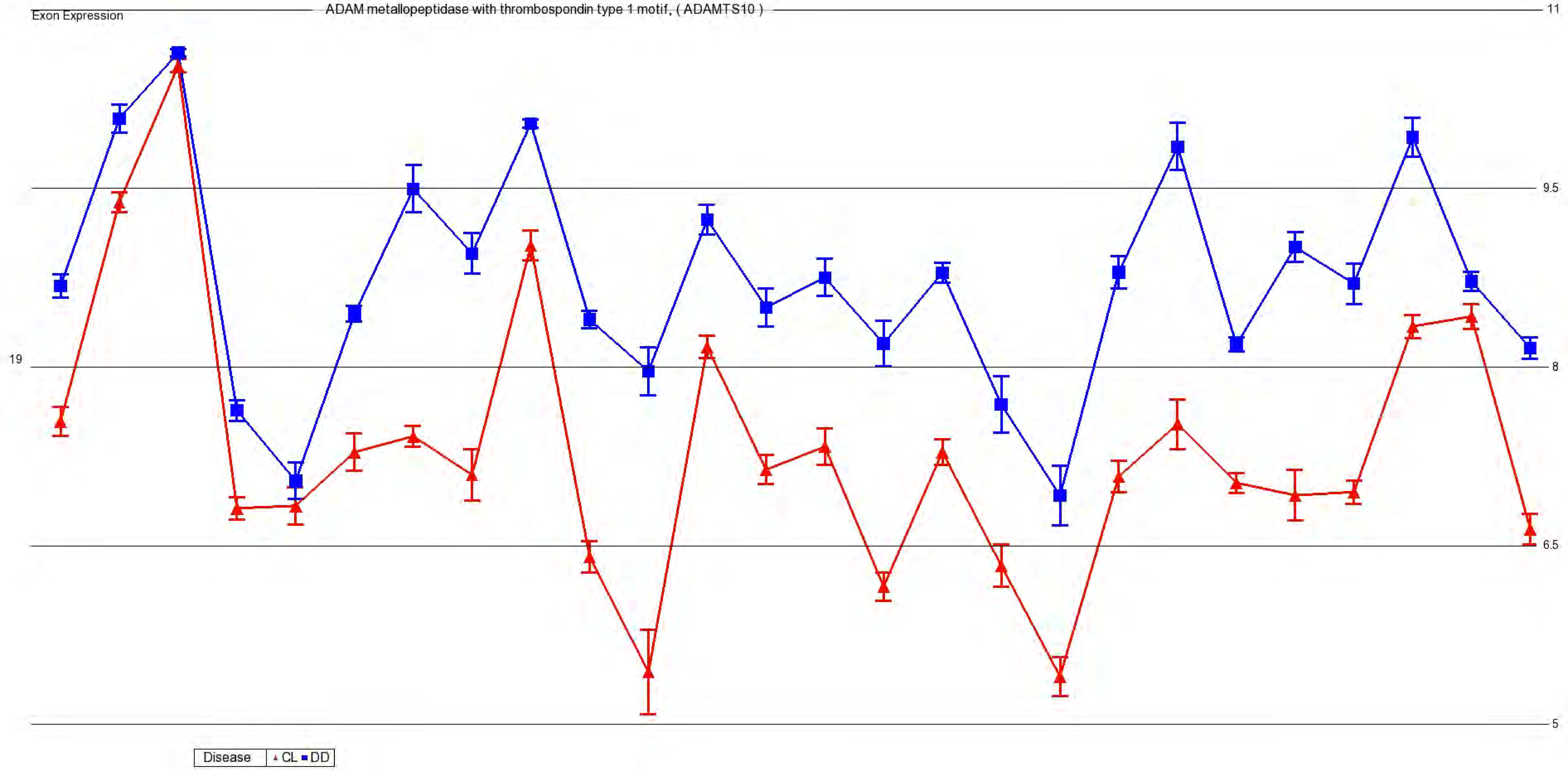
Exon Expression
ADAM metallopeptidase with thrombospondin type 1 motif, (ADAMTS10)
11
9.5
19
8
6.5
5
Disease
CL
DD

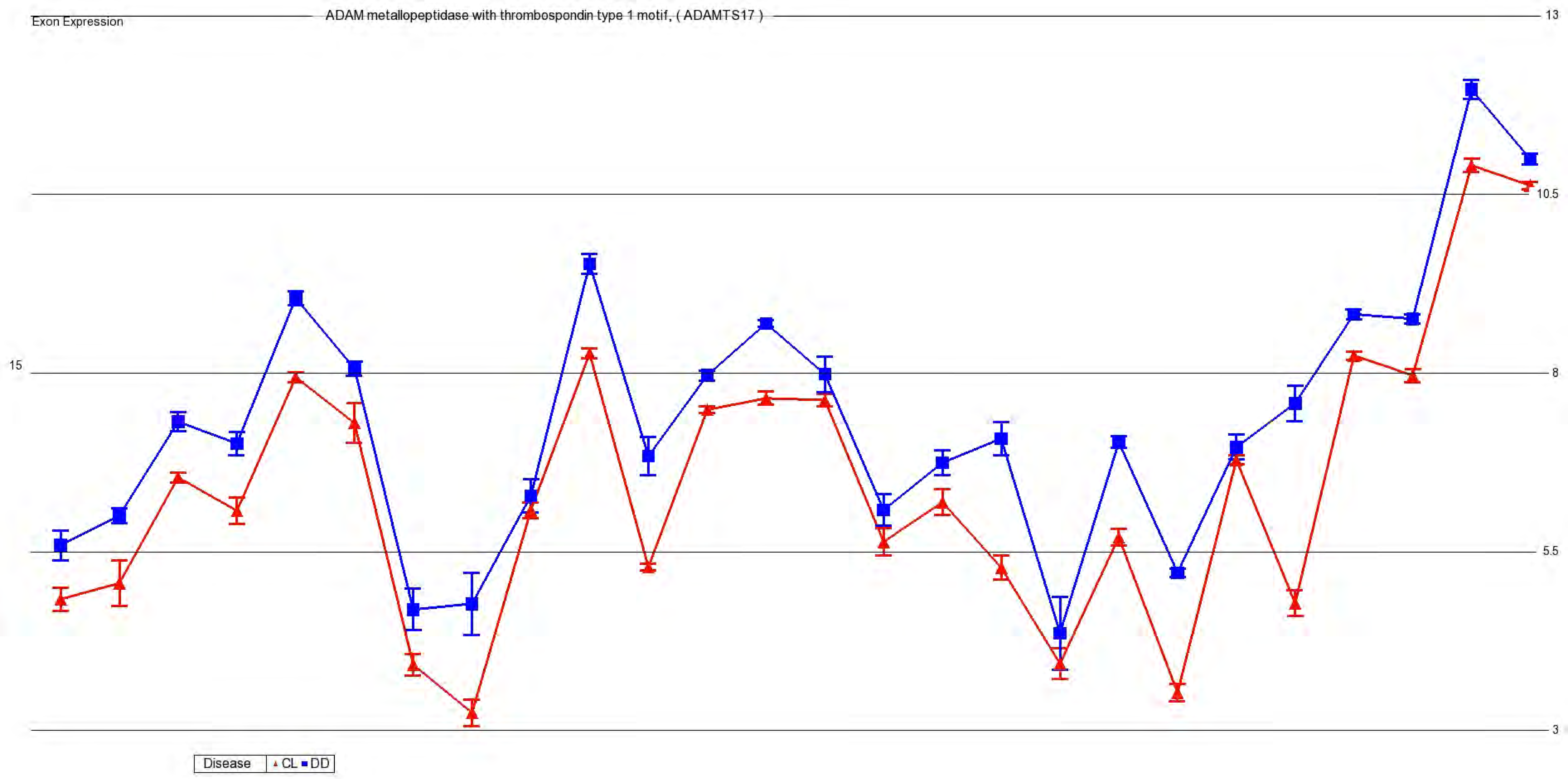

ADAM metallopeptidase with thrombospondin type 1 motif, (ADAMTS17)
Exon Expression
13
10.5
15
8
5.5
3
Disease
CL
DD

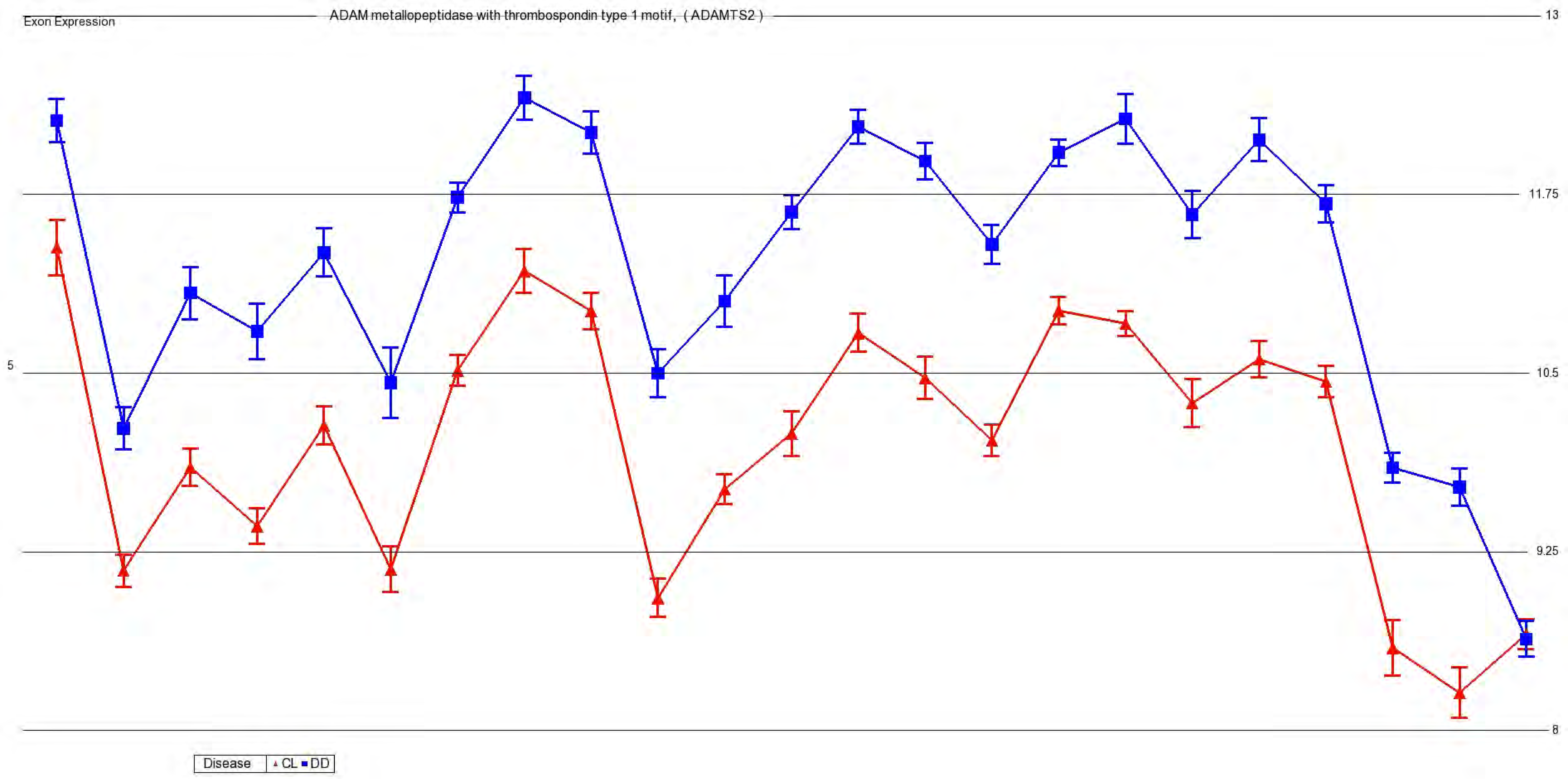
Exon Expression
ADAM metallopeptidase with thrombospondin type 1 motif, (ADAMTS2)
13
11.75
10.5
9.25
8
5
Disease
CL
DD

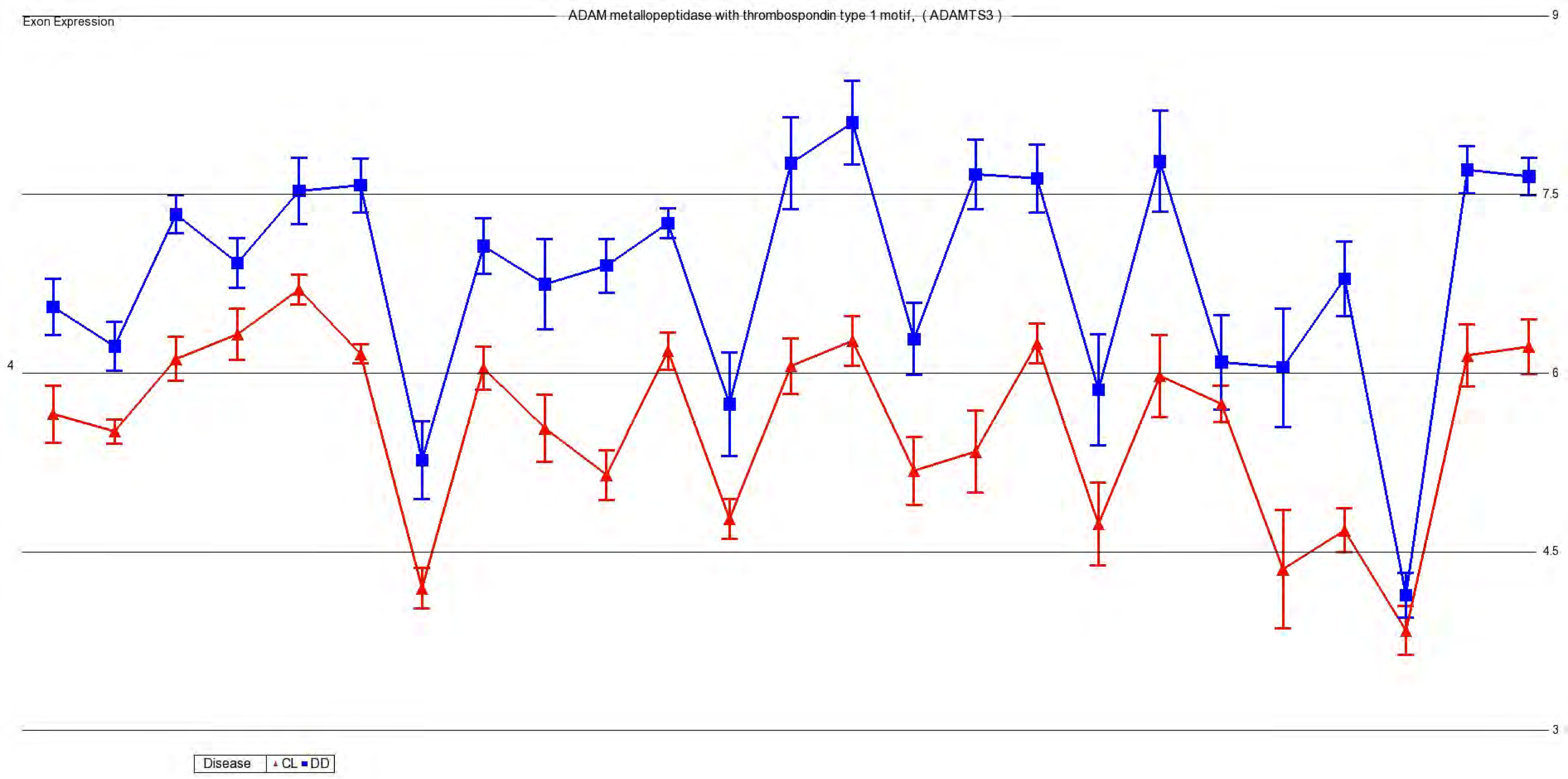

Exon Expression
ADAM metallopeptidase with thrombospondin type 1 motif, (ADAMTS3)
9
7.5
4
6
4.5
3
Disease
CL
DD

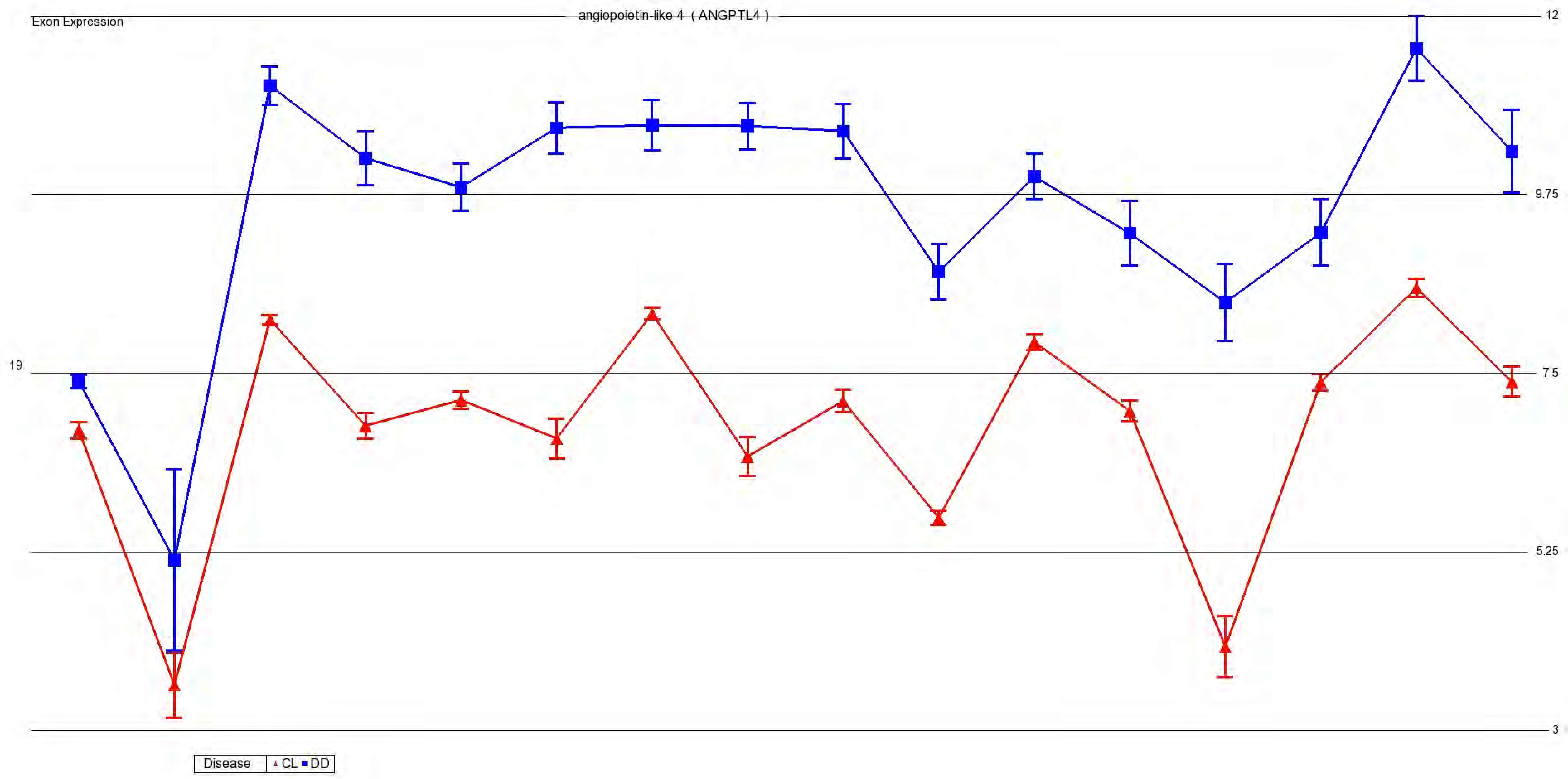

Exon Expression
angiopoietin-like 4 (ANGPTL4)
12
9.75
19
7.5
5.25
3
Disease
CL
DD

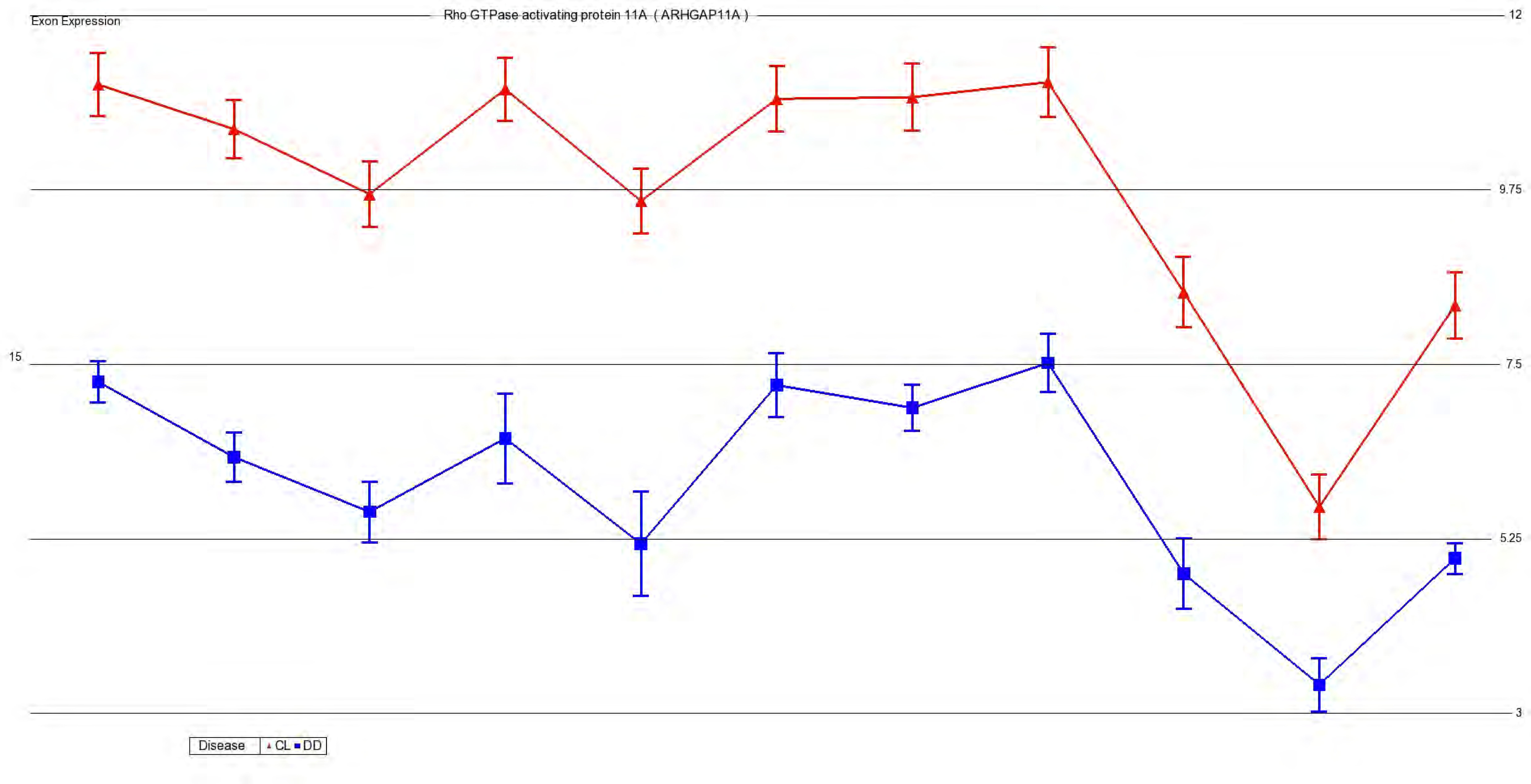

Exon Expression
Rho GTPase activating protein 11A (ARHGAP11A)
12
9.75
15
7.5
5.25
3
Disease
CL
DD

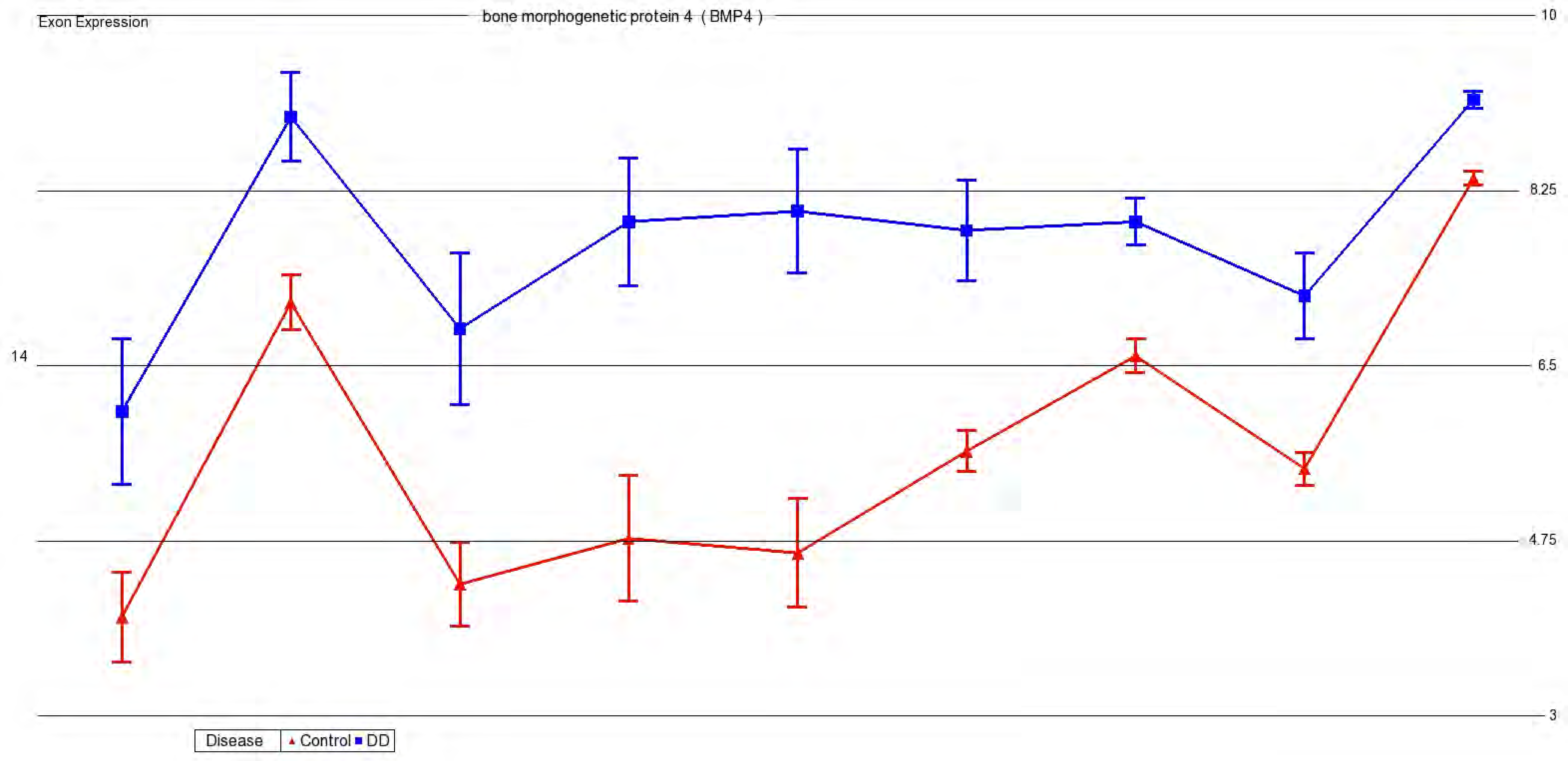

Exon Expression
bone morphogenetic protein 4 (BMP4)
10
8.25
14
6.5
4.75
3
Disease
Control
DD

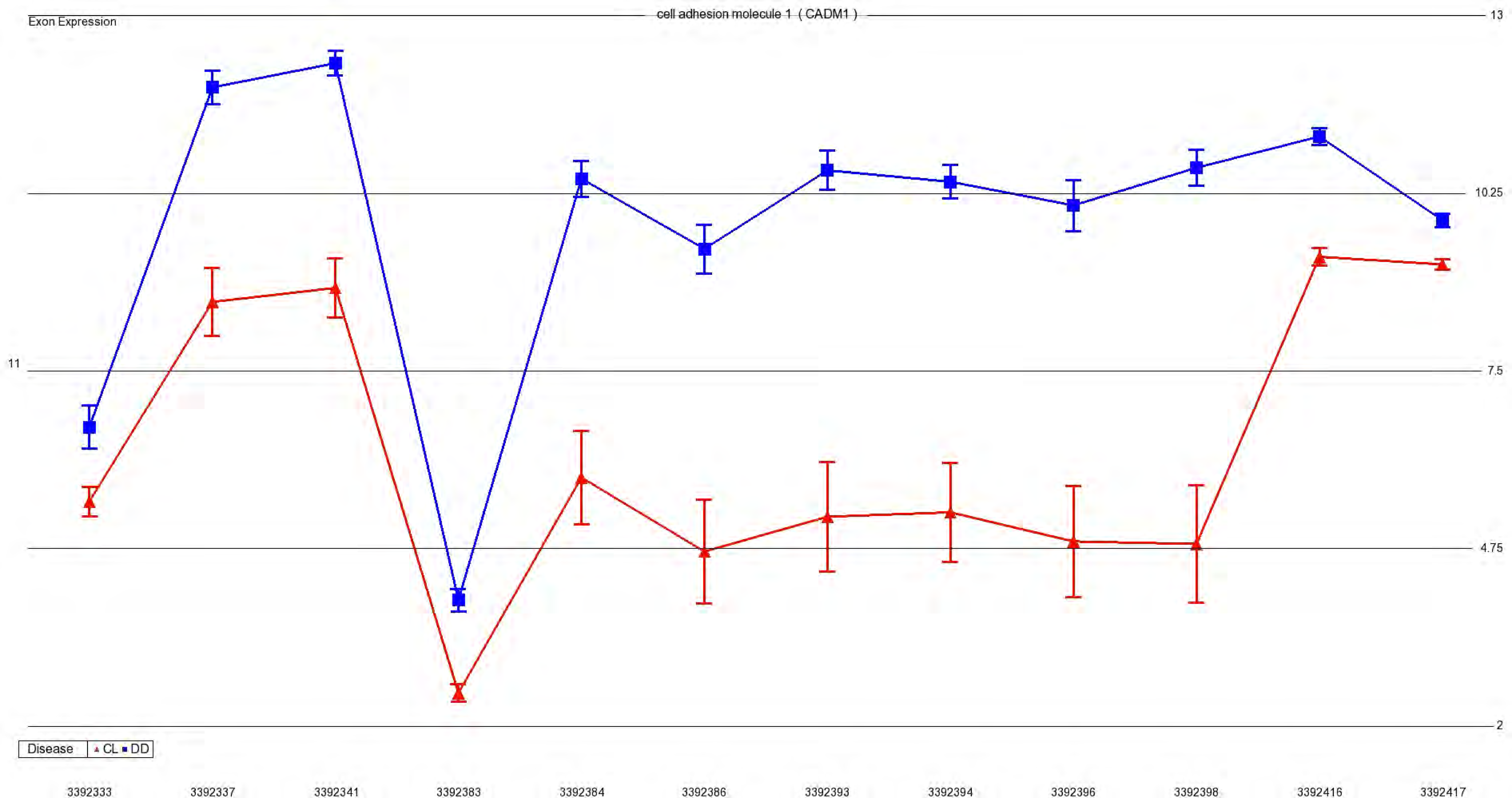

cell adhesion molecule 1 (CADM1)
Exon Expression
13
10.25
7.5
4.75
2
11
Disease
CL
DD
3392333
3392337
3392341
3392383
3392384
3392386
3392393
3392394
3392396
3392398
3392416
3392417

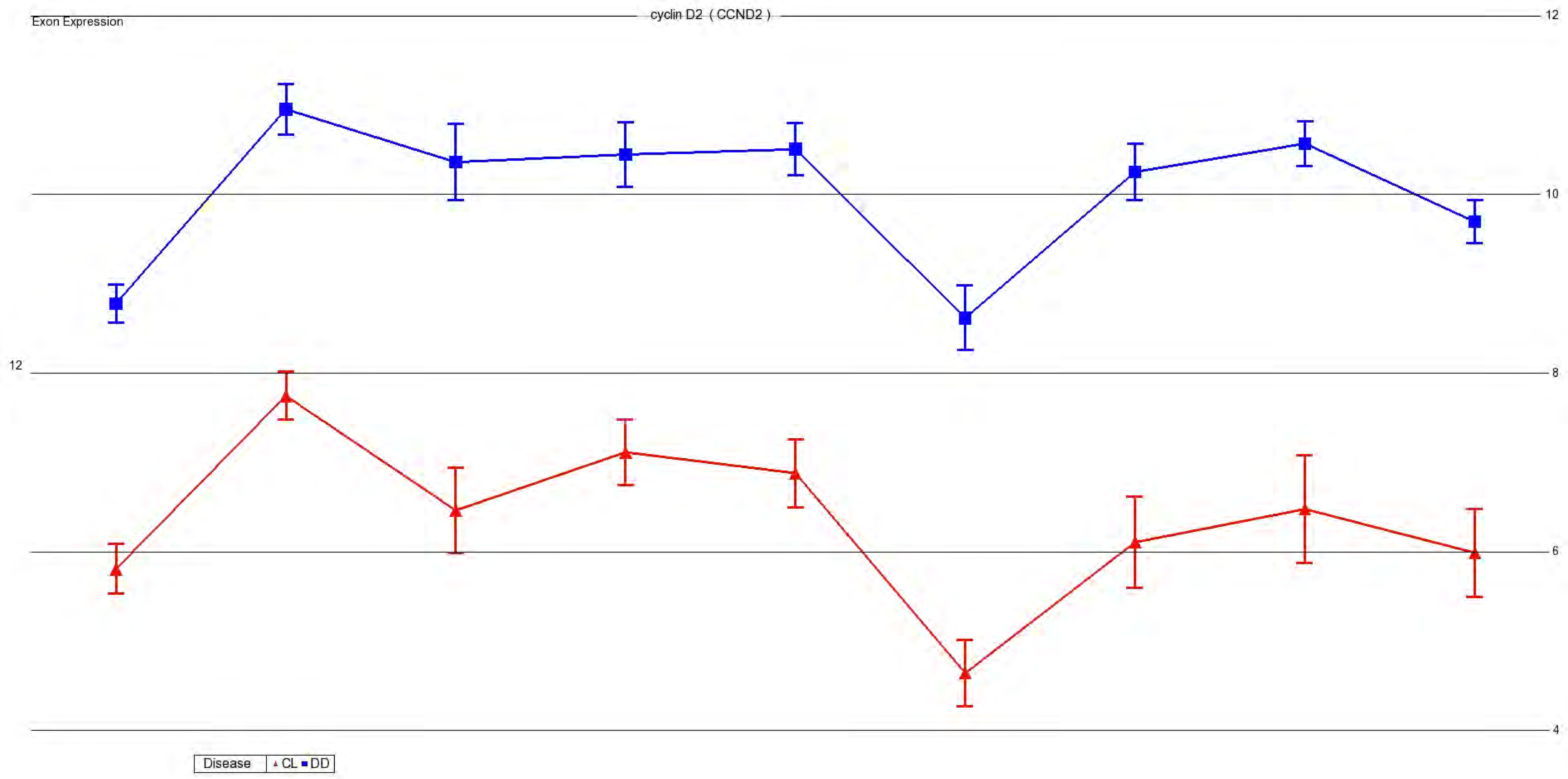

Exon Expression
cyclin D2 (CCND2)
12
10
8
6
4
12
Disease
CL
DD

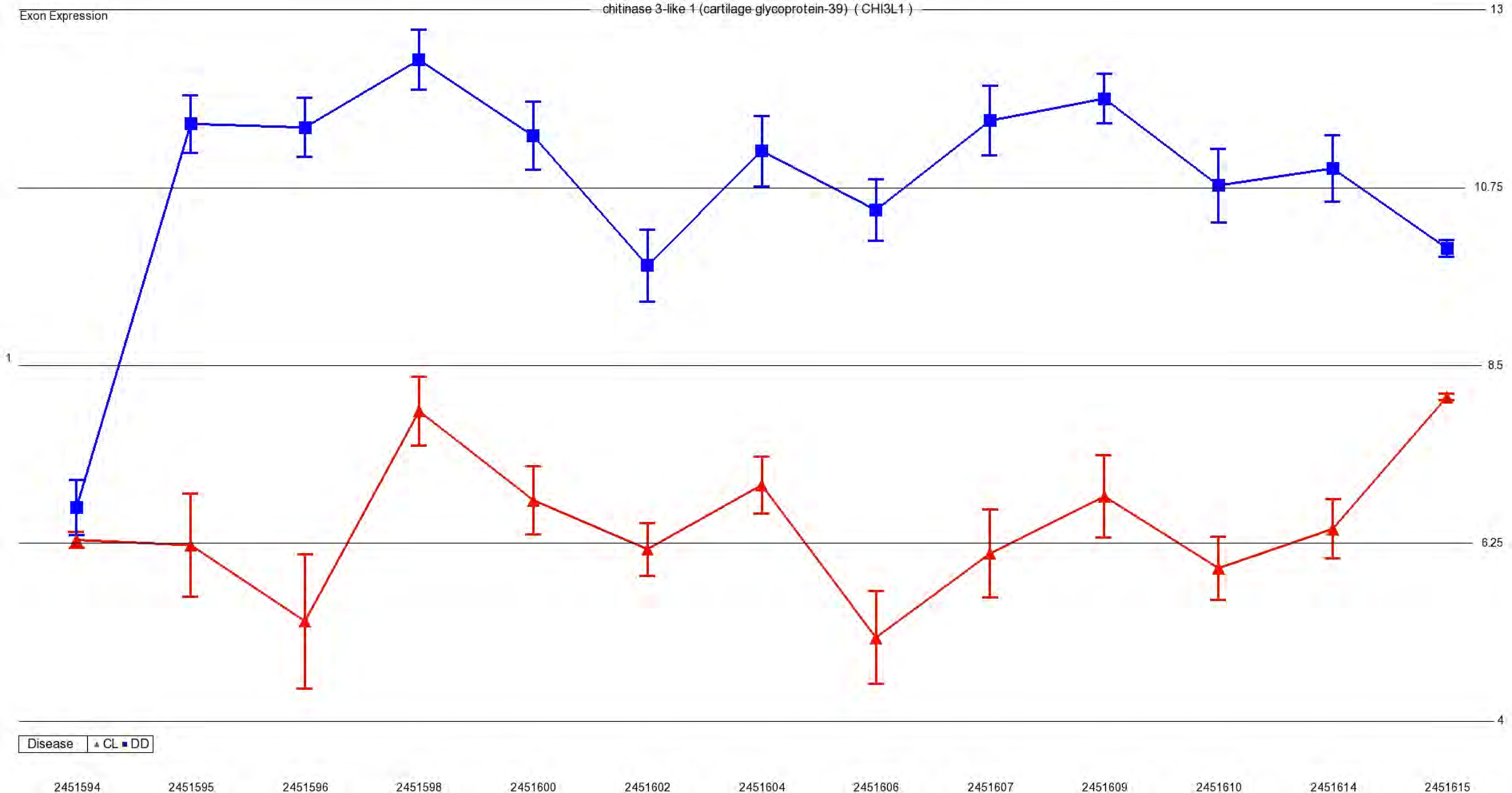

chitinase 3-like 1 (cartilage glycoprotein-39) (CHI3L1)
Exon Expression
13
10.75
8.5
6.25
4
1
Disease
CL
DD
2451594
2451595
2451596
2451598
2451600
2451602
2451604
2451606
2451607
2451609
2451610
2451614
2451615

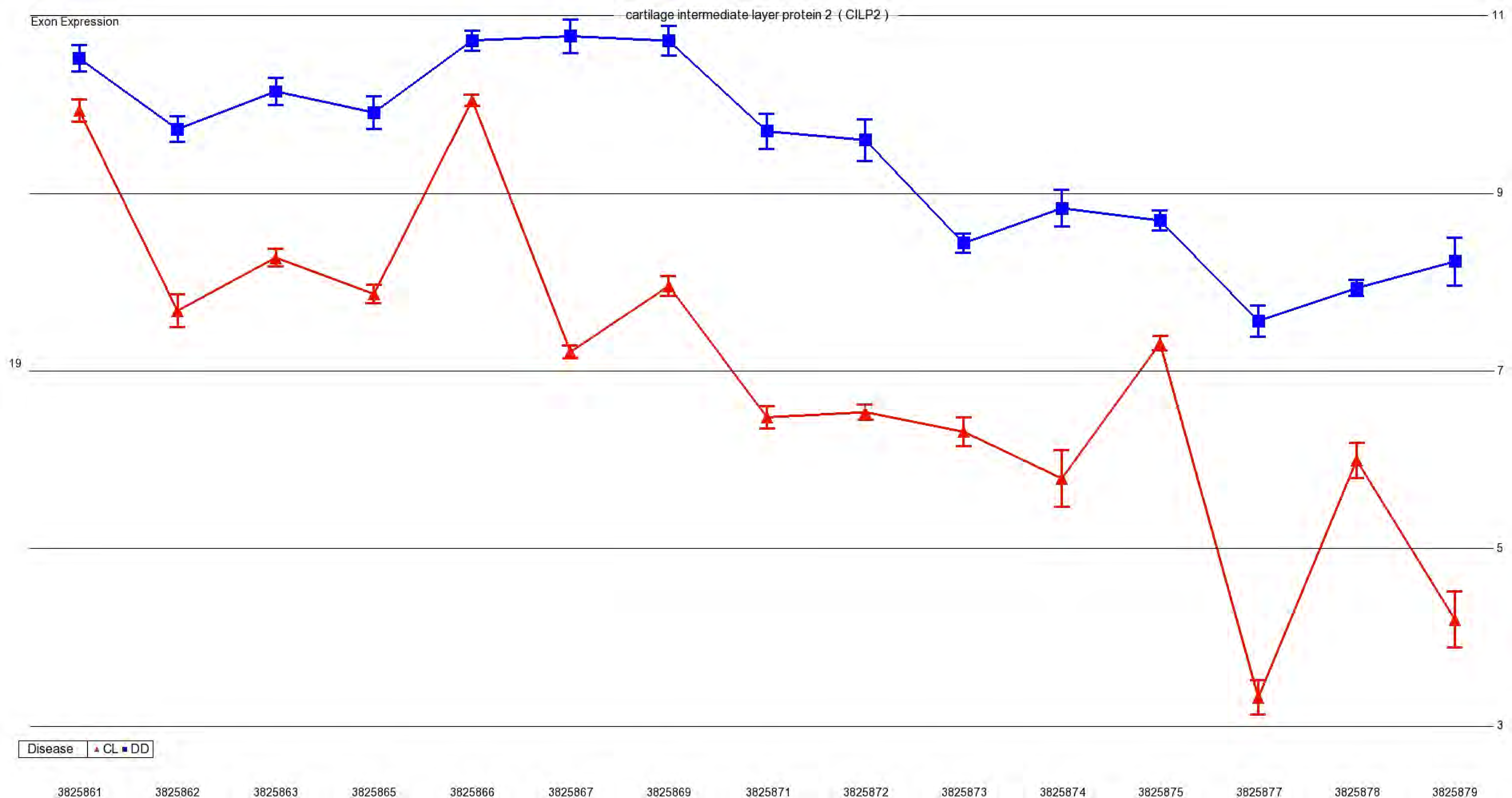
Exon Expression
cartilage intermediate layer protein 2 (CILP2)
11
9
7
5
3
19
Disease
CL
DD
3825861
3825862
3825863
3825865
3825866
3825867
3825869
3825871
3825872
3825873
3825874
3825875
3825877
3825878
3825879

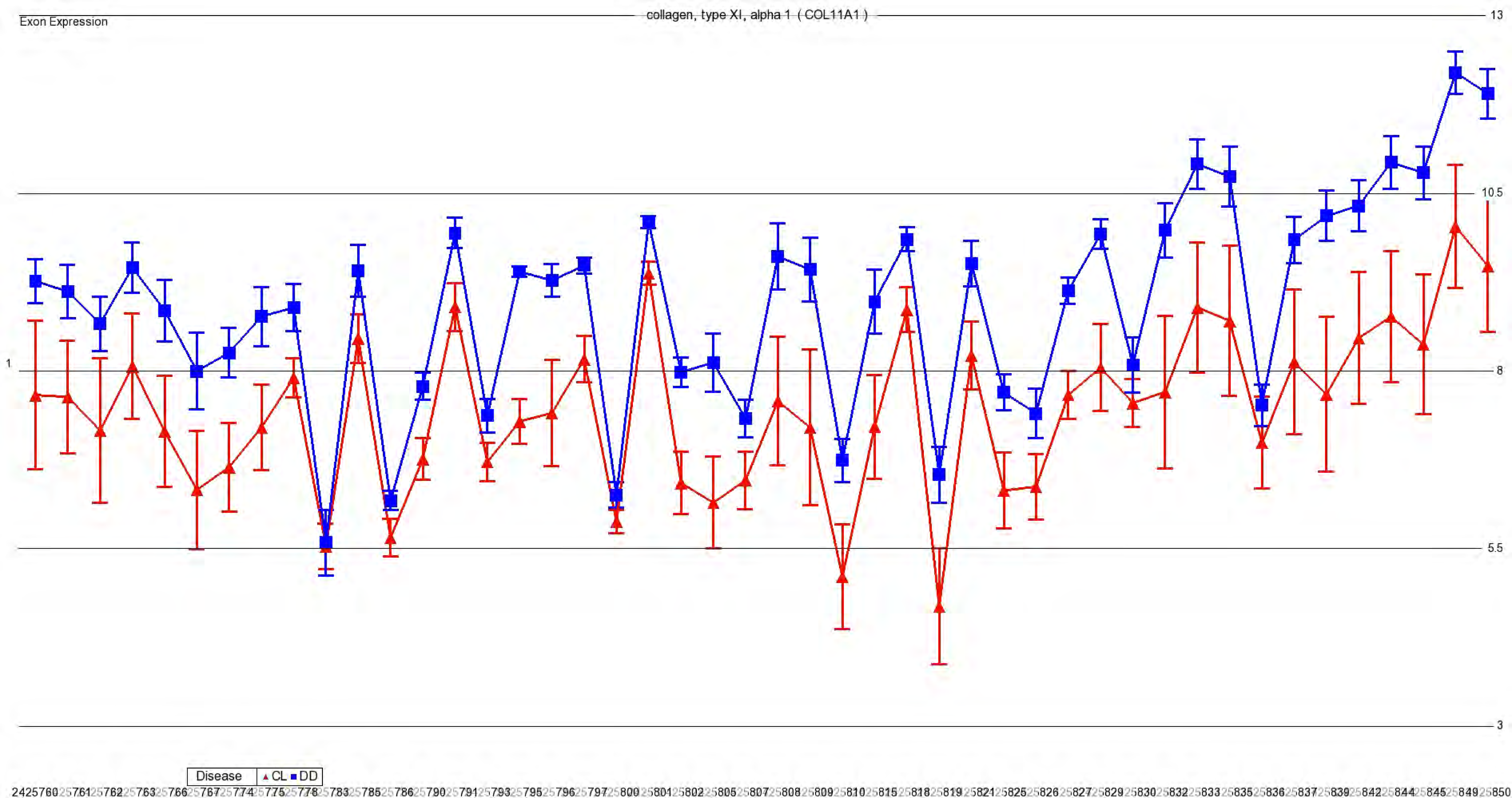

collagen, type XI, alpha 1 (COL11A1)
Exon Expression
13
10.5
1
8
5.5
3
Disease
CL
DD
2425760 25761 25762 25763 25766 25767 25774 25775 25778 25783 25785 25786 25790 25791 25793 25795 25796 25797 25800 25801 25802 25805 25807 25808 25809 25810 25815 25818 25819 25821 25825 25826 25827 25829 25830 25832 25833 25835 25836 25837 25839 25842 25844 25845 25849 25850

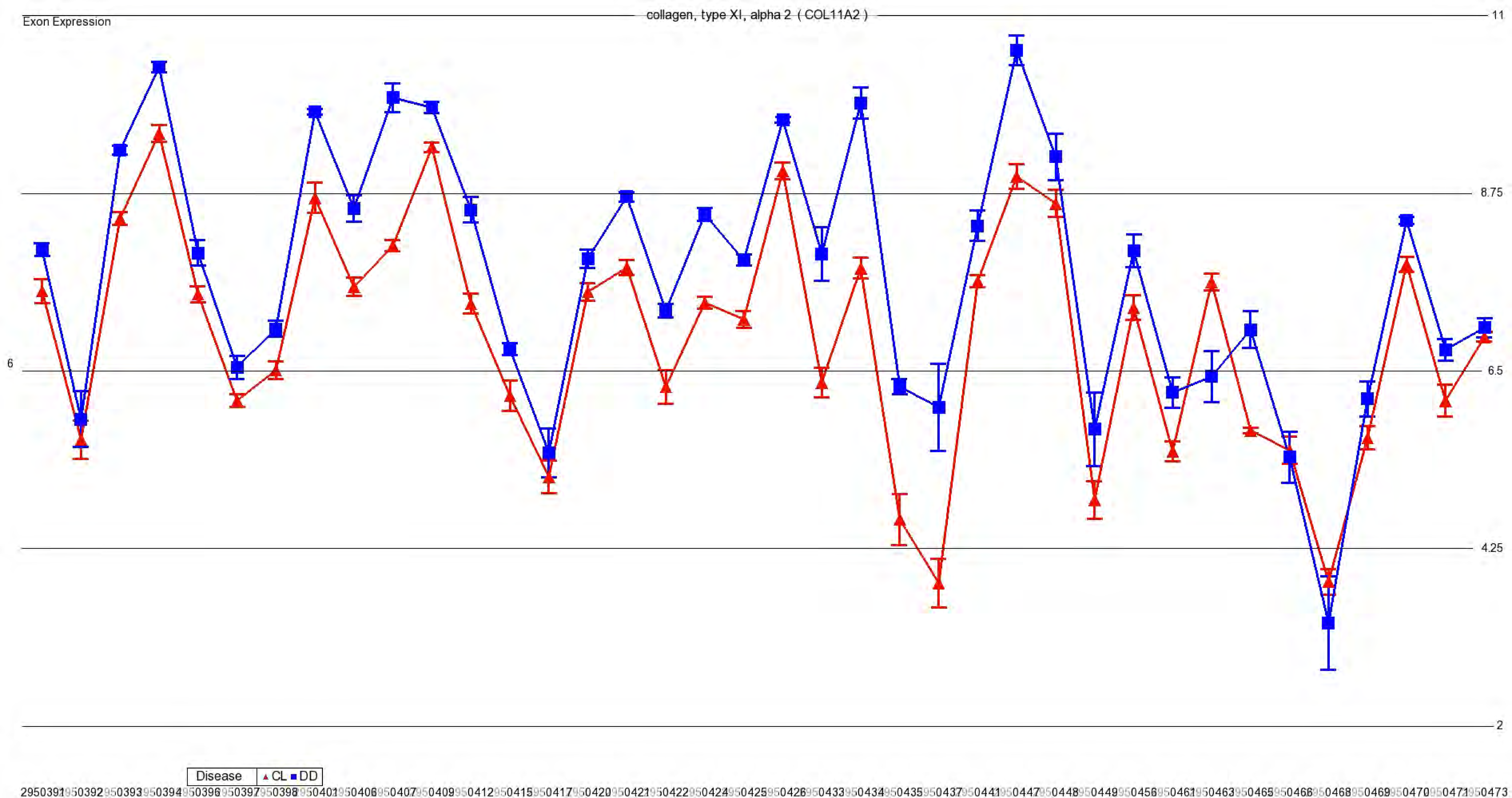

collagen, type XI, alpha 2 (COL11A2)
Exon Expression
11
8.75
6.5
4.25
2
6
Disease
CL
DD
2950391
50392
50393
50394
50396
50397
50398
50401
50406
50407
50409
50412
50415
50417
50420
50421
50422
50424
50425
50426
50433
50434
50435
50437
50441
50447
50448
50449
50456
50461
50463
50465
50466
50468
50469
50470
50471
50473

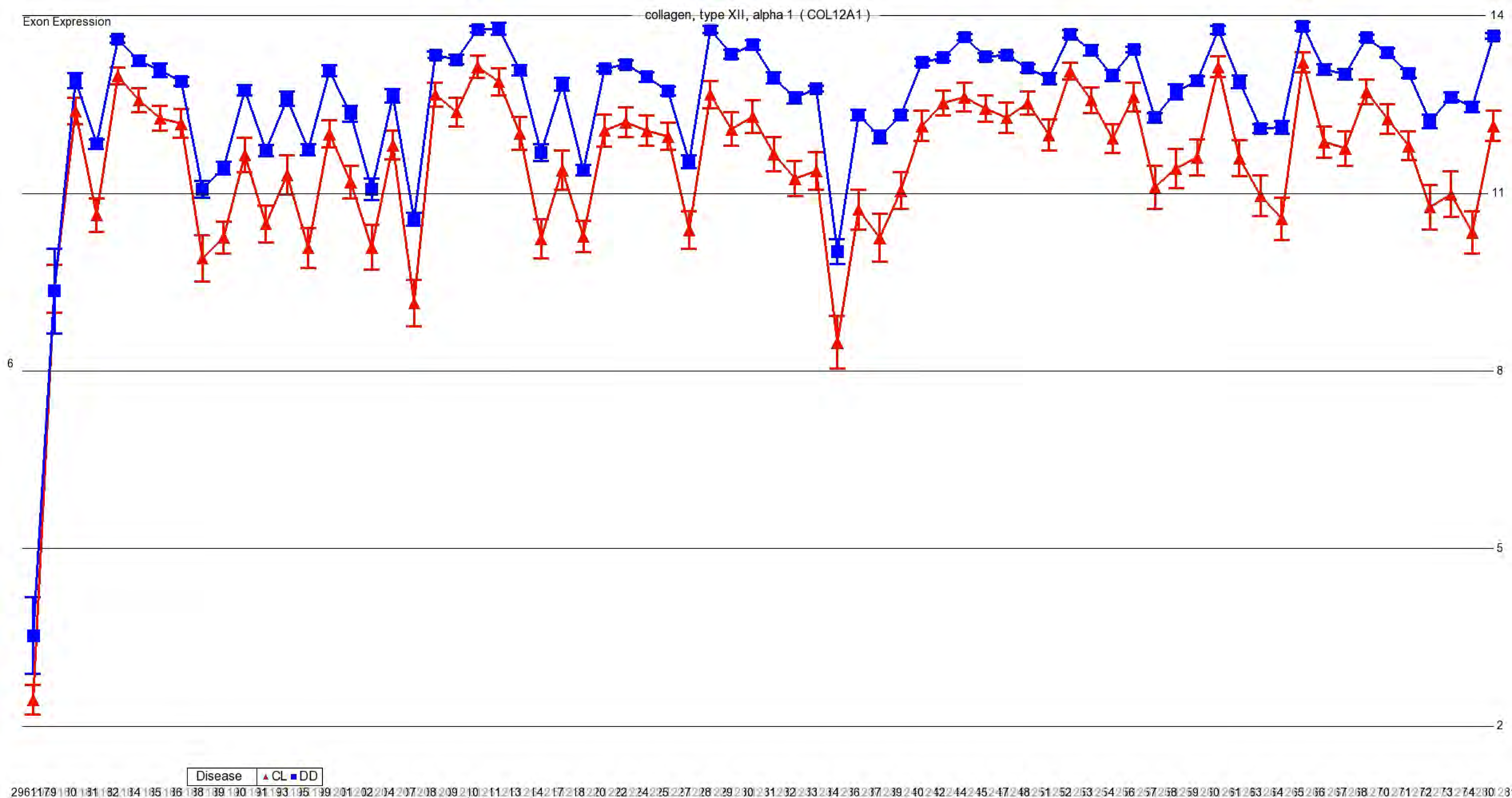

collagen, type XII, alpha 1 (COL12A1)
Exon Expression
14
11
8
5
2
6
Disease
CL
DD

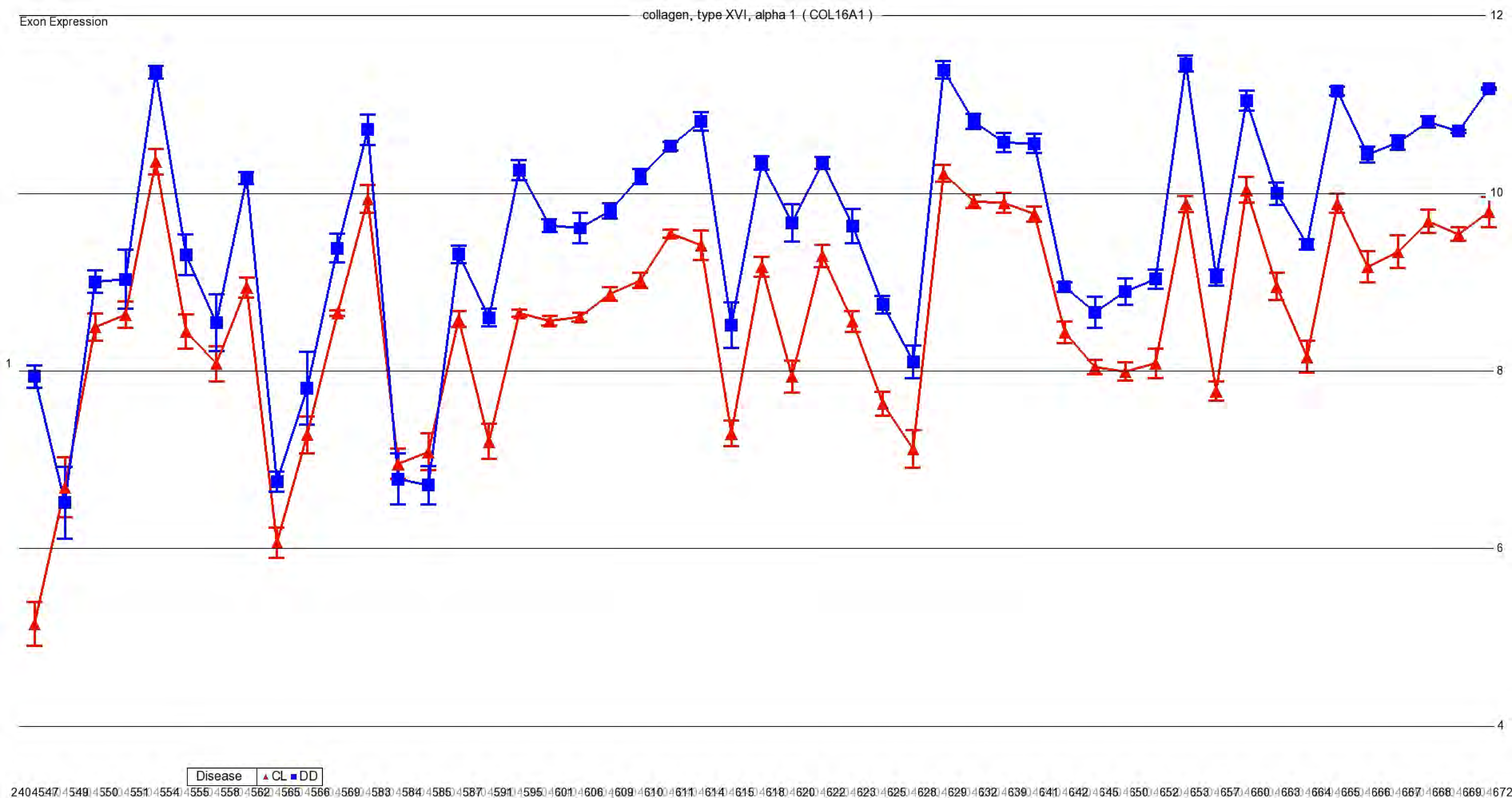
collagen, type XVI, alpha 1 (COL16A1)
Exon Expression
12
10
8
6
4
1
Disease
CL
DD

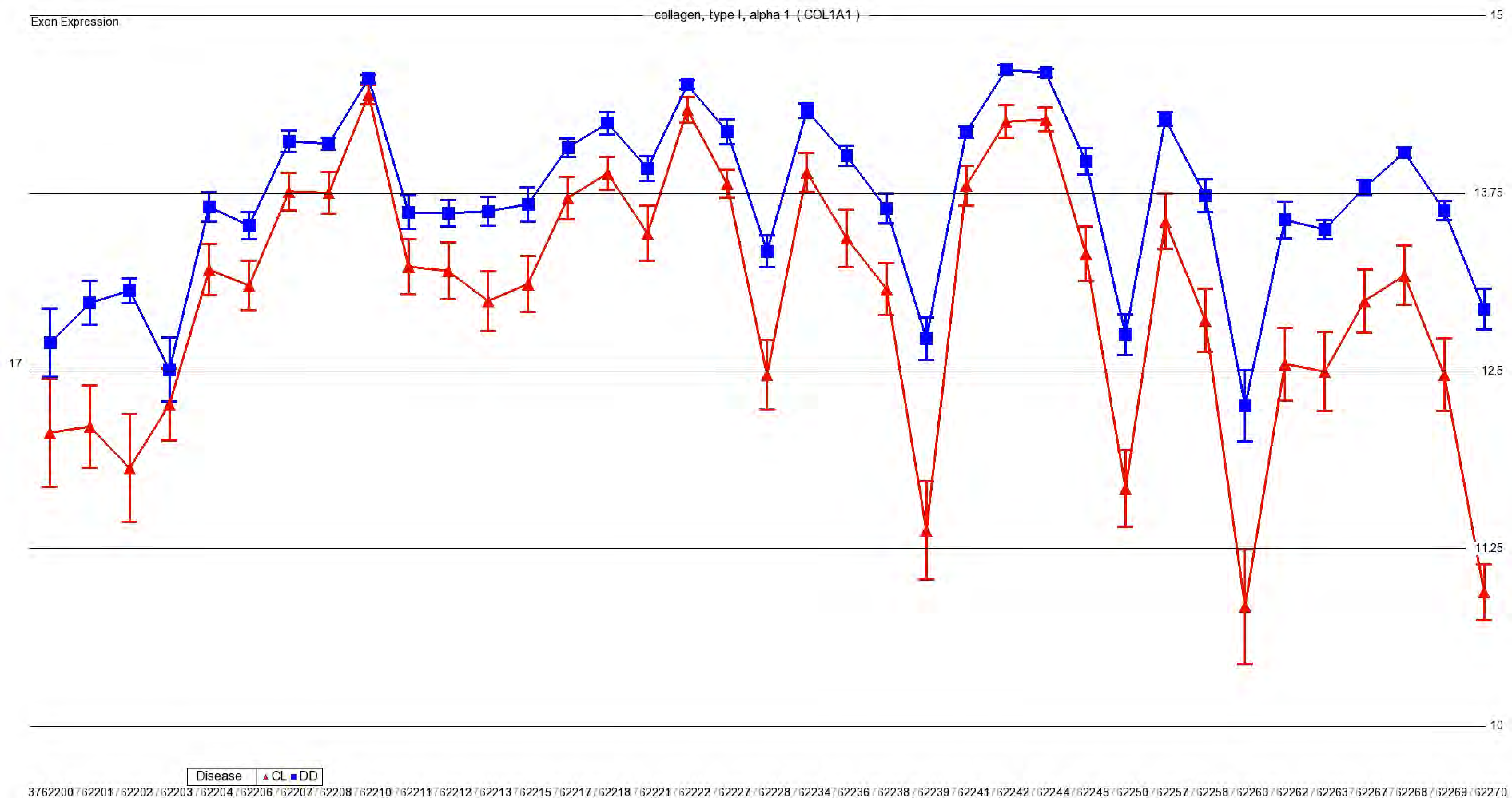

collagen, type I, alpha 1 (COL1A1)
Exon Expression
15
13.75
12.5
11.25
10
17
Disease
CL
DD
3762200

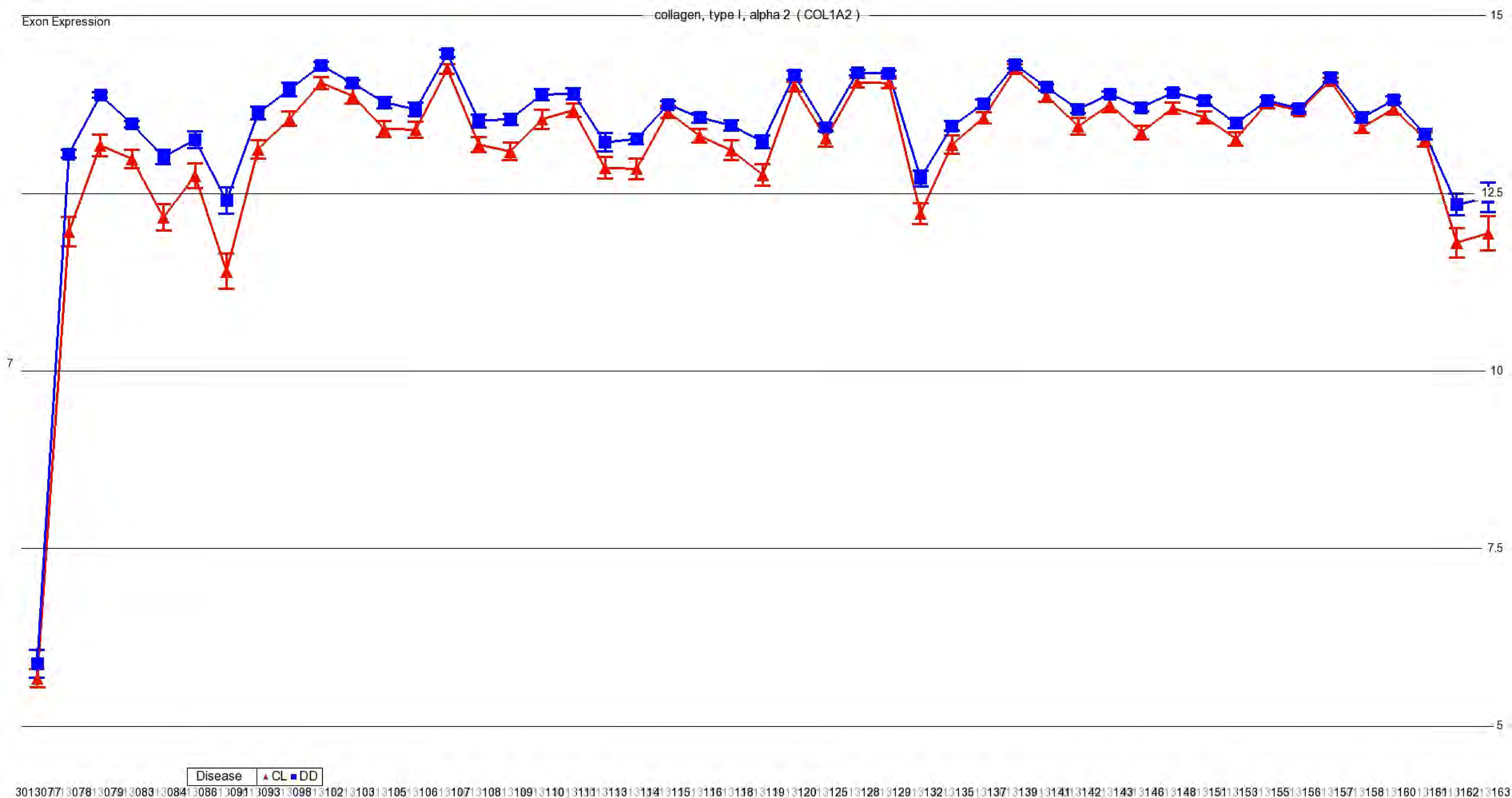

collagen, type I, alpha 2 (COL1A2)
Exon Expression
15
12.5
10
7.5
5
7
Disease
CL
DD

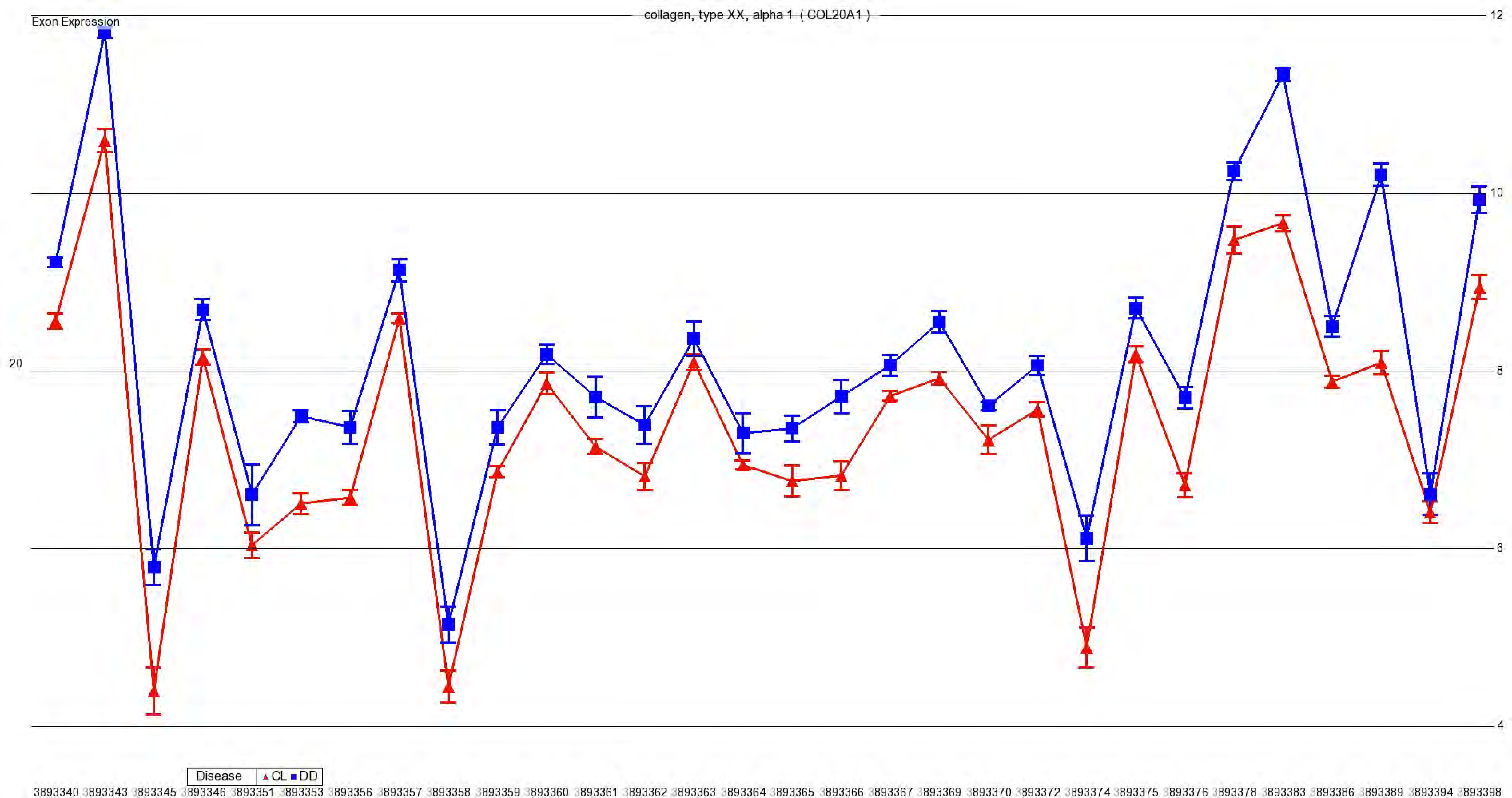

collagen, type XX, alpha 1 (COL20A1)
Exon Expression
20
12
10
8
6
4
Disease
CL
DD
3893340
3893343
3893345
3893346
3893351
3893353
3893356
3893357
3893358
3893359
3893360
3893361
3893362
3893363
3893364
3893365
3893366
3893367
3893369
3893370
3893372
3893374
3893375
3893376
3893378
3893383
3893386
3893389
3893394
3893398

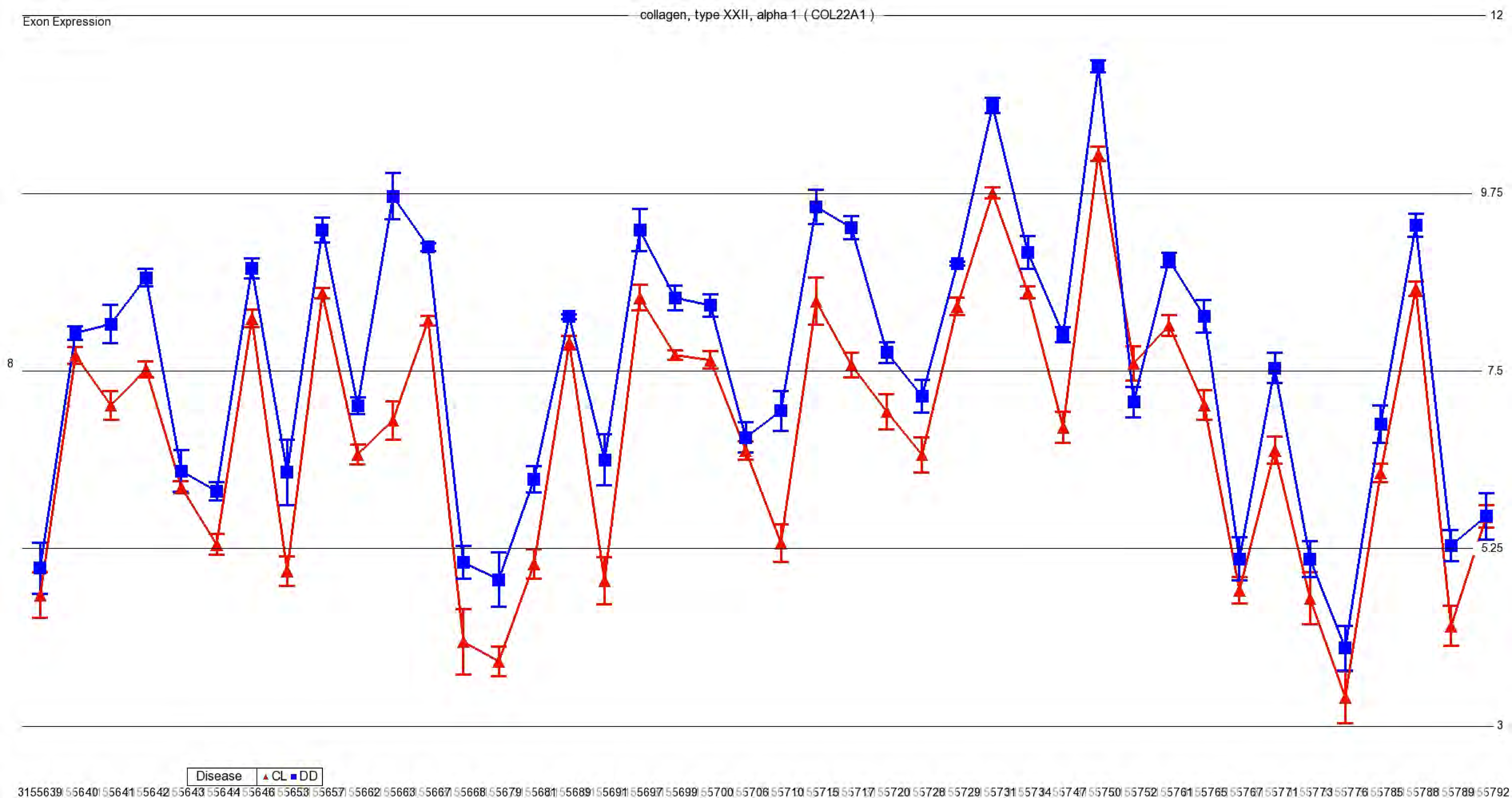

collagen, type XXII, alpha 1 (COL22A1)
Exon Expression
12
9.75
8
7.5
5.25
3
Disease
CL
DD
3155639 55640 55641 55642 55643 55644 55646 55653 55657 55662 55663 55667 55668 55679 55681 55689 55691 55697 55699 55700 55706 55710 55715 55717 55720 55728 55729 55731 55734 55747 55750 55752 55761 55765 55767 55771 55773 55776 55785 55788 55789 55792

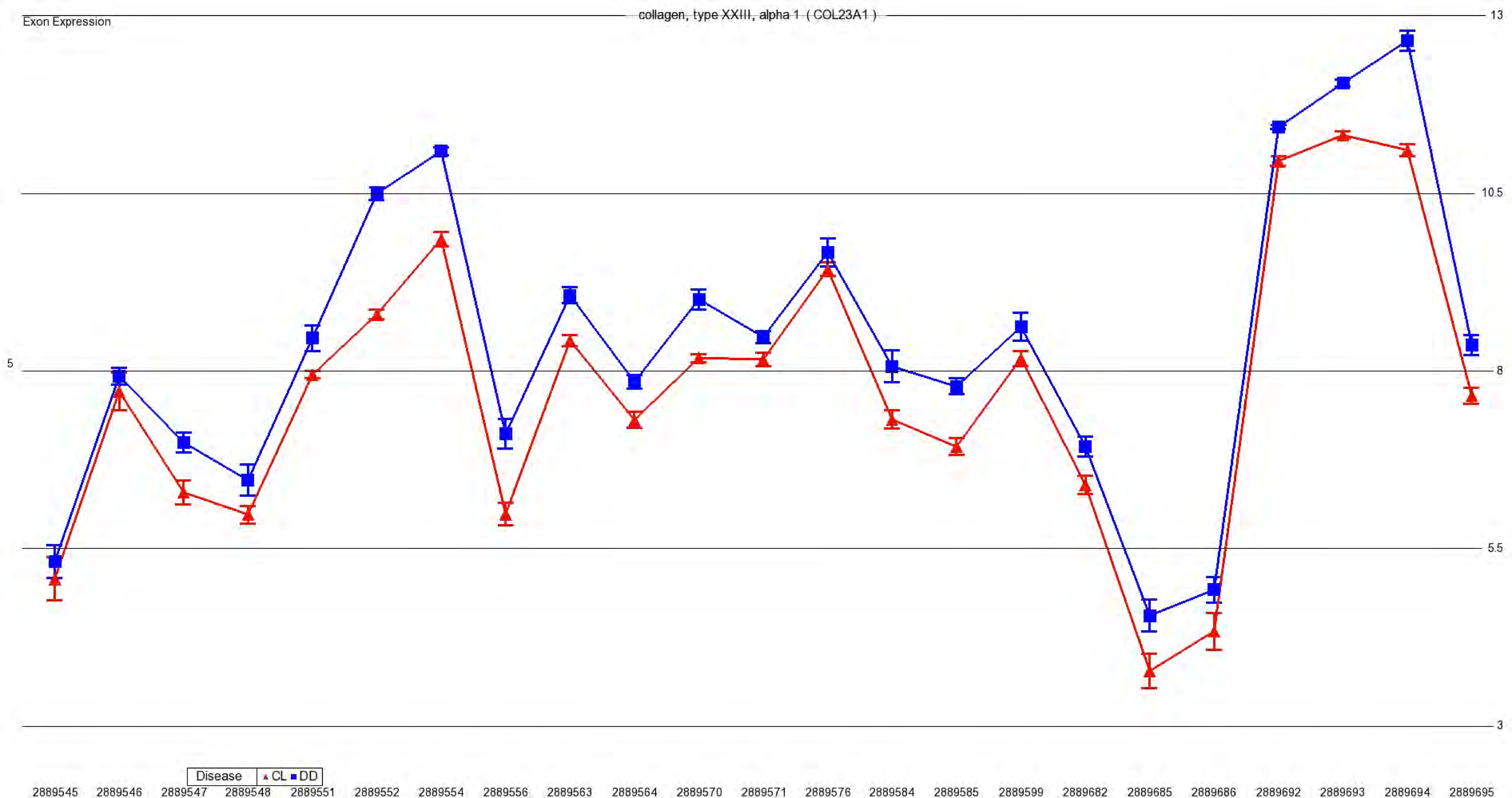

collagen, type XXIII, alpha 1 (COL23A1)
Exon Expression
13
10.5
8
5.5
3
5
Disease
CL
DD
2889545
2889546
2889547
2889548
2889551
2889552
2889554
2889556
2889563
2889564
2889570
2889571
2889576
2889584
2889585
2889599
2889682
2889685
2889686
2889692
2889693
2889694
2889695

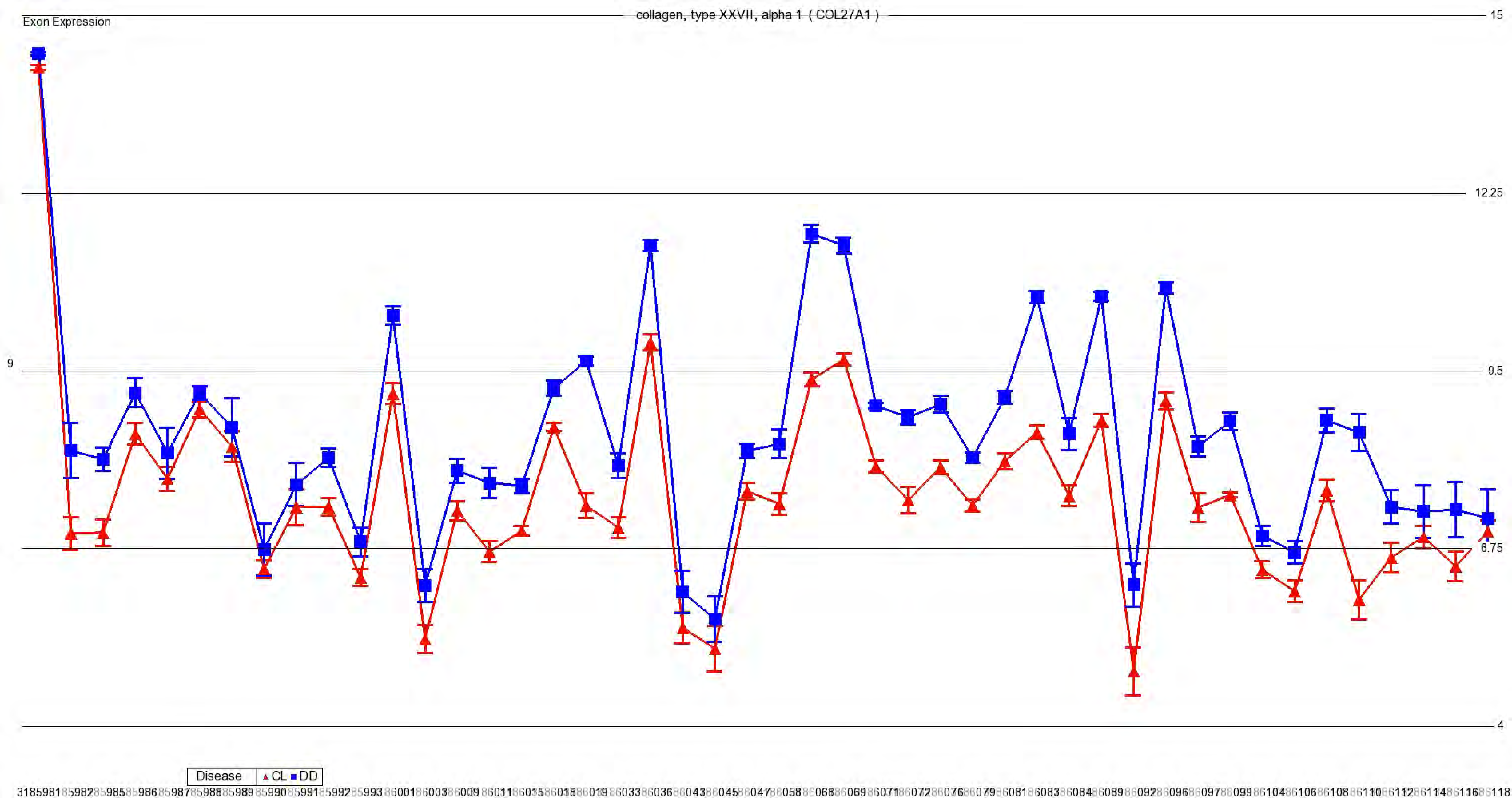
collagen, type XXVII, alpha 1 (COL27A1)
Exon Expression
15
12.25
9.5
9
6.75
4
Disease
CL
DD

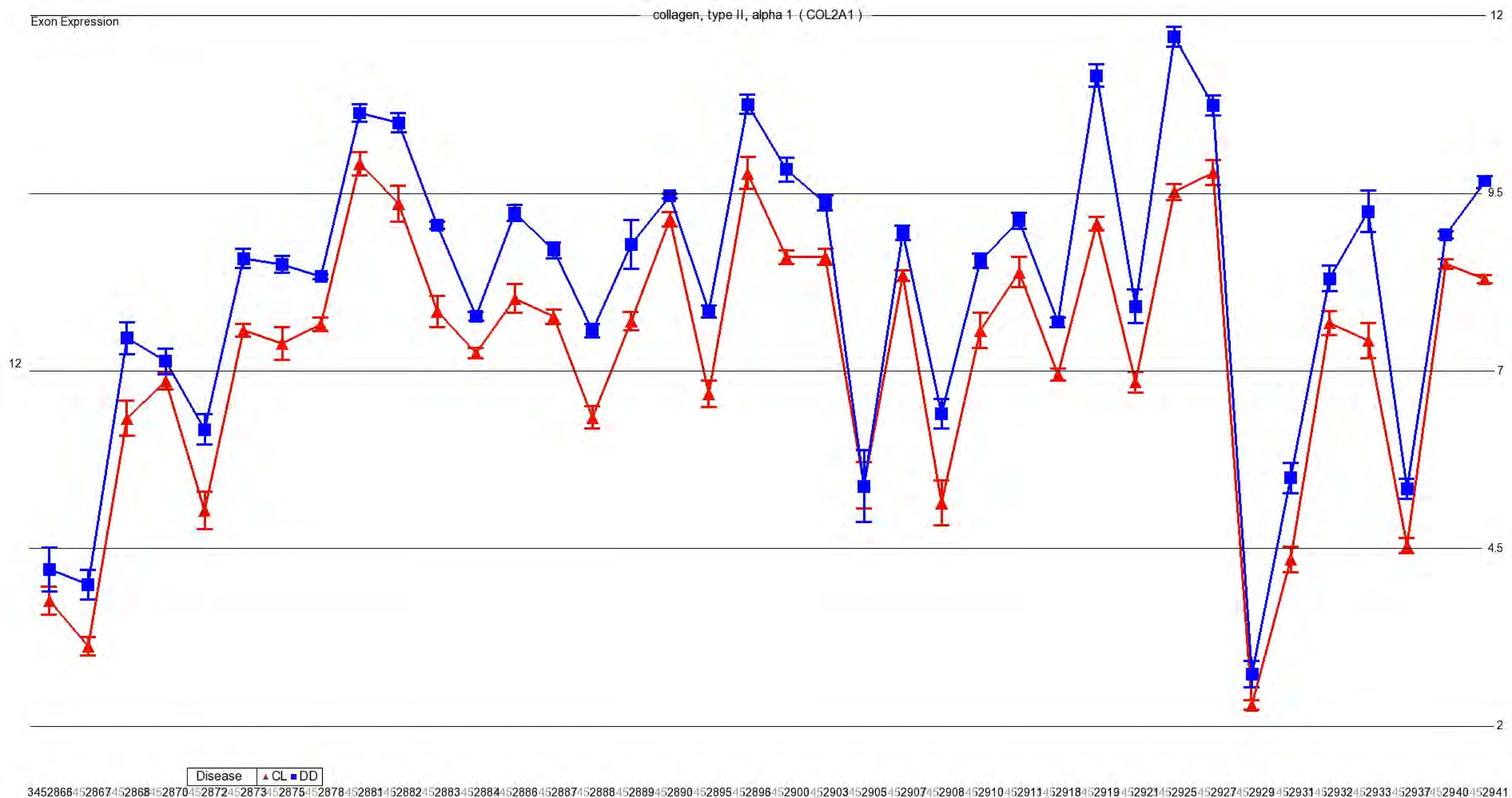

collagen, type II, alpha 1 (COL2A1)
Exon Expression
12
9.5
7
4.5
2
12
Disease
CL
DD
3452866452867452868452870452872452873452875452878452881452882452883452884452886452887452888452889452890452895452896452900452903452905452907452908452910452911452918452919452921452925452927452929452931452932452933452937452940452941

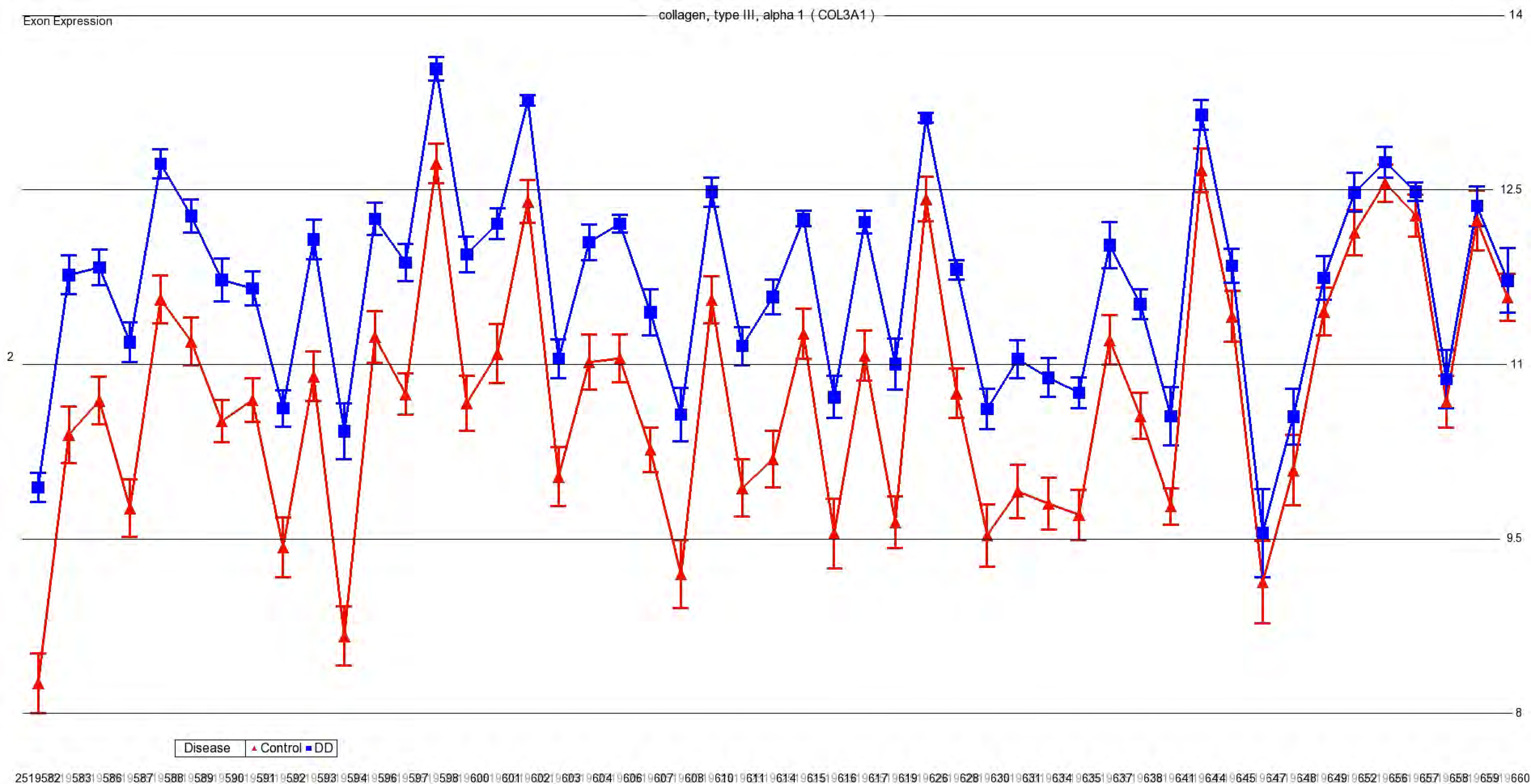

collagen, type III, alpha 1 (COL3A1)
Exon Expression
14
12.5
11
9.5
8
2
Disease
Control
DD
2519582 19583 19586 19587 19588 19589 19590 19591 19592 19593 19594 19596 19597 19598 19600 19601 19602 19603 19604 19606 19607 19608 19610 19611 19614 19615 19616 19617 19619 19626 19628 19630 19631 19634 19635 19637 19638 19641 19644 19645 19647 19648 19649 19652 19656 19657 19658 19659 19660

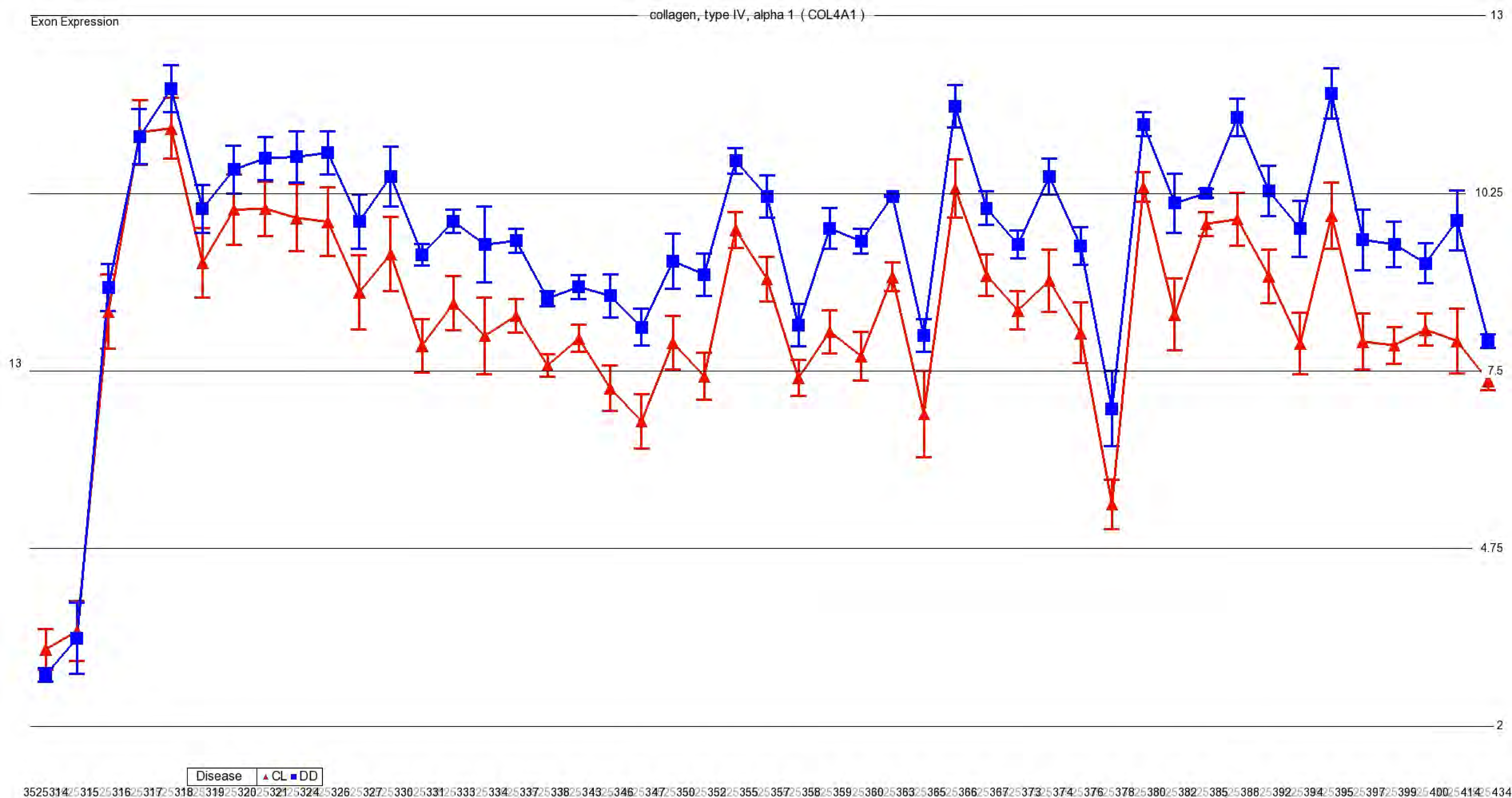
collagen, type IV, alpha 1 (COL4A1)
Exon Expression
13
10.25
7.5
4.75
2
13
Disease
CL
DD

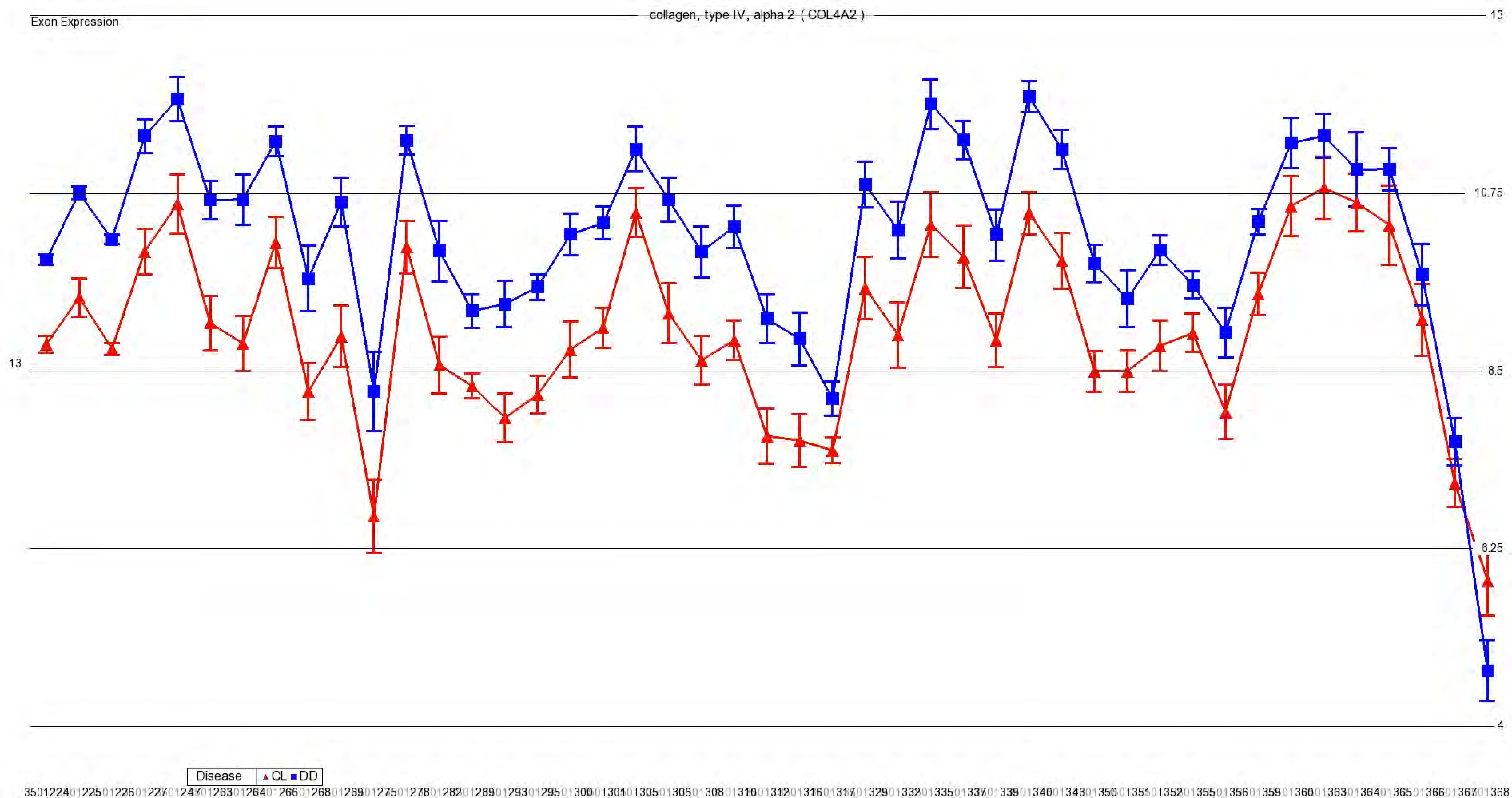

collagen, type IV, alpha 2 (COL4A2)
Exon Expression
13
10.75
8.5
6.25
4
13
Disease
CL
DD
3501224 01225 01226 01227 01247 01263 01264 01266 01268 01269 01275 01278 01282 01289 01293 01295 01300 01301 01305 01306 01308 01310 01312 01316 01317 01329 01332 01335 01337 01339 01340 01343 01350 01351 01352 01355 01356 01359 01360 01363 01364 01365 01366 01367 01368

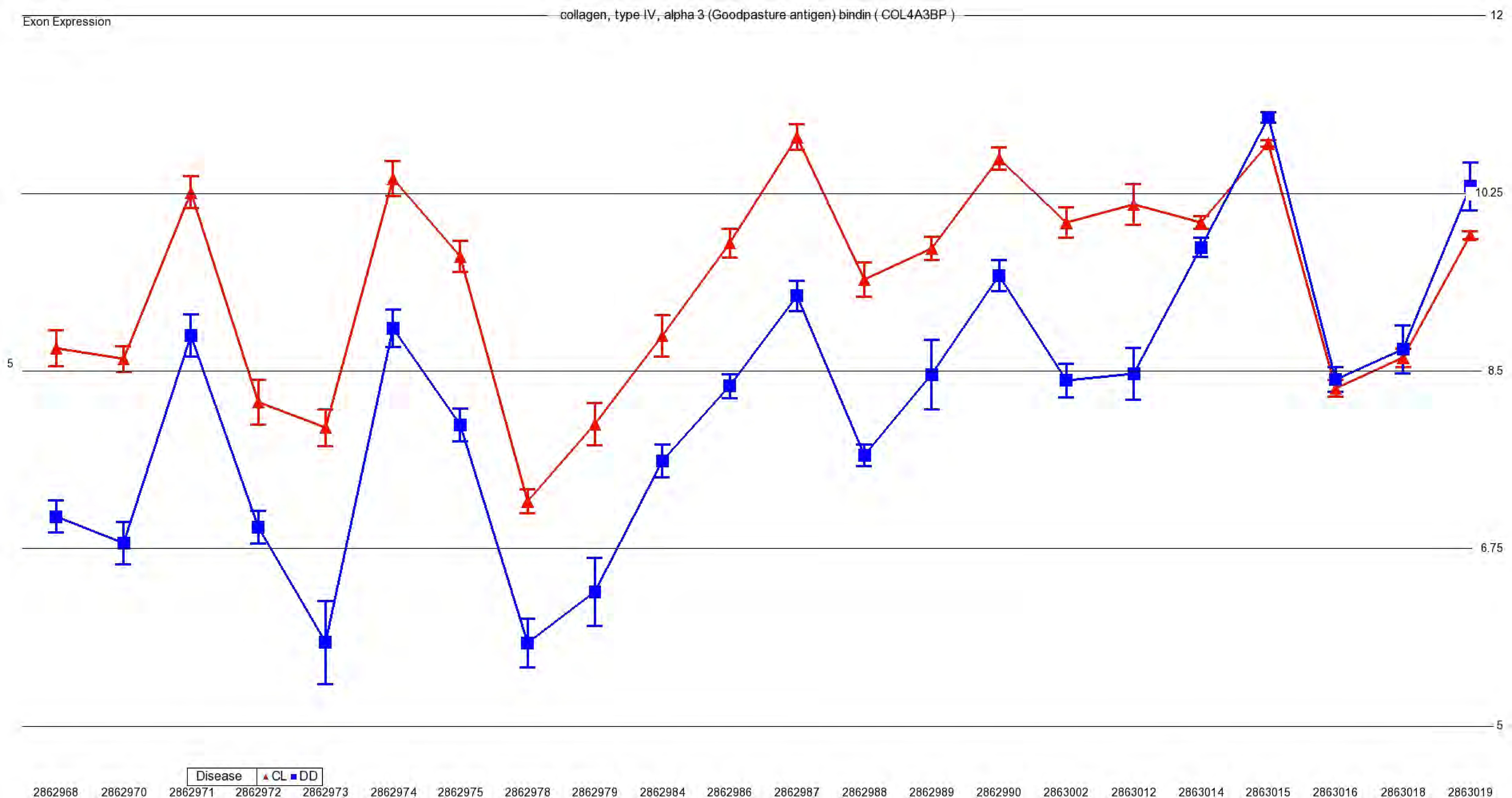
collagen, type IV, alpha 3 (Goodpasture antigen) bindin (COL4A3BP)
Exon Expression
12
10.25
8.5
6.75
5
5
Disease
CL
DD
2862968
2862970
2862971
2862972
2862973
2862974
2862975
2862978
2862979
2862984
2862986
2862987
2862988
2862989
2862990
2863002
2863012
2863014
2863015
2863016
2863018
2863019

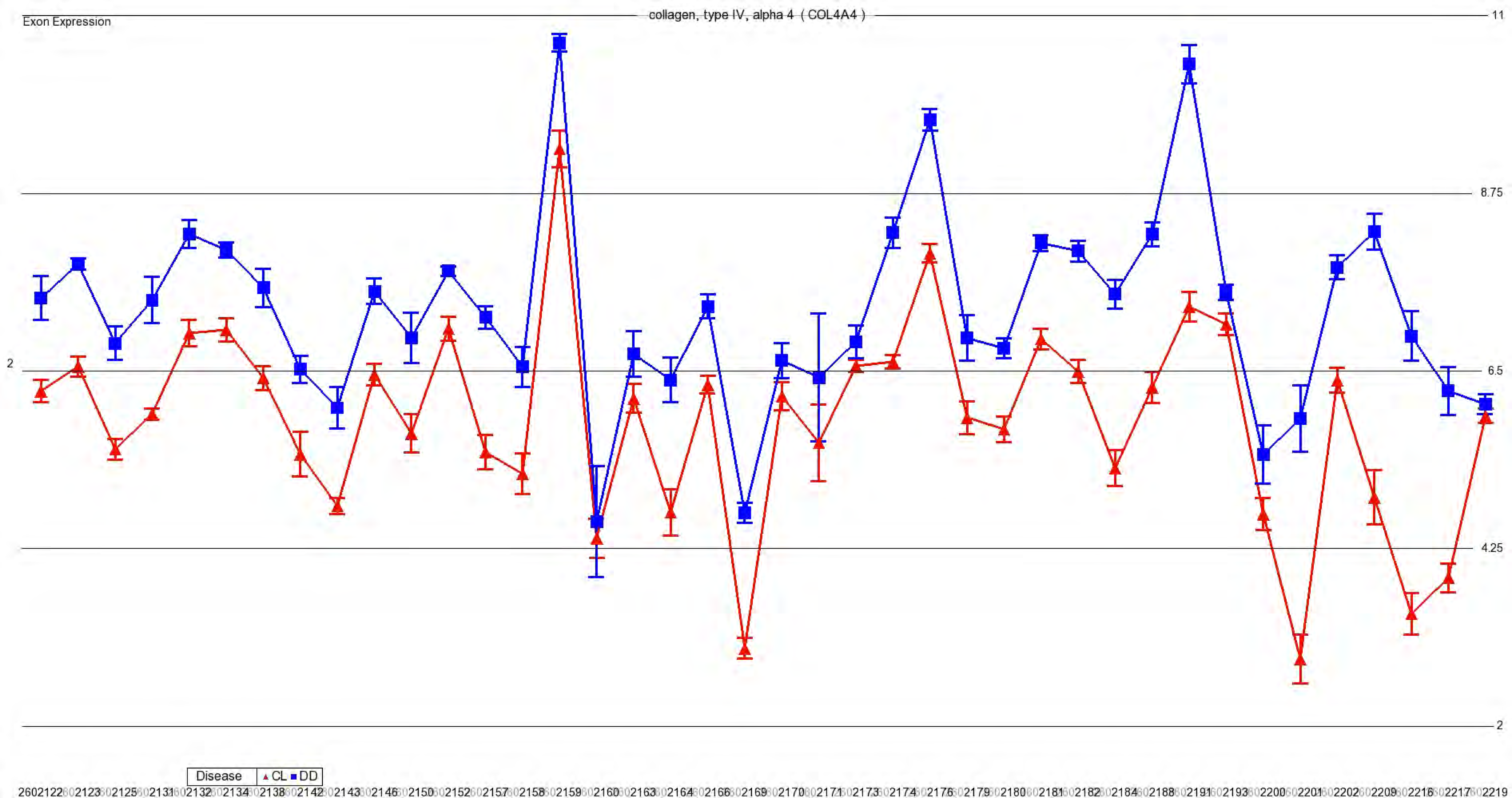

collagen, type IV, alpha 4 (COL4A4)
Exon Expression
11
8.75
6.5
4.25
2
2
Disease
CL
DD
2602122
2123
2125
2131
2132
2134
2138
2142
2143
2146
2150
2152
2157
2158
2159
2160
2163
2164
2166
2169
2170
2171
2173
2174
2176
2179
2180
2181
2182
2184
2188
2191
2193
2200
2201
2202
2209
2216
2217
2219

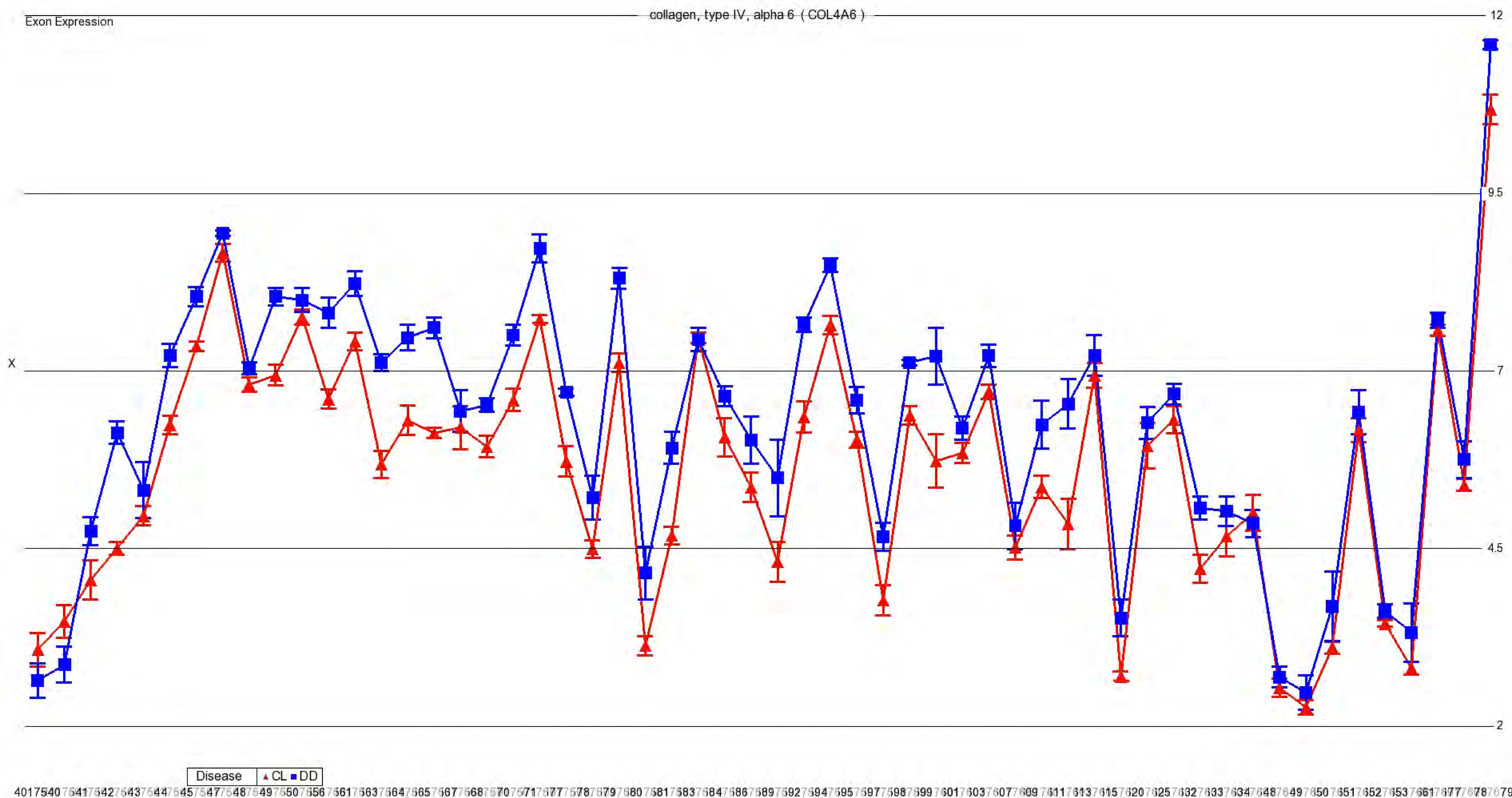
collagen, type IV, alpha 6 (COL4A6)
Exon Expression
X
12
9.5
7
4.5
2
Disease
CL
DD

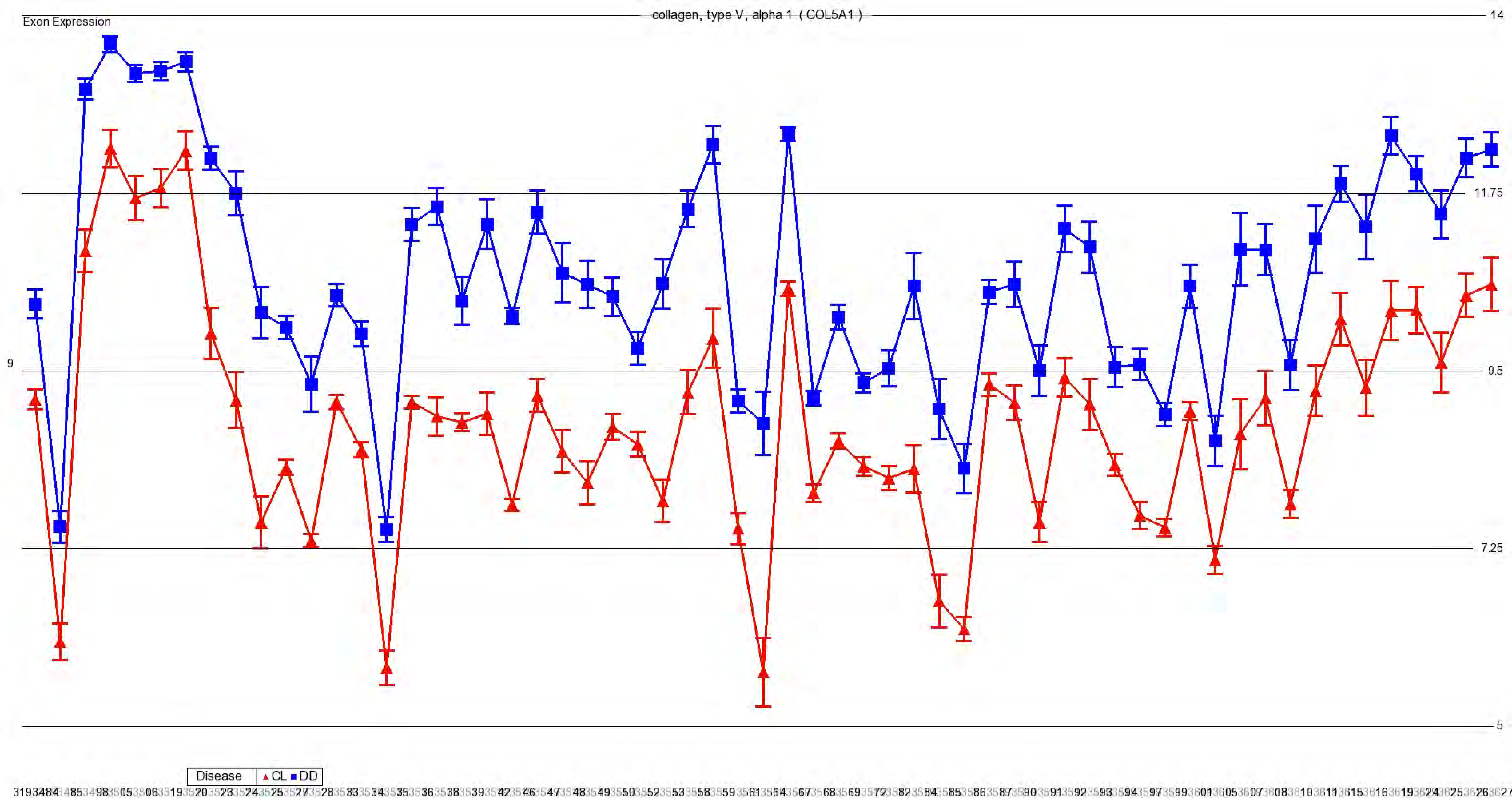

collagen, type V, alpha 1 (COL5A1)
Exon Expression
14
11.75
9
9.5
7.25
5
Disease
CL
DD

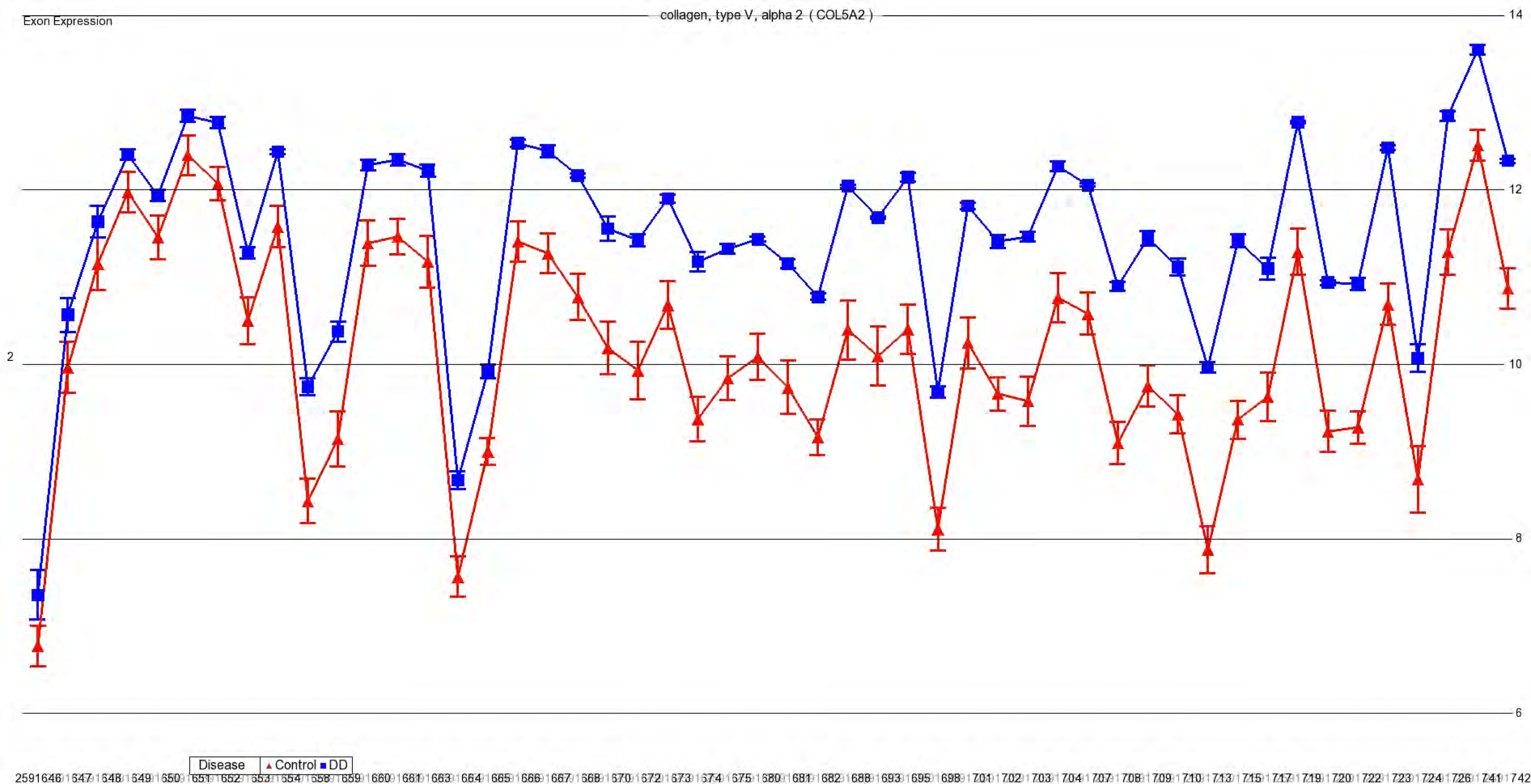

Exon Expression
collagen, type V, alpha 2 (COL5A2)
14
12
10
8
6
2
Disease
Control
DD

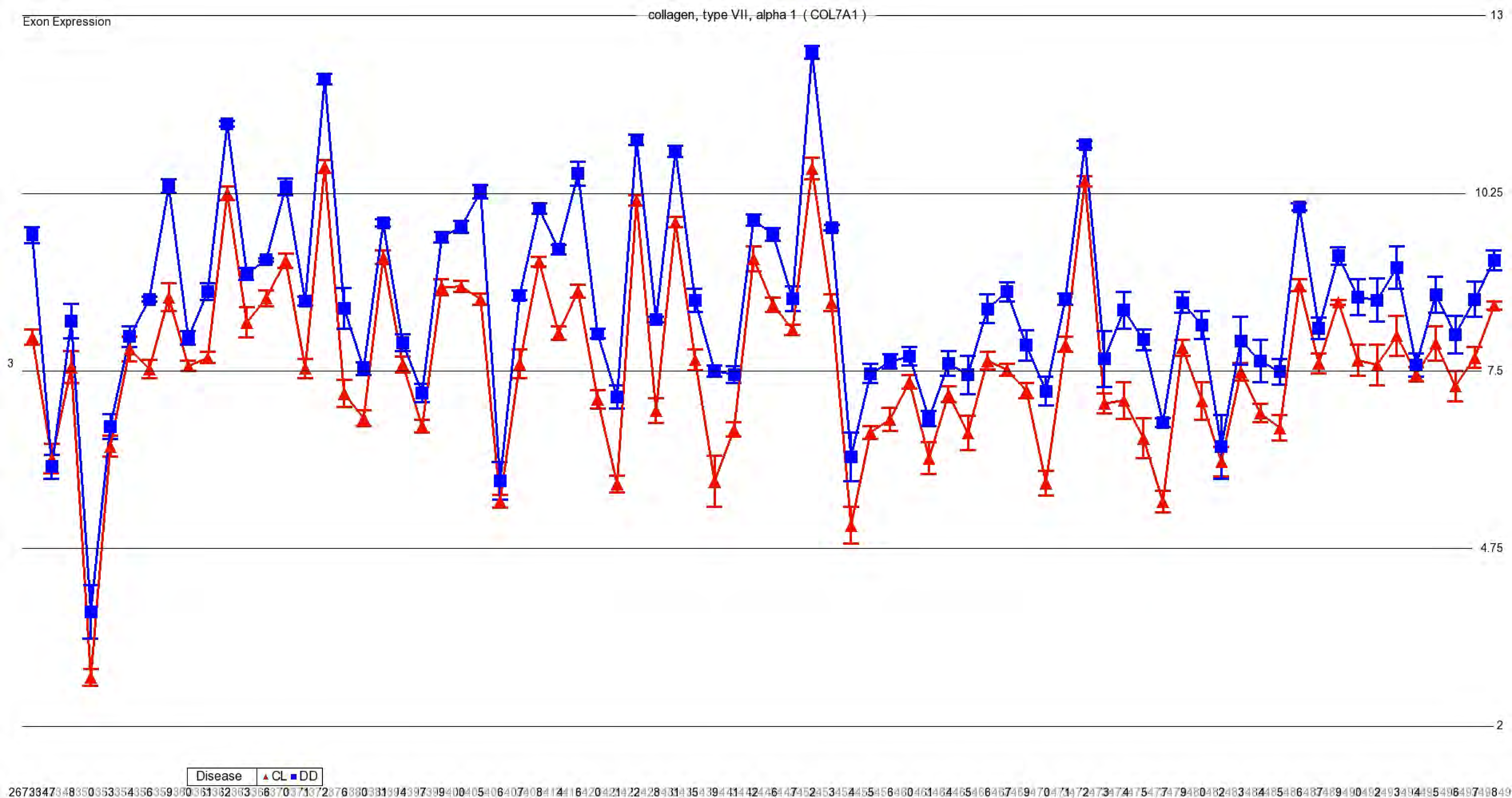

collagen, type VII, alpha 1 (COL7A1)
Exon Expression
13
10.25
7.5
4.75
2
Disease
CL
DD

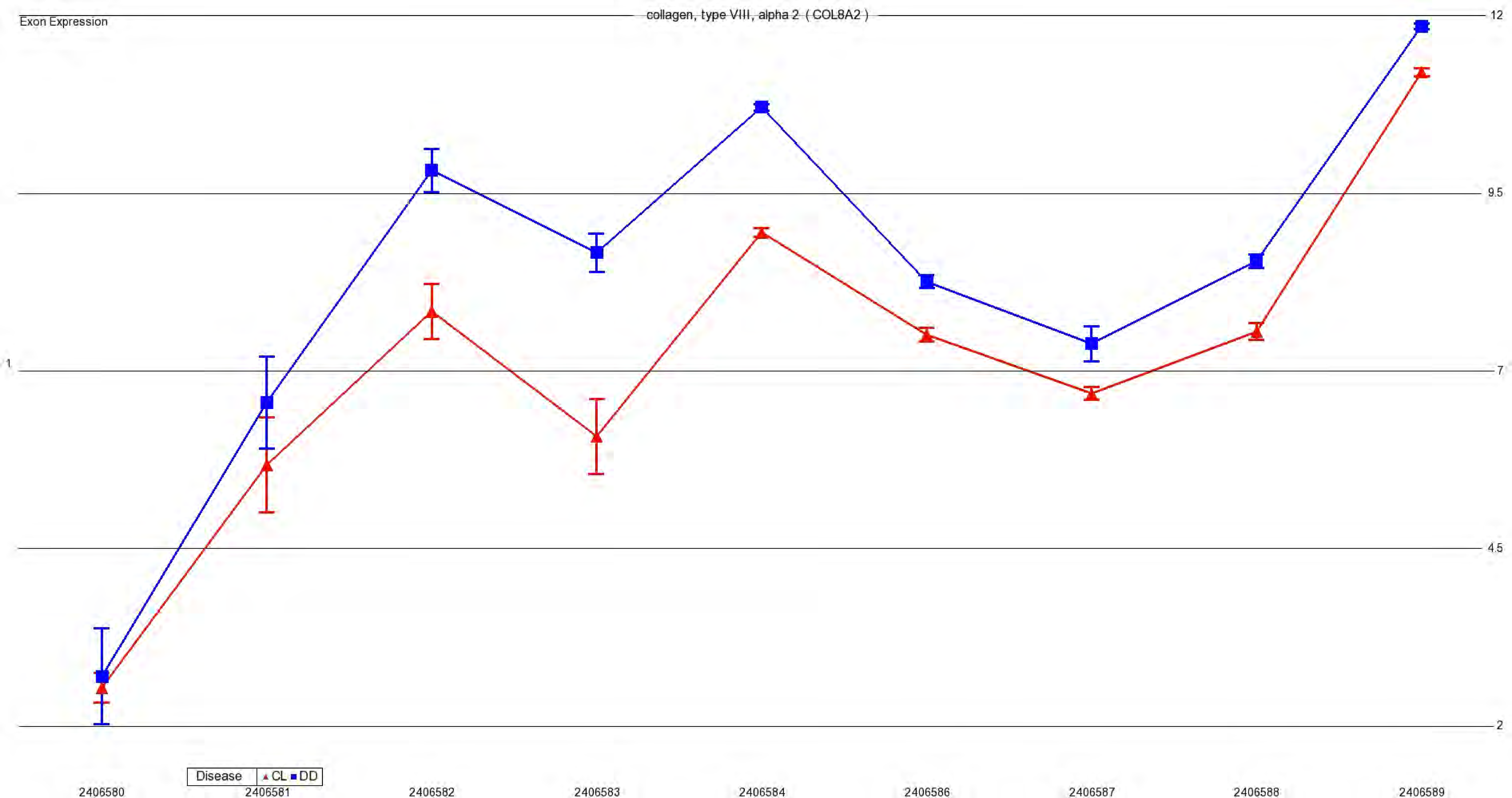

Exon Expression
collagen, type VIII, alpha 2 (COL8A2)
12
9.5
7
4.5
2
1
Disease
CL
DD
2406580
2406581
2406582
2406583
2406584
2406586
2406587
2406588
2406589

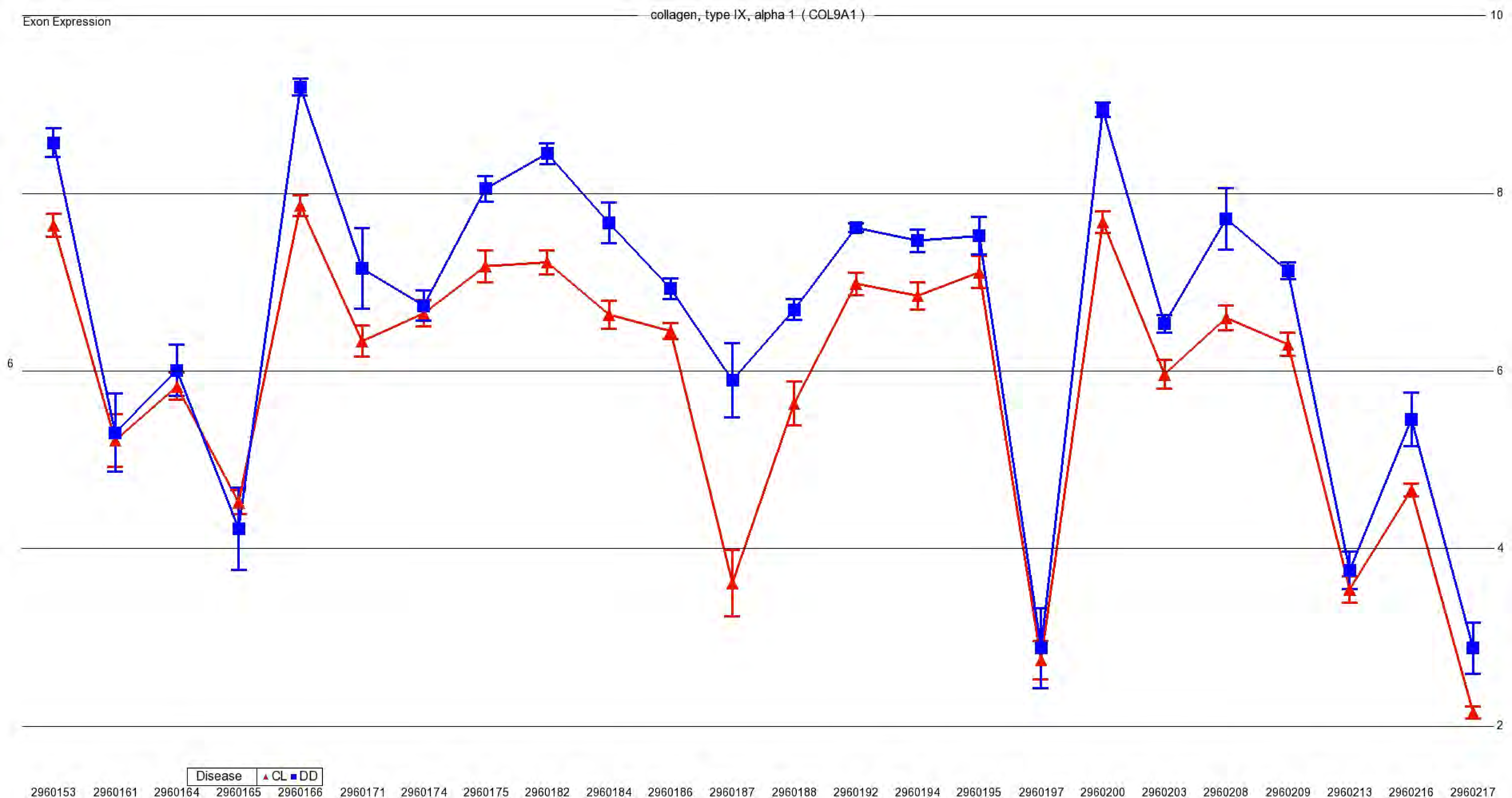

Exon Expression
collagen, type IX, alpha 1 (COL9A1)
10
8
6
6
4
2
Disease
CL
DD
2960153
2960161
2960164
2960165
2960166
2960171
2960174
2960175
2960182
2960184
2960186
2960187
2960188
2960192
2960194
2960195
2960197
2960200
2960203
2960208
2960209
2960213
2960216
2960217

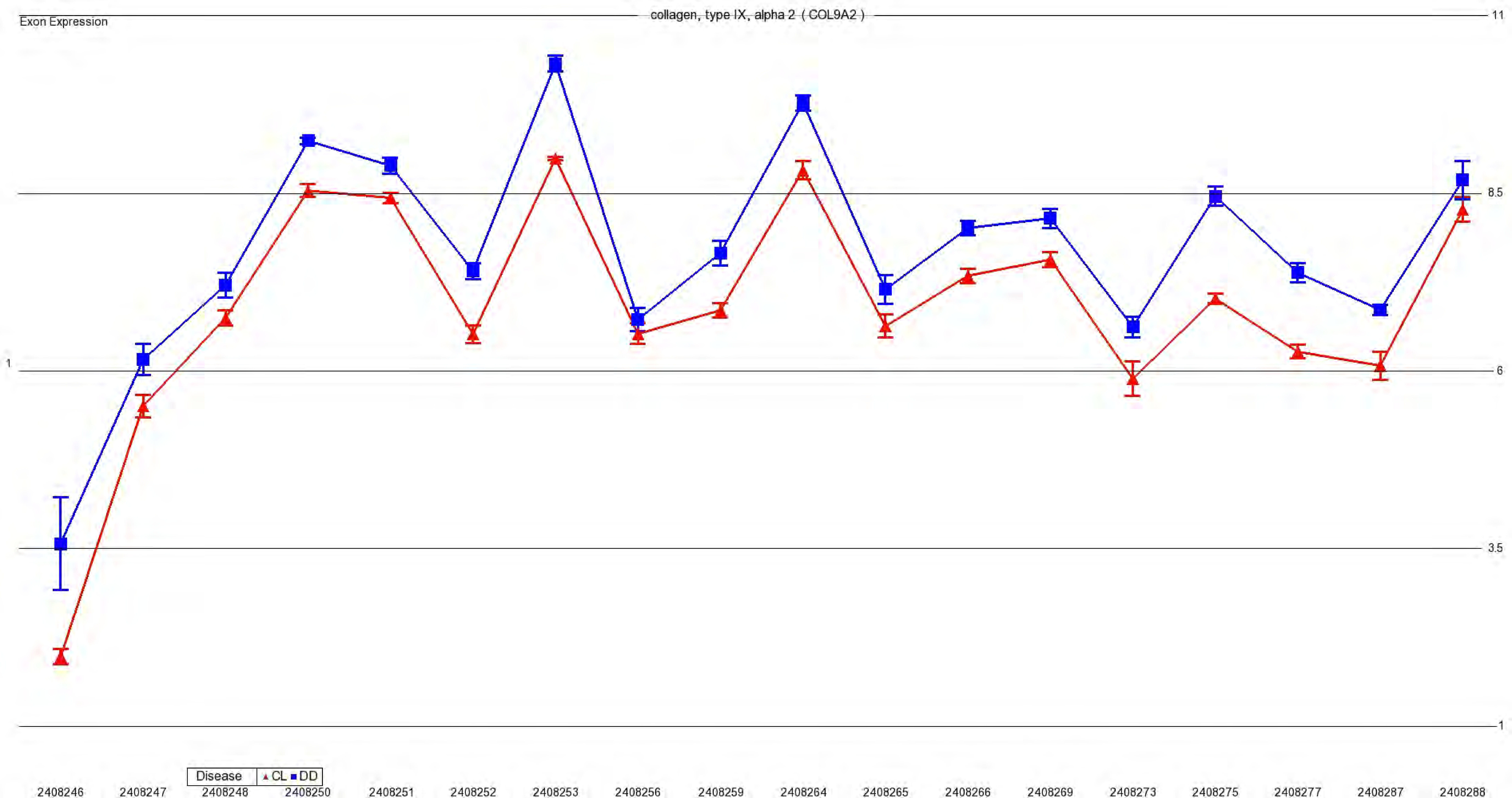

collagen, type IX, alpha 2 (COL9A2)
Exon Expression
11
8.5
6
3.5
1
1
Disease
CL
DD
2408246
2408247
2408248
2408250
2408251
2408252
2408253
2408256
2408259
2408264
2408265
2408266
2408269
2408273
2408275
2408277
2408287
2408288

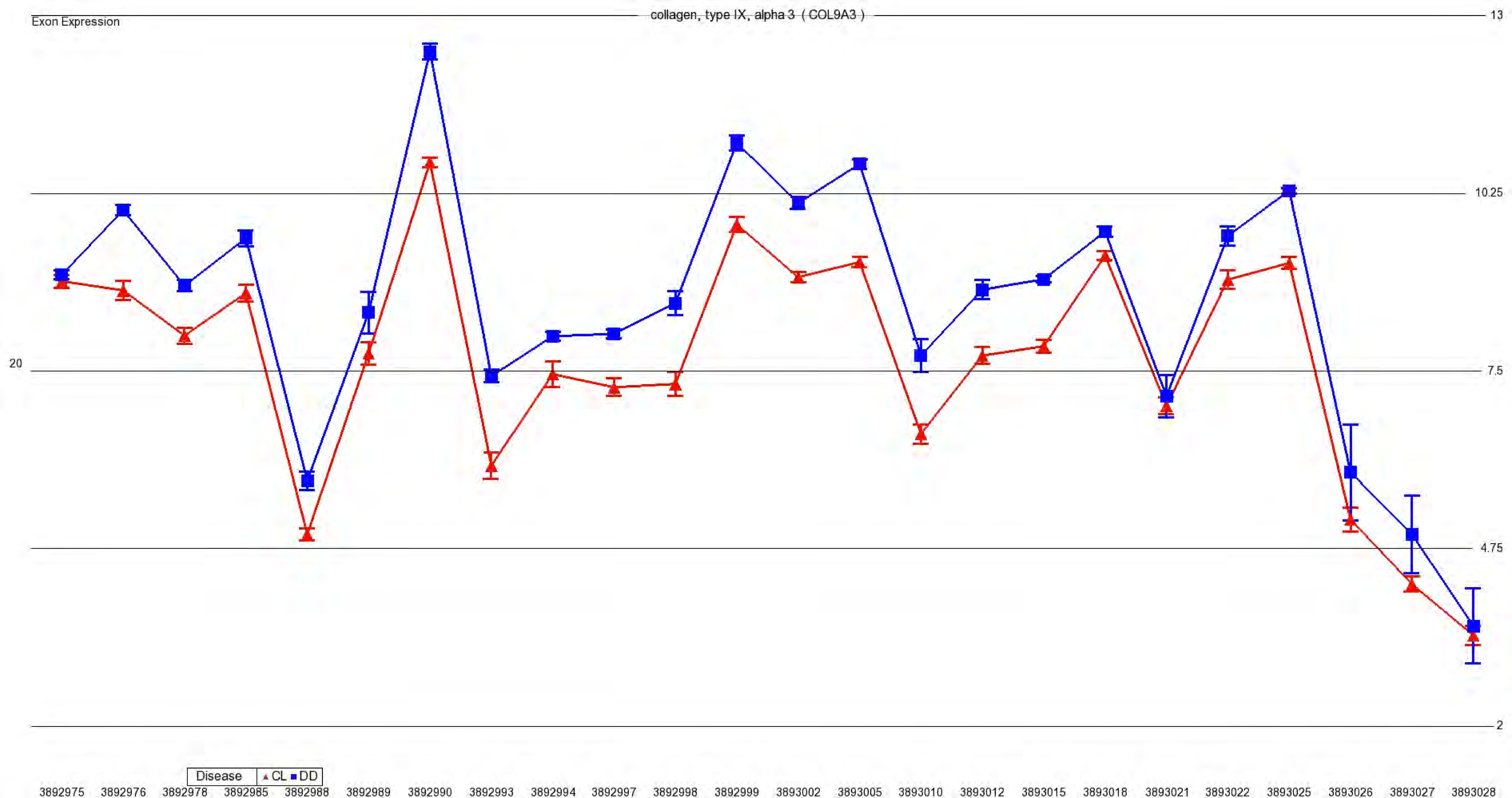

Exon Expression
collagen, type IX, alpha 3 (COL9A3)
13
10.25
7.5
4.75
2
20
Disease
CL
DD
3892975
3892976
3892978
3892985
3892988
3892989
3892990
3892993
3892994
3892997
3892998
3892999
3893002
3893005
3893010
3893012
3893015
3893018
3893021
3893022
3893025
3893026
3893027
3893028

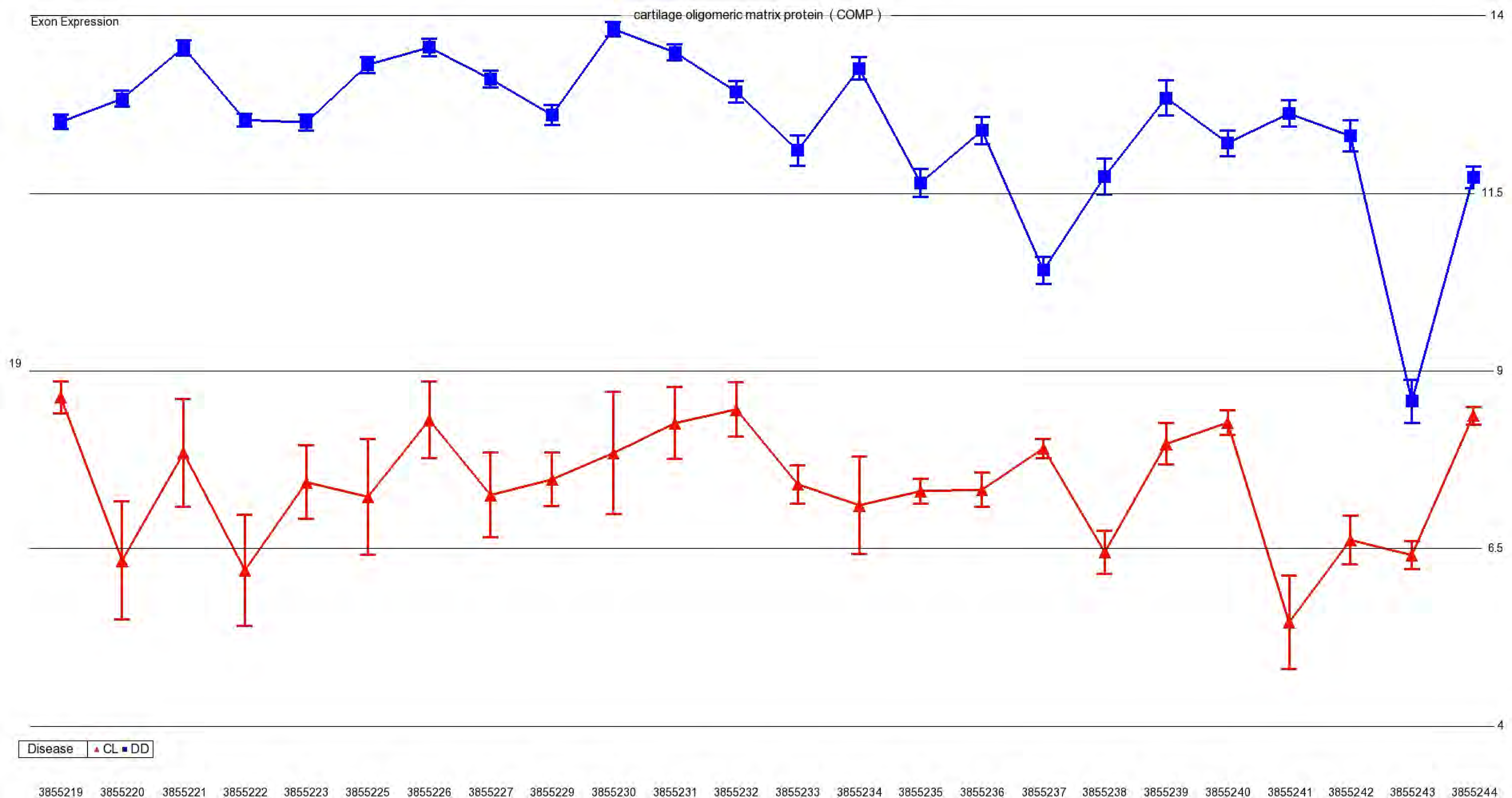
cartilage oligomeric matrix protein (COMP)
Exon Expression
14
11.5
19
9
6.5
4
Disease
CL
DD
3855219
3855220
3855221
3855222
3855223
3855225
3855226
3855227
3855229
3855230
3855231
3855232
3855233
3855234
3855235
3855236
3855237
3855238
3855239
3855240
3855241
3855242
3855243
3855244

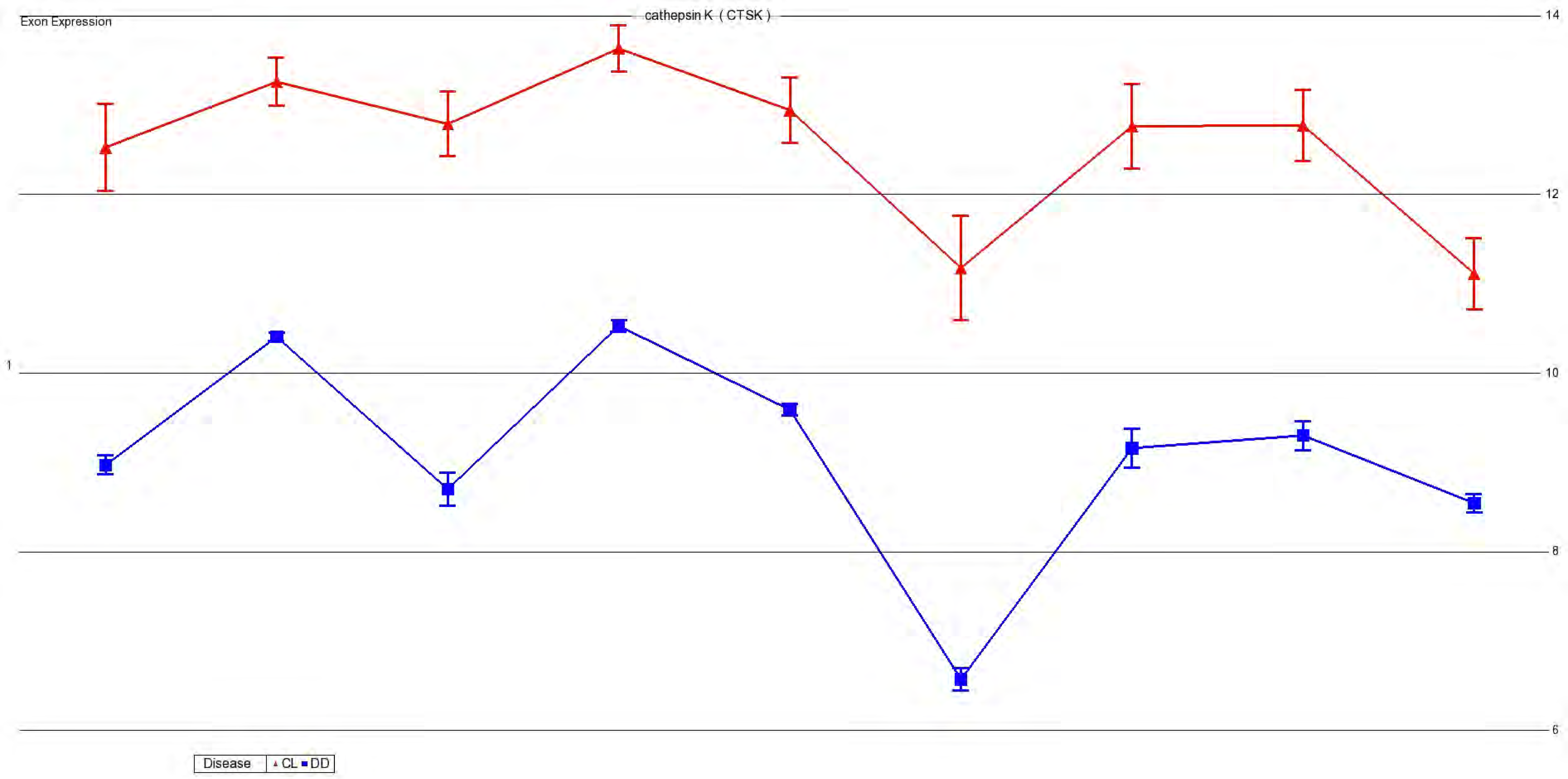

cathepsin K (CTSK)
Exon Expression
14
12
1
10
8
6
Disease
CL
DD

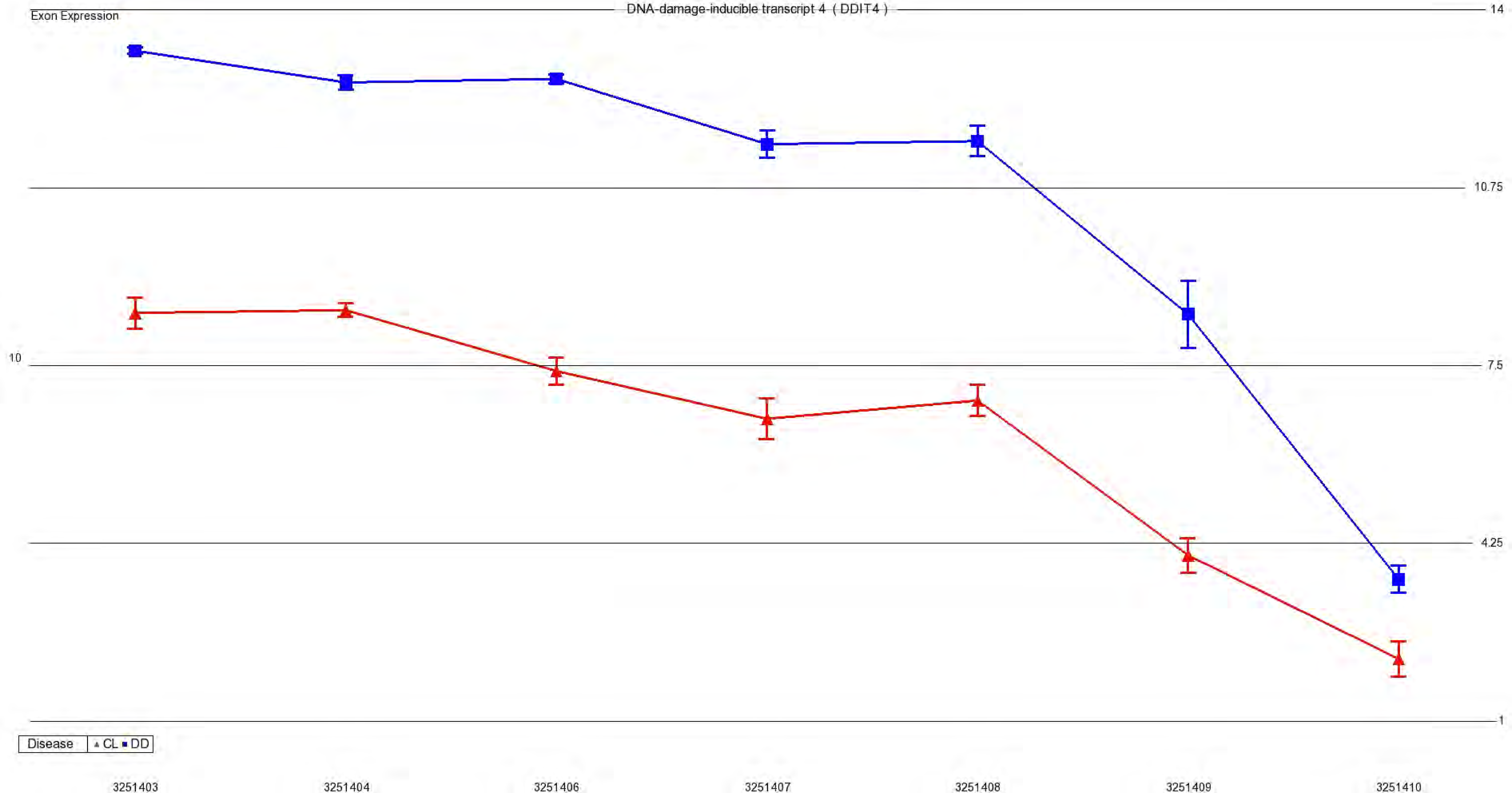

DNA-damage-inducible transcript 4 (DDIT4)
Exon Expression
14
10.75
10
7.5
4.25
1
Disease
CL
DD
3251403
3251404
3251406
3251407
3251408
3251409
3251410

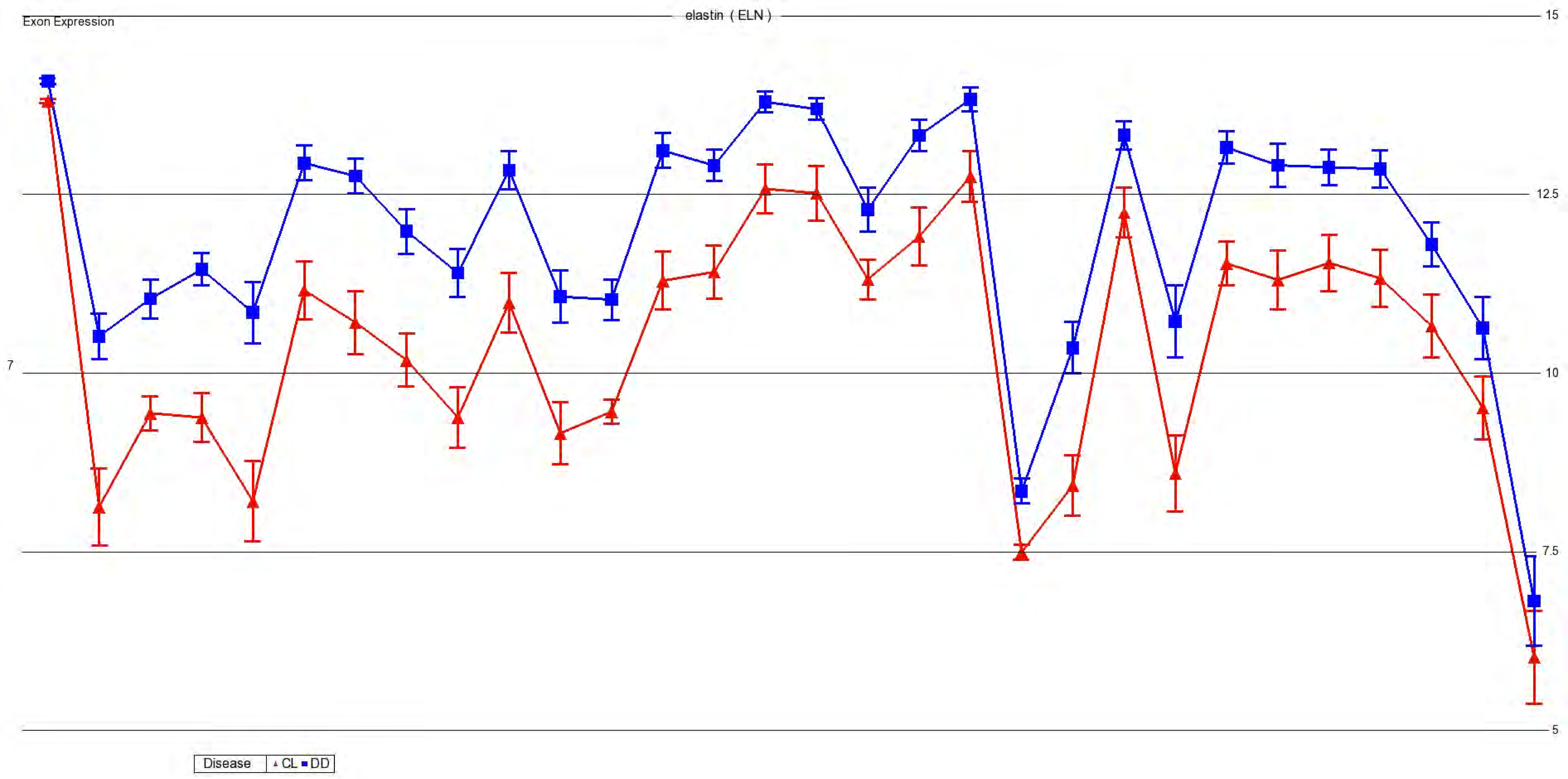

Exon Expression
elastin (ELN)
15
12.5
7
10
7.5
5
Disease
CL
DD

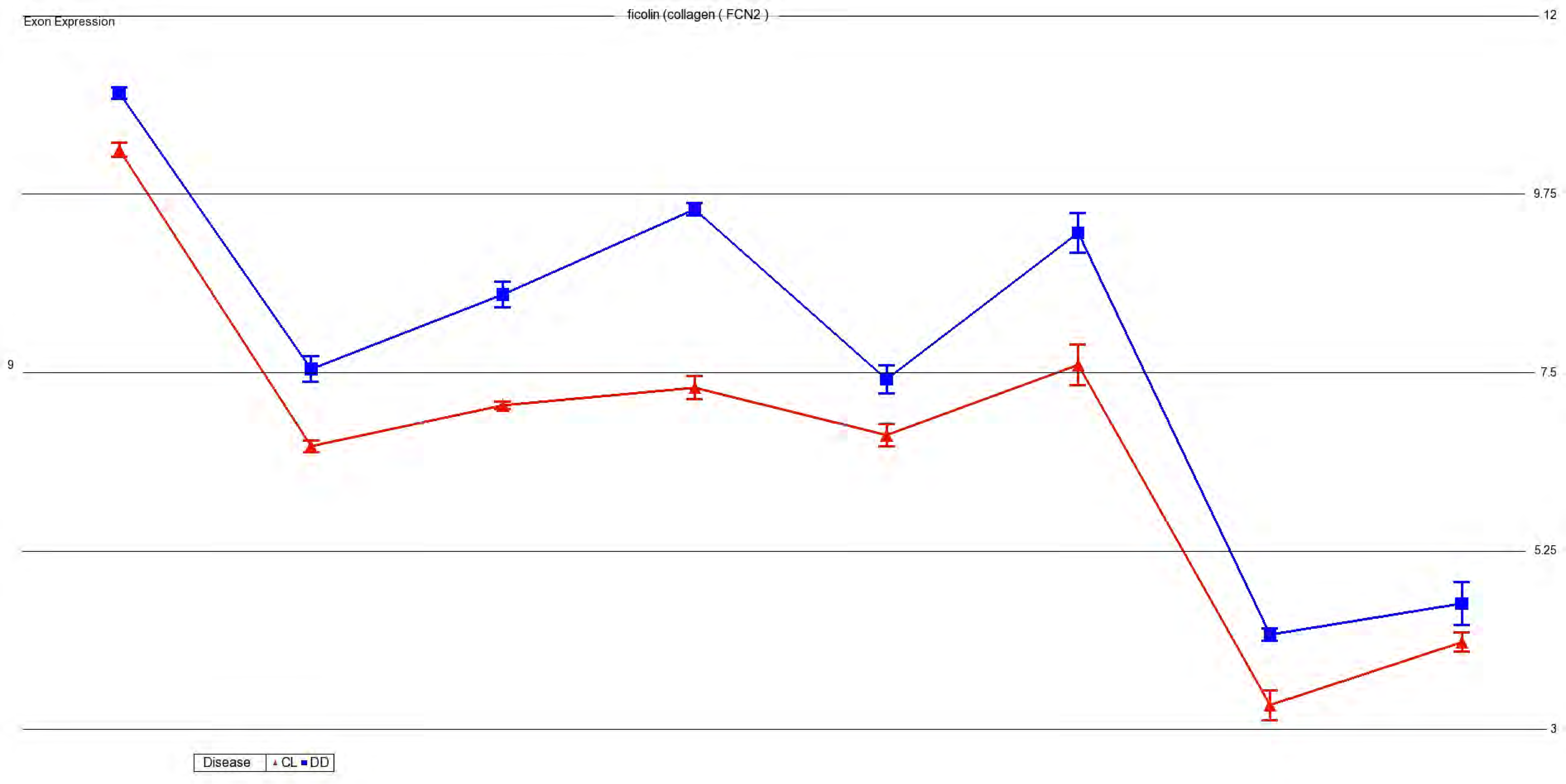

ficolin (collagen (FCN2)
Exon Expression
12
9.75
9
7.5
5.25
3
Disease
CL
DD

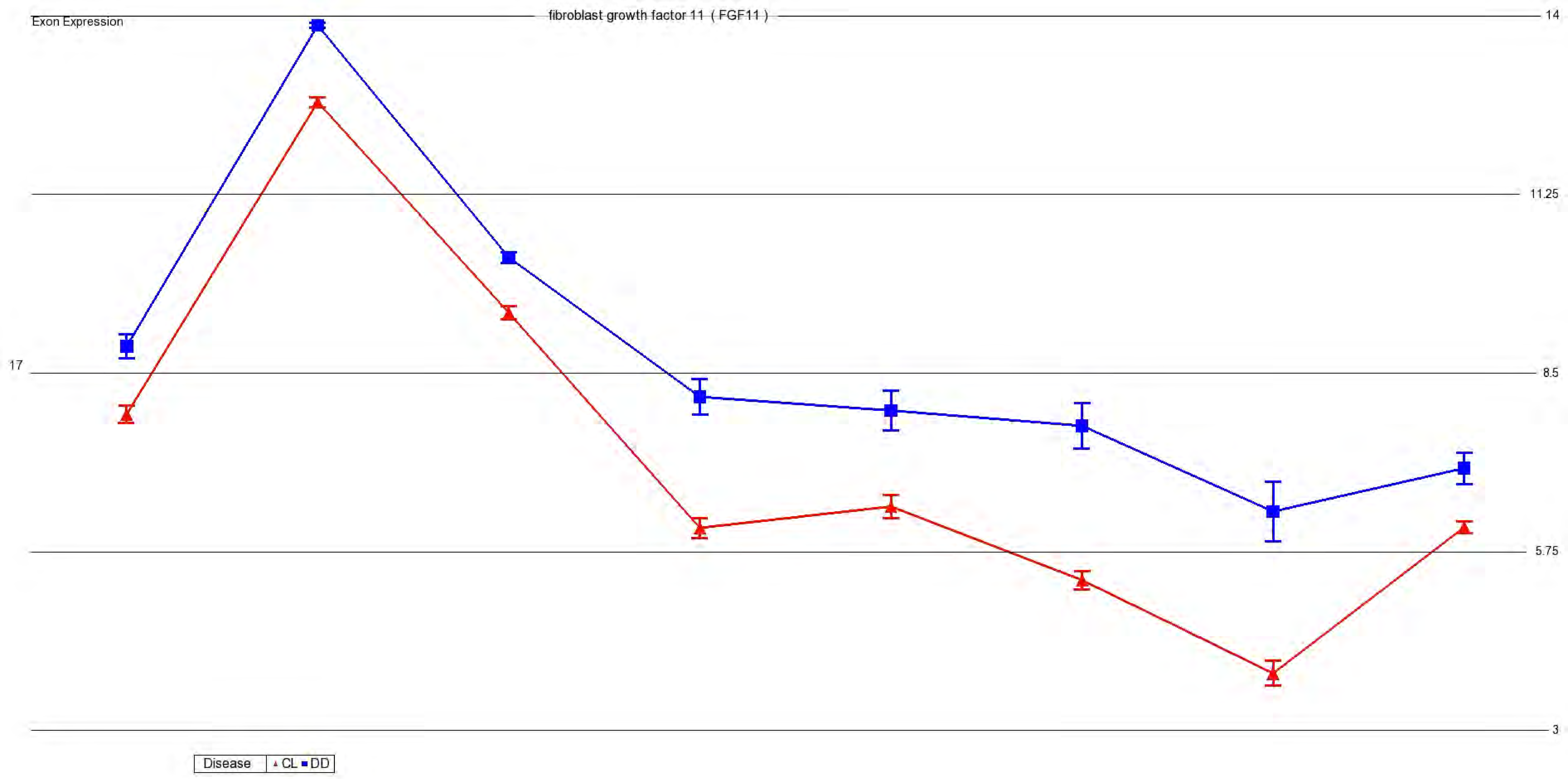
fibroblast growth factor 11 (FGF11)
Exon Expression
14
11.25
17
8.5
5.75
3
Disease
CL
DD

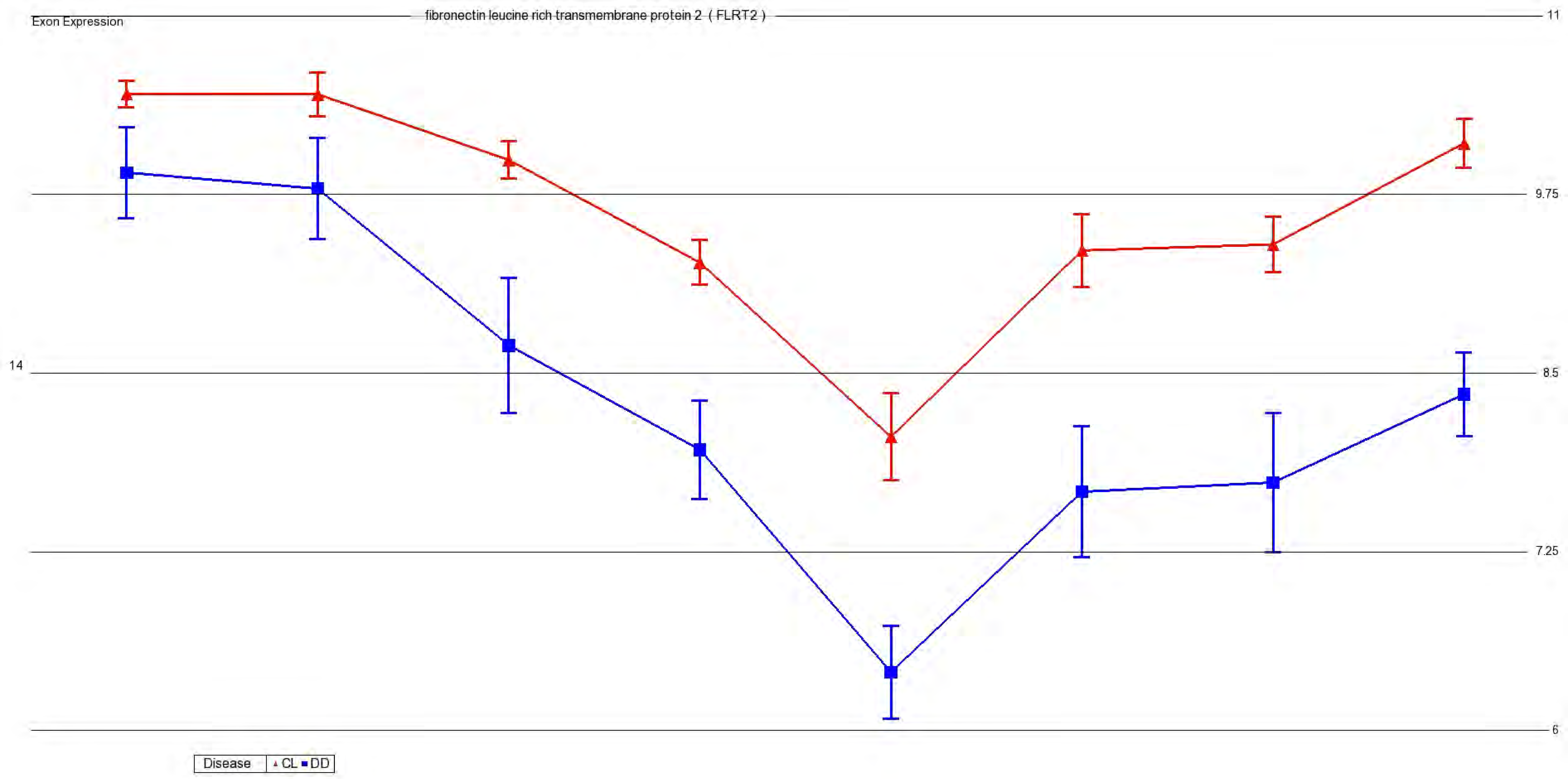

Exon Expression
fibronectin leucine rich transmembrane protein 2 (FLRT2)
11
9.75
14
8.5
7.25
6
Disease
CL
DD

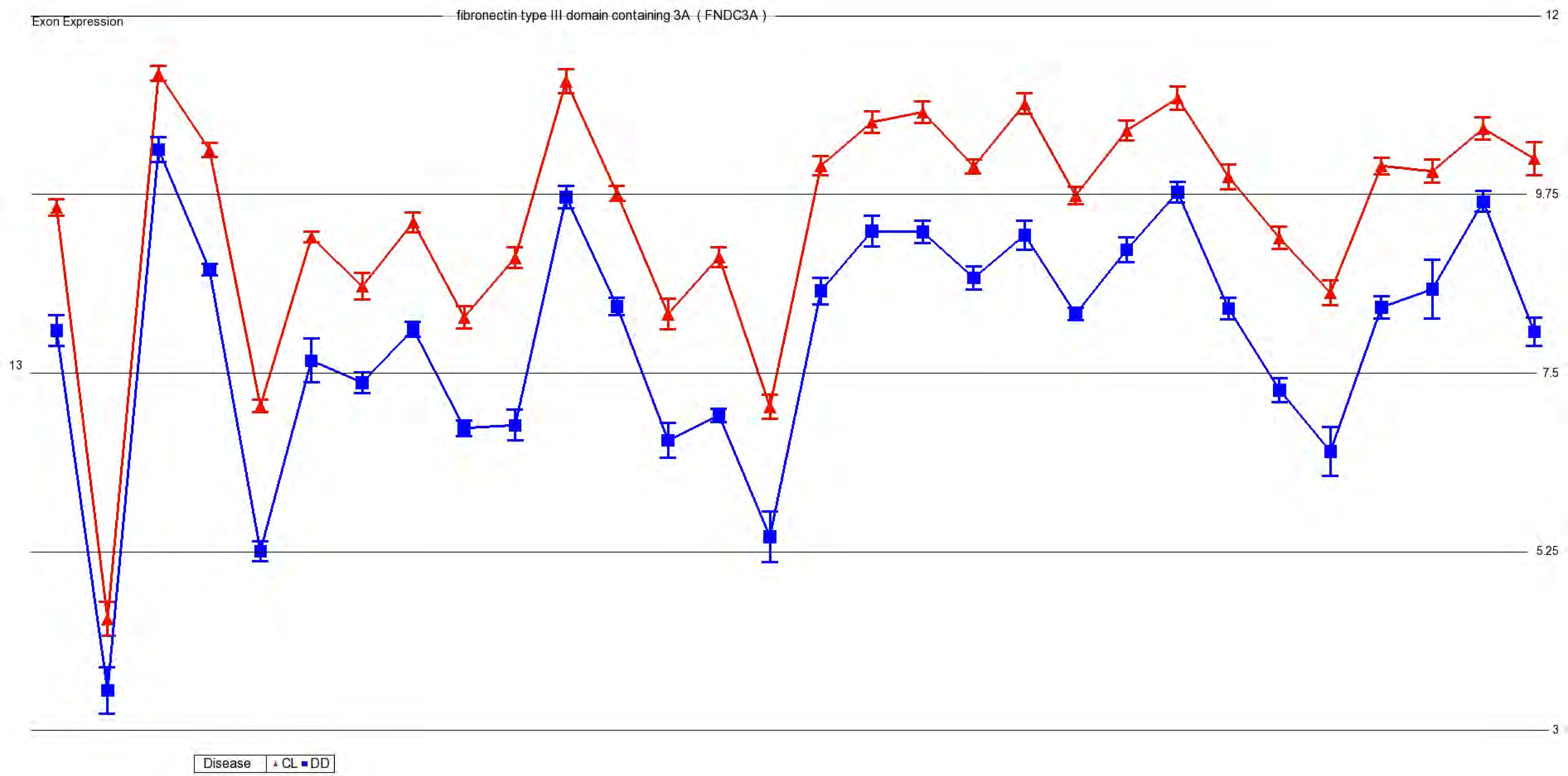

Exon Expression
fibronectin type III domain containing 3A (FNDC3A)
12
9.75
13
7.5
5.25
3
Disease
CL
DD

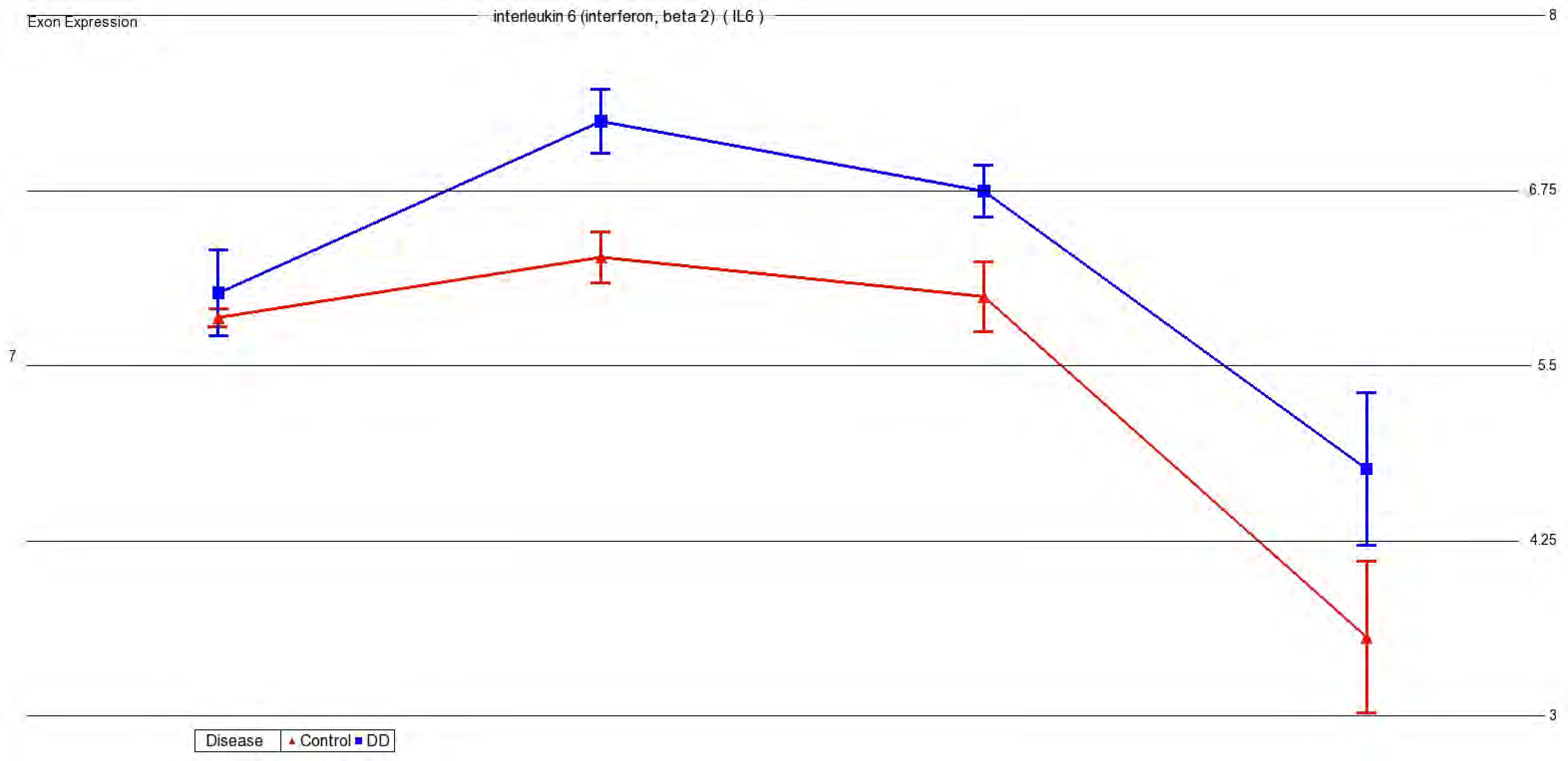
Exon Expression
interleukin 6 (interferon, beta 2) (IL6)
8
6.75
7
5.5
4.25
3
Disease
Control
DD

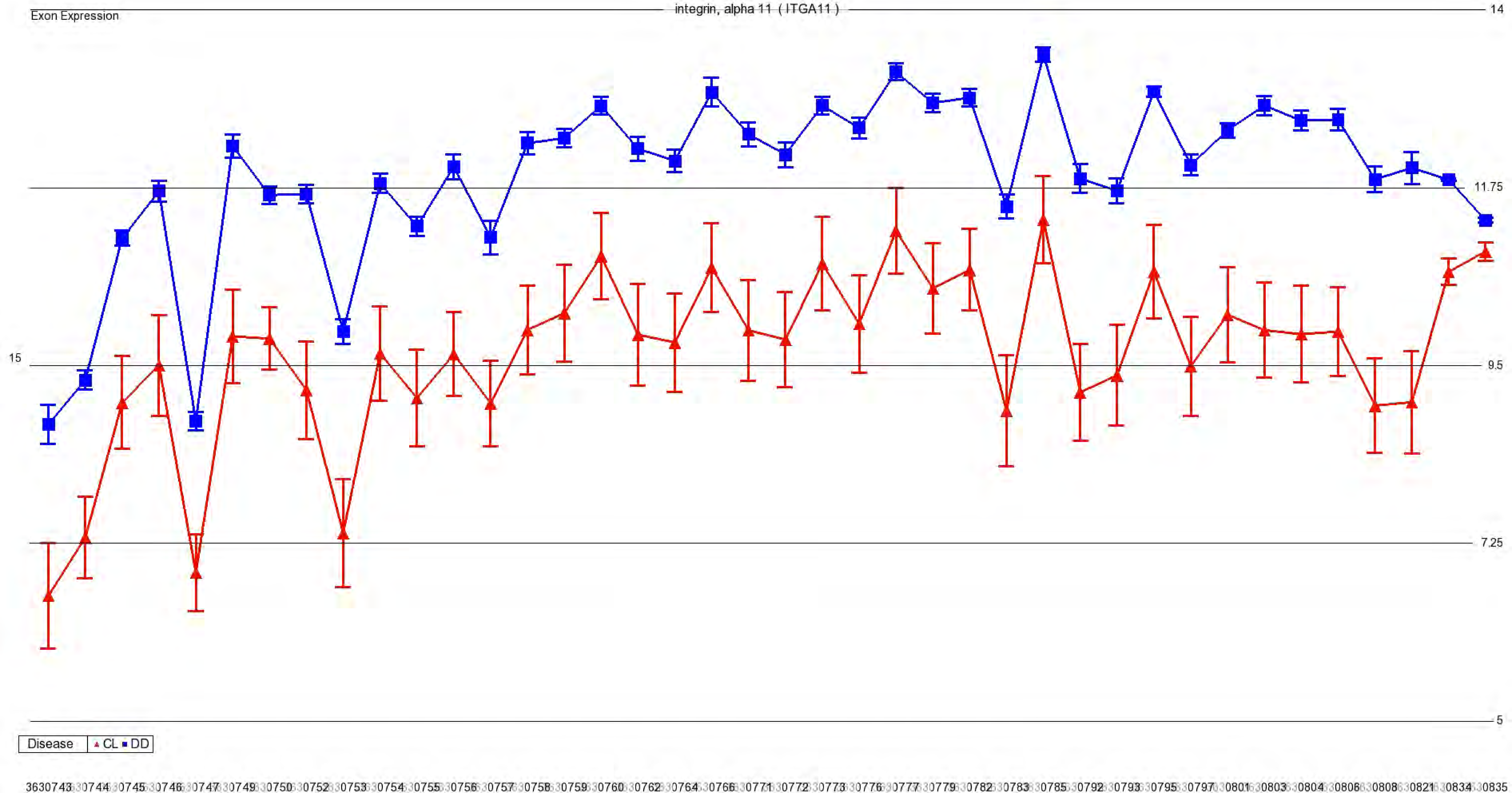

Exon Expression
integrin, alpha 11 (ITGA11)
14
11.75
15
9.5
7.25
5
Disease
CL
DD
3630743

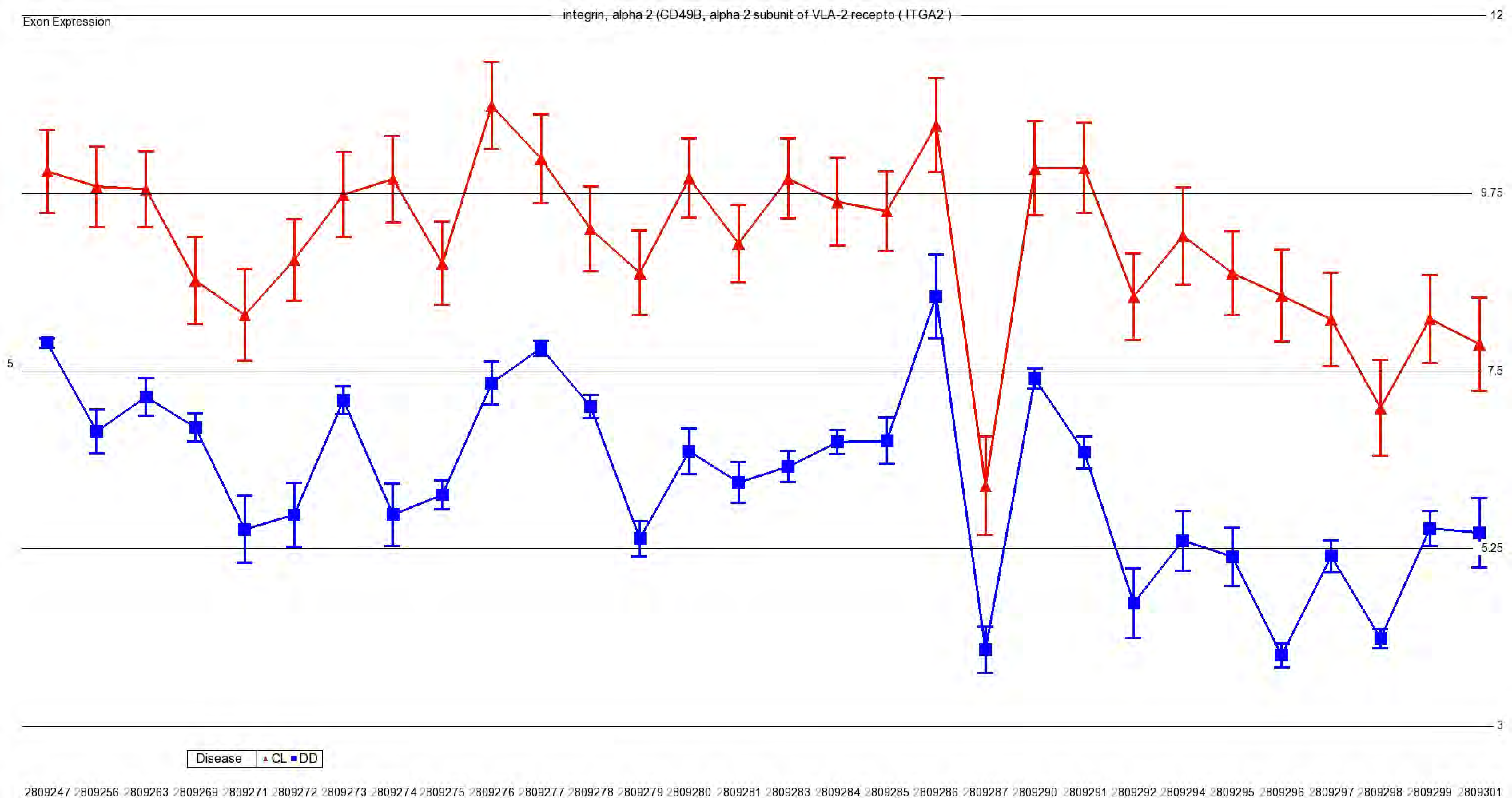

Exon Expression
integrin, alpha 2 (CD49B, alpha 2 subunit of VLA-2 recepto (ITGA2)
12
9.75
7.5
5.25
3
5
Disease
CL
DD
2809247
2809256
2809263
2809269
2809271
2809272
2809273
2809274
2809275
2809276
2809277
2809278
2809279
2809280
2809281
2809283
2809284
2809285
2809286
2809287
2809290
2809291
2809292
2809294
2809295
2809296
2809297
2809298
2809299
2809301

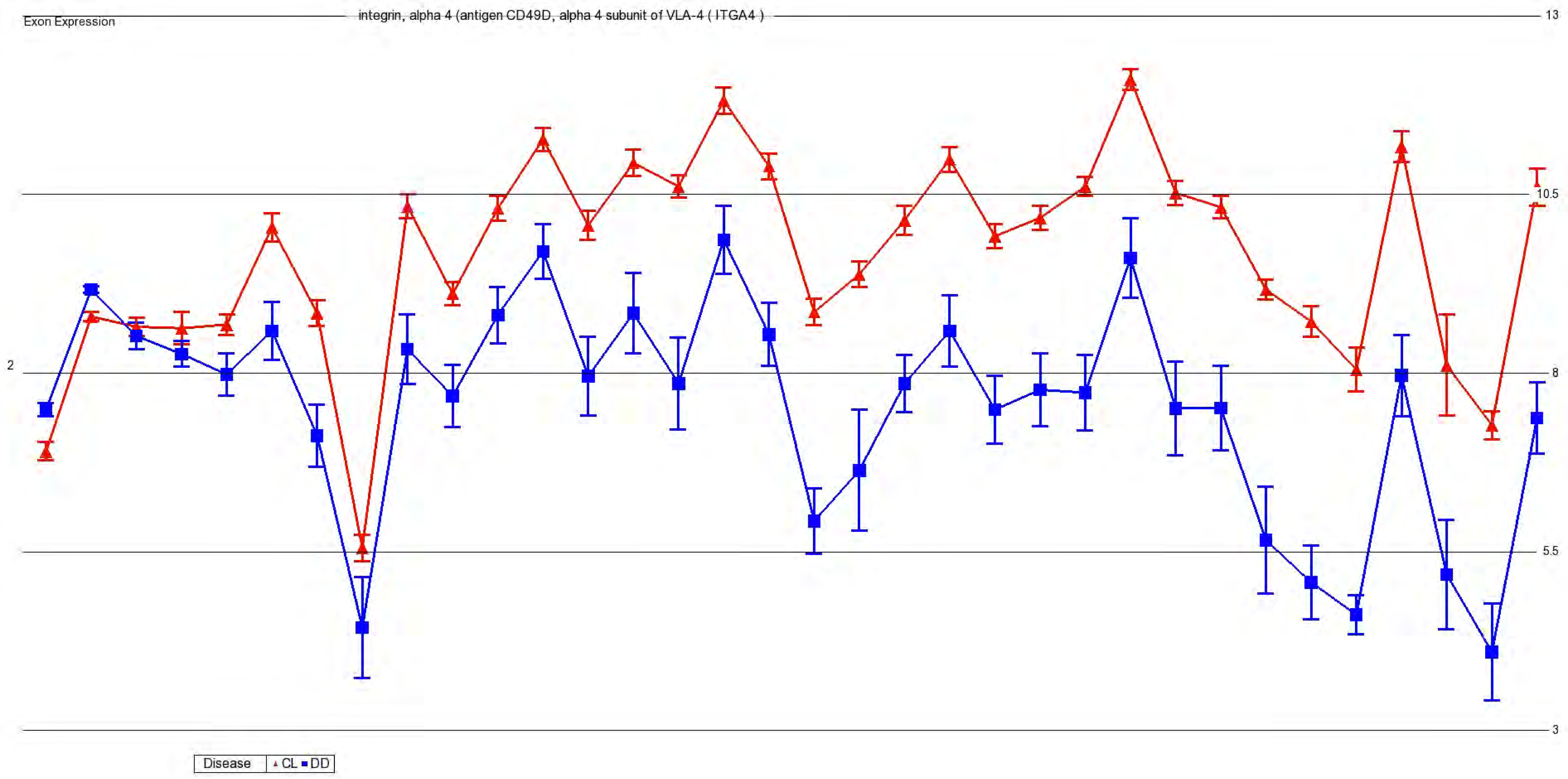
integrin, alpha 4 (antigen CD49D, alpha 4 subunit of VLA-4 (ITGA4)
Exon Expression
13
10.5
8
5.5
3
2
Disease
CL
DD

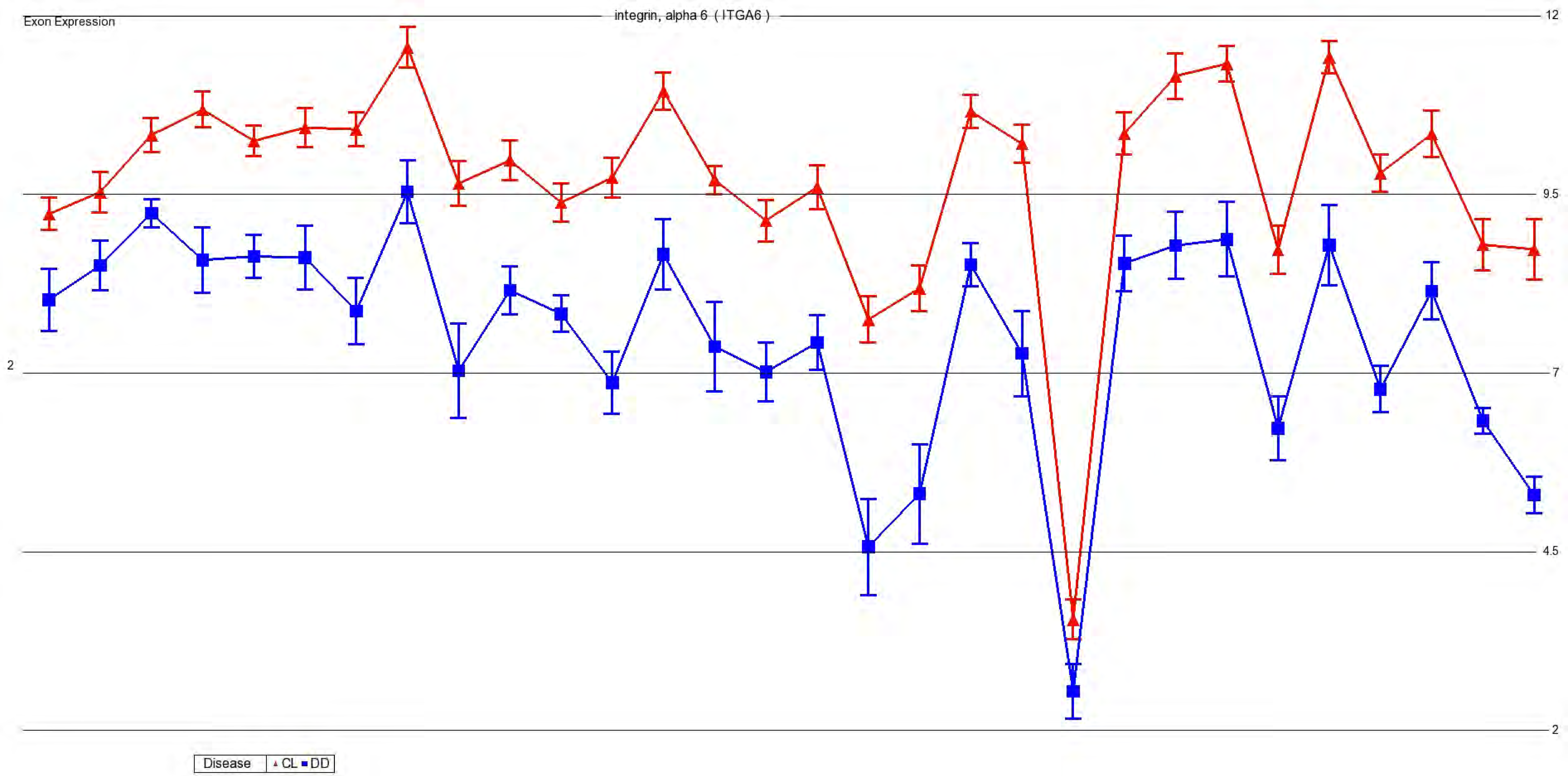

integrin, alpha 6 (ITGA6)
Exon Expression
12
9.5
7
4.5
2
2
Disease
CL
DD

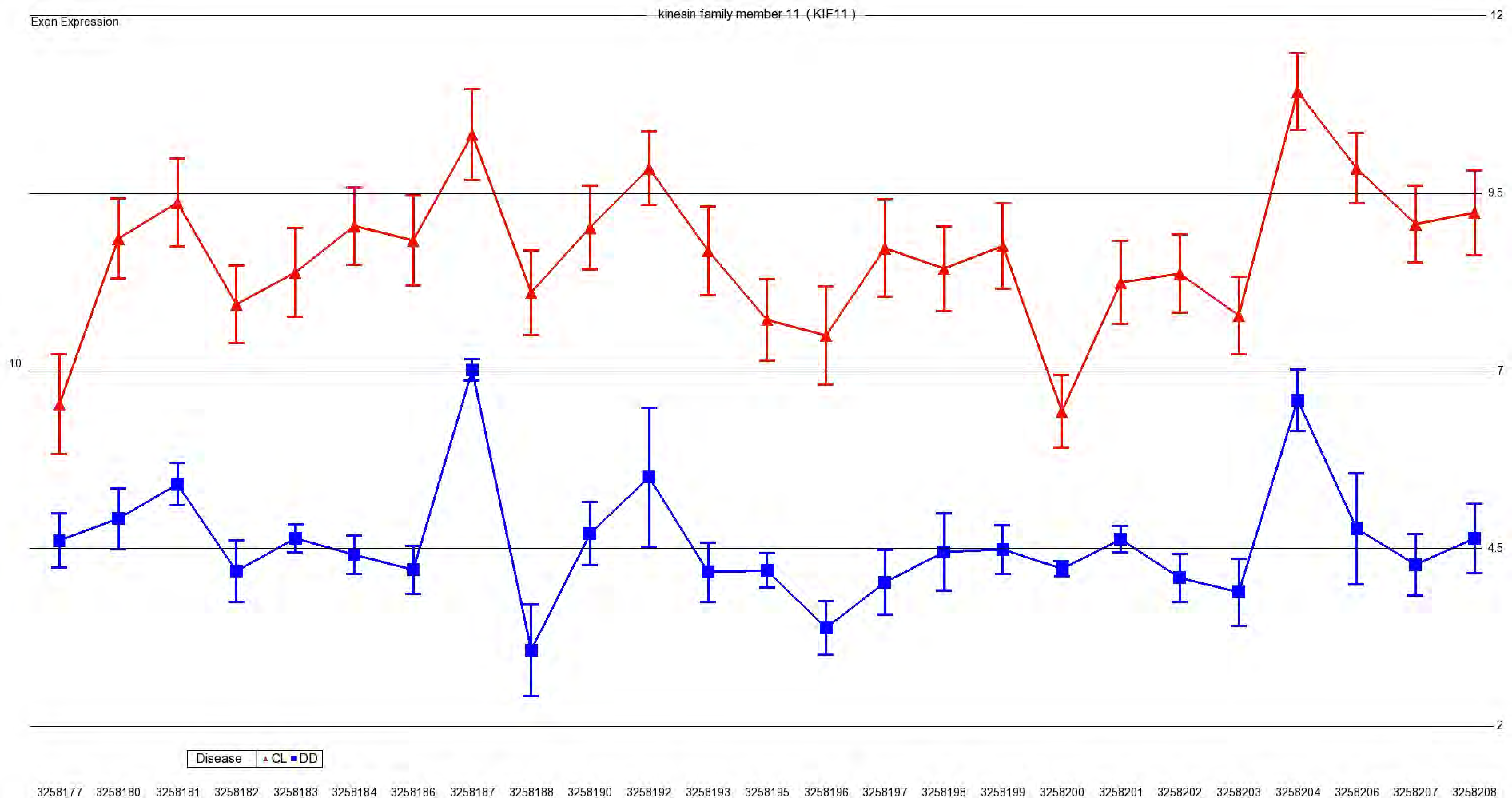
kinesin family member 11 (KIF11)
Exon Expression
12
9.5
10
7
4.5
2
Disease
CL
DD
3258177
3258180
3258181
3258182
3258183
3258184
3258186
3258187
3258188
3258190
3258192
3258193
3258195
3258196
3258197
3258198
3258199
3258200
3258201
3258202
3258203
3258204
3258206
3258207
3258208

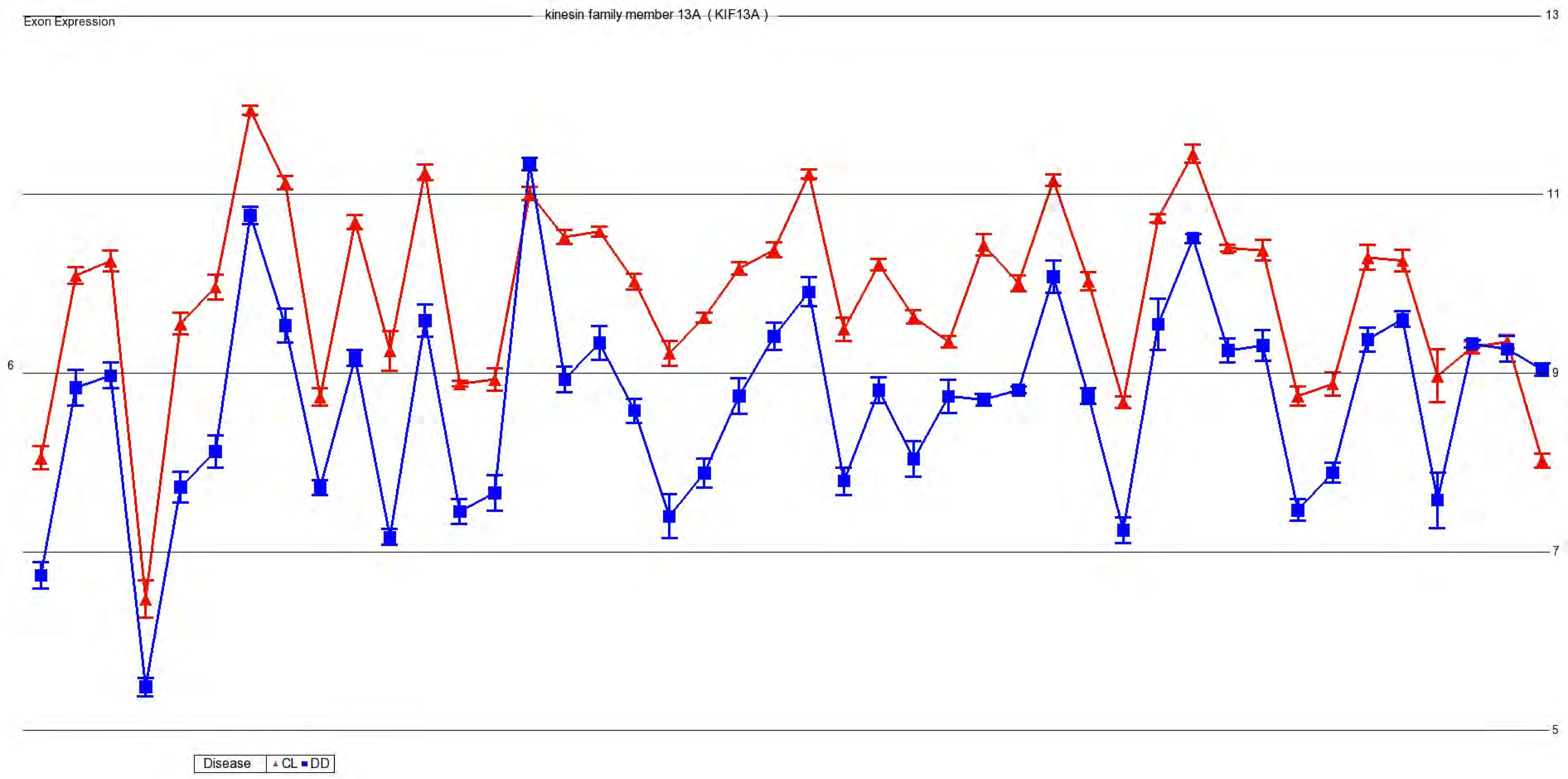

kinesin family member 13A (KIF13A)
Exon Expression
13
11
9
7
5
6
Disease
CL
DD

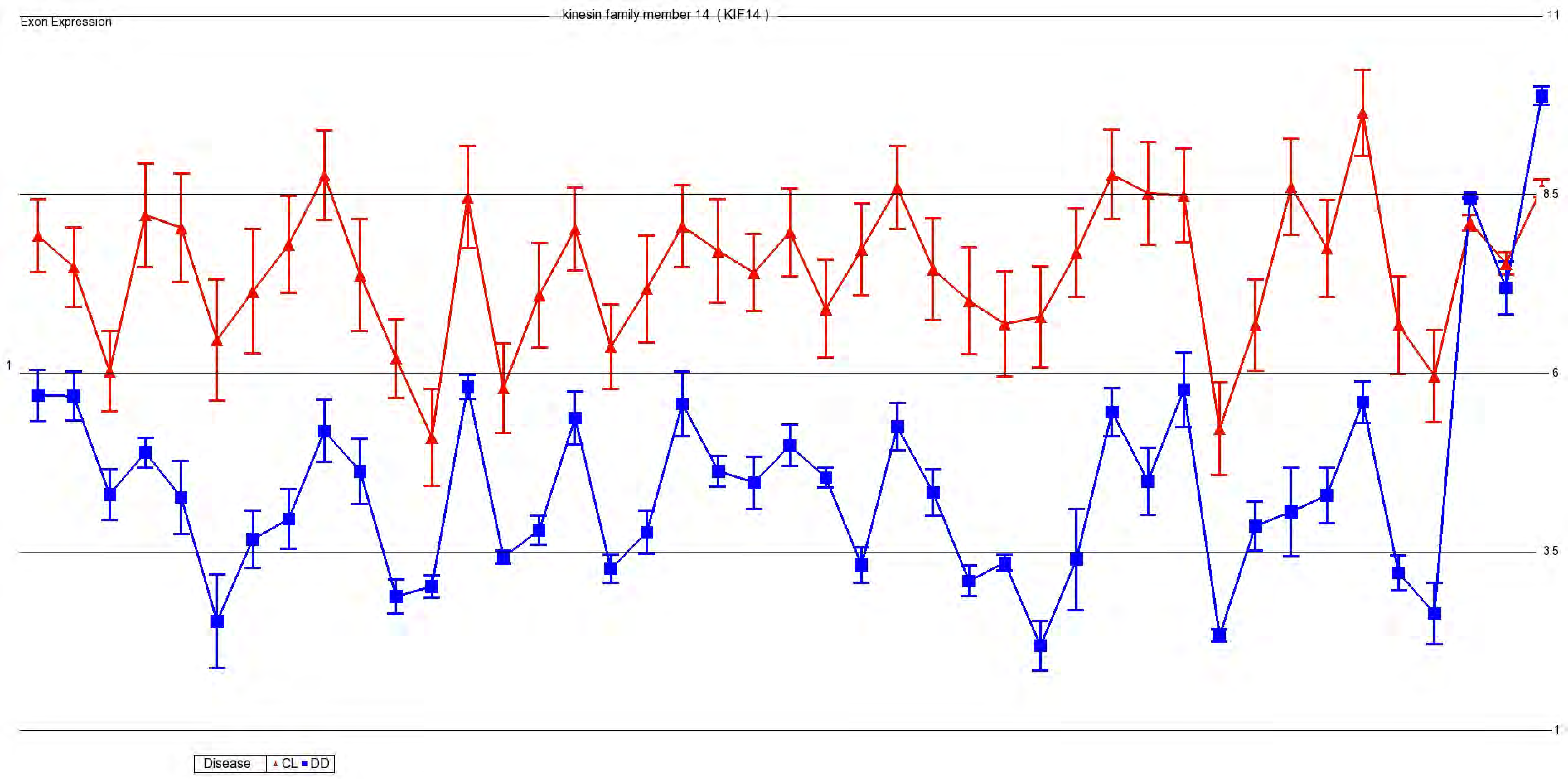
kinesin family member 14 (KIF14)
Exon Expression
11
8.5
6
3.5
1
1
Disease
CL
DD

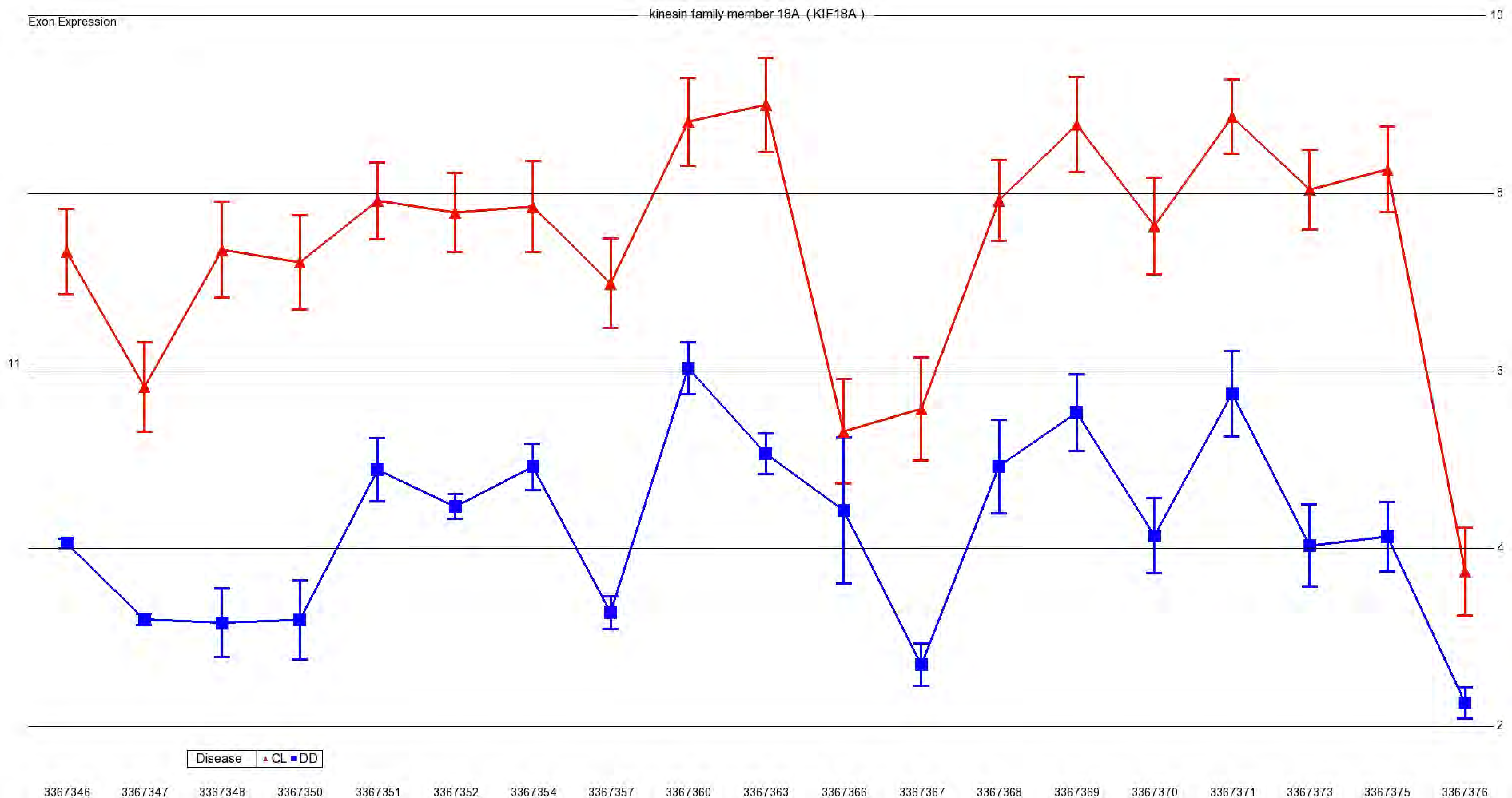

kinesin family member 18A (KIF18A)
Exon Expression
10
8
11
6
4
2
Disease
CL
DD
3367346
3367347
3367348
3367350
3367351
3367352
3367354
3367357
3367360
3367363
3367366
3367367
3367368
3367369
3367370
3367371
3367373
3367375
3367376

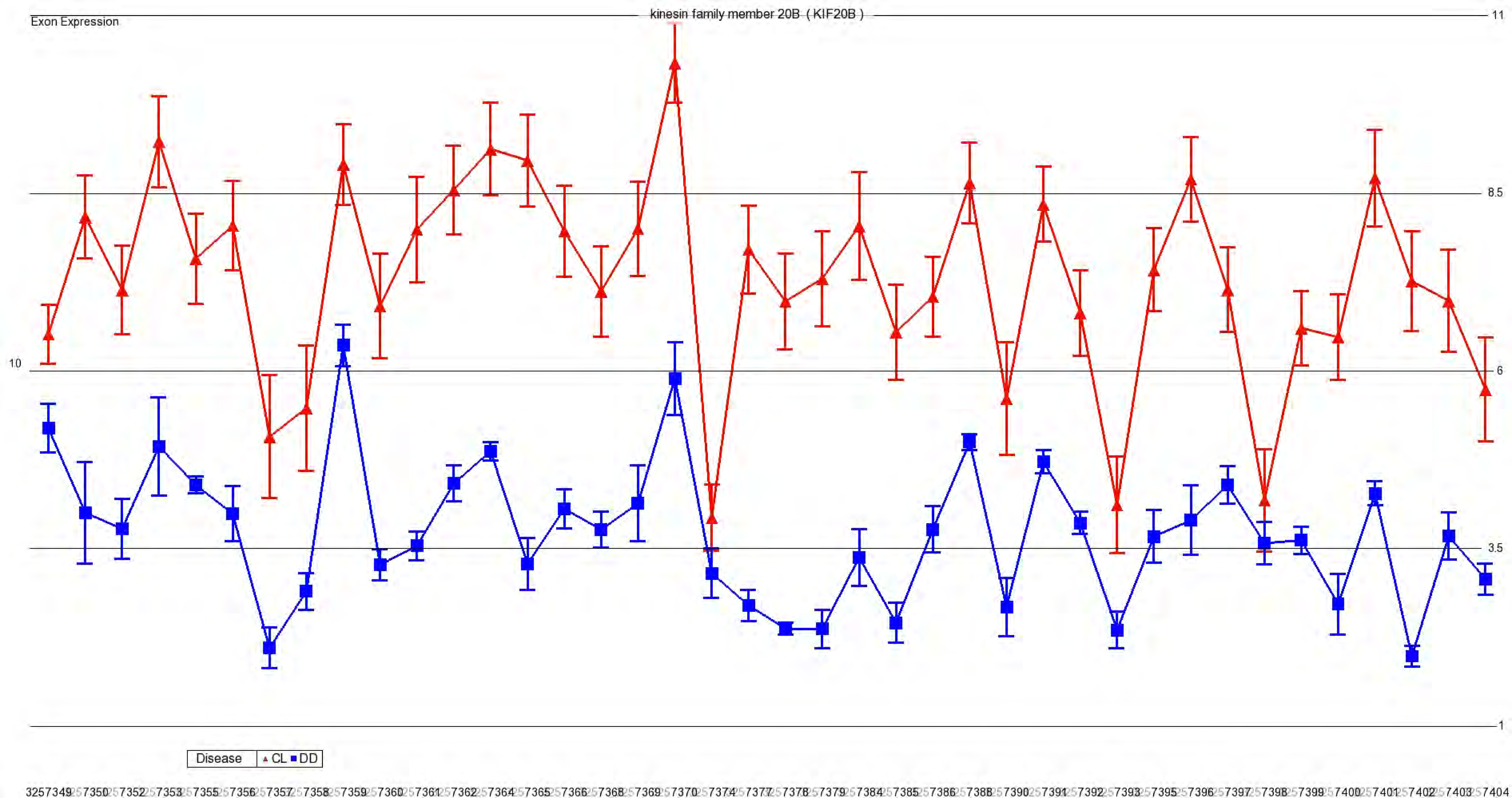

kinesin family member 20B (KIF20B)
Exon Expression
11
8.5
10
6
3.5
1
Disease
CL
DD
3257349
3257350
3257352
3257353
3257355
3257356
3257357
3257358
3257359
3257360
3257361
3257362
3257364
3257365
3257366
3257368
3257369
3257370
3257374
3257377
3257378
3257379
3257384
3257385
3257386
3257388
3257390
3257391
3257392
3257393
3257395
3257396
3257397
3257398
3257399
3257400
3257401
3257402
3257403
3257404

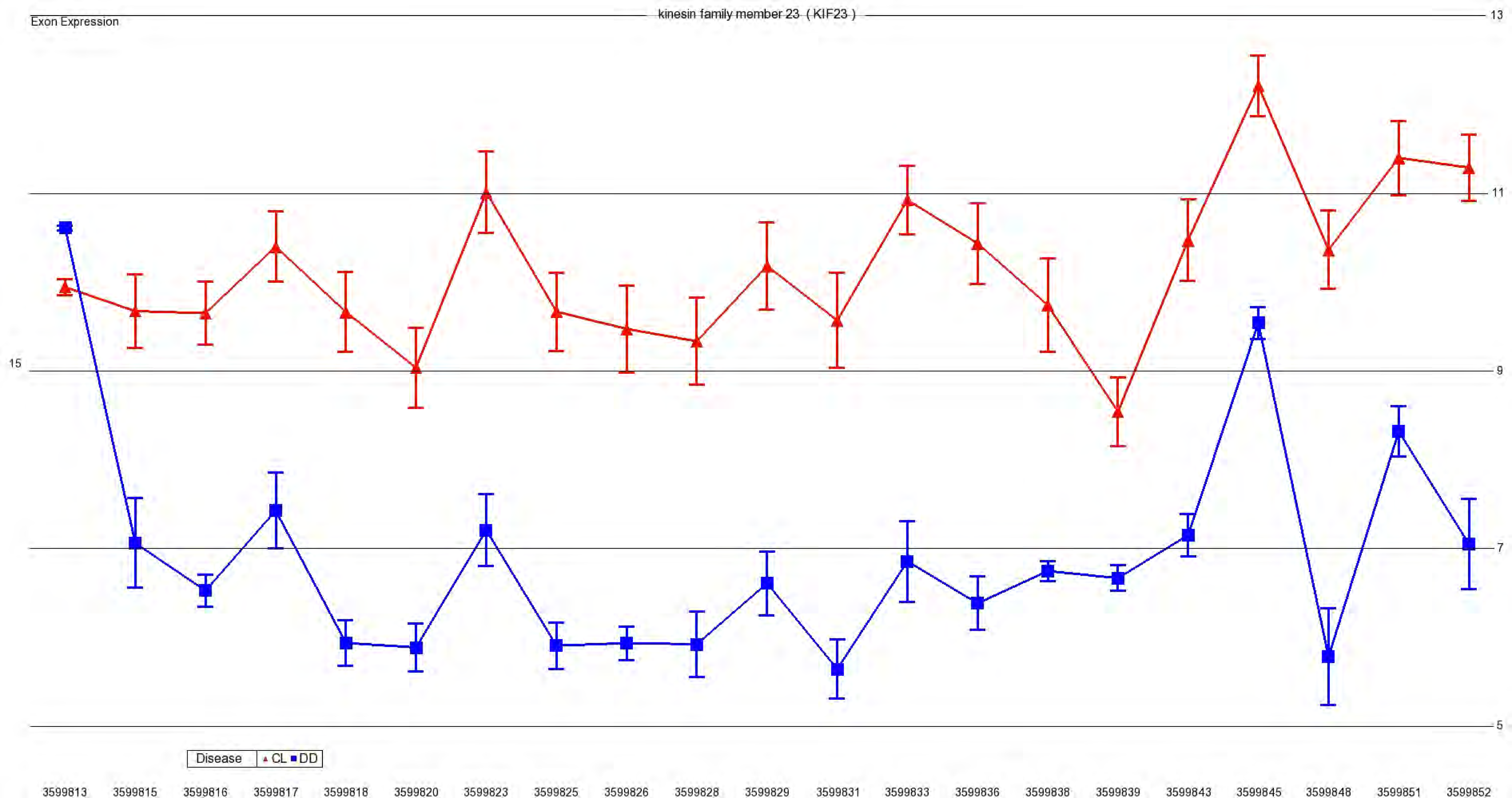

kinesin family member 23 (KIF23)
Exon Expression
13
11
9
7
5
15
Disease
CL
DD
3599813
3599815
3599816
3599817
3599818
3599820
3599823
3599825
3599826
3599828
3599829
3599831
3599833
3599836
3599838
3599839
3599843
3599845
3599848
3599851
3599852

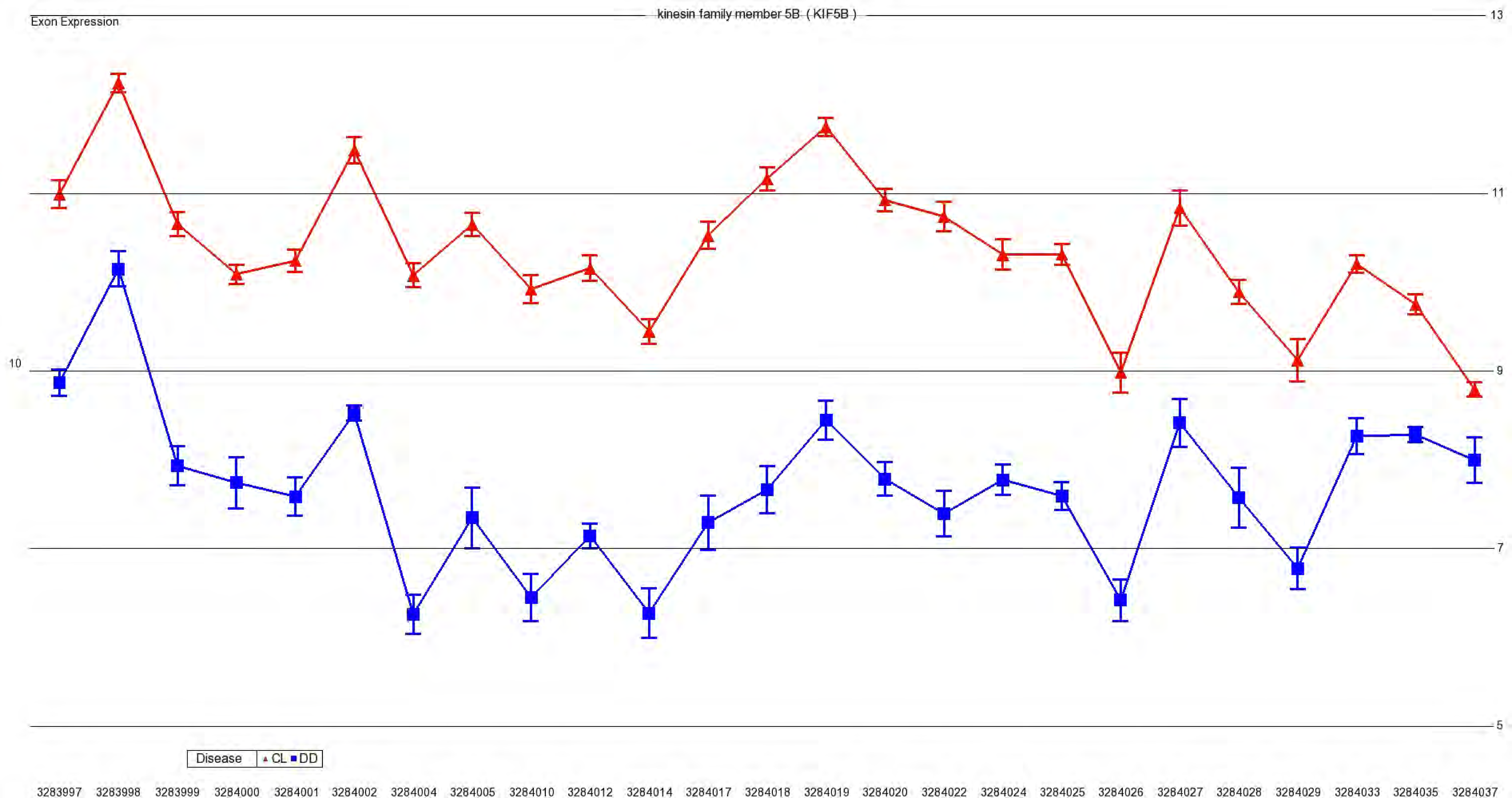

kinesin family member 5B (KIF5B)
Exon Expression
13
11
10
9
7
5
Disease
CL
DD
3283997
3283998
3283999
3284000
3284001
3284002
3284004
3284005
3284010
3284012
3284014
3284017
3284018
3284019
3284020
3284022
3284024
3284025
3284026
3284027
3284028
3284029
3284033
3284035
3284037

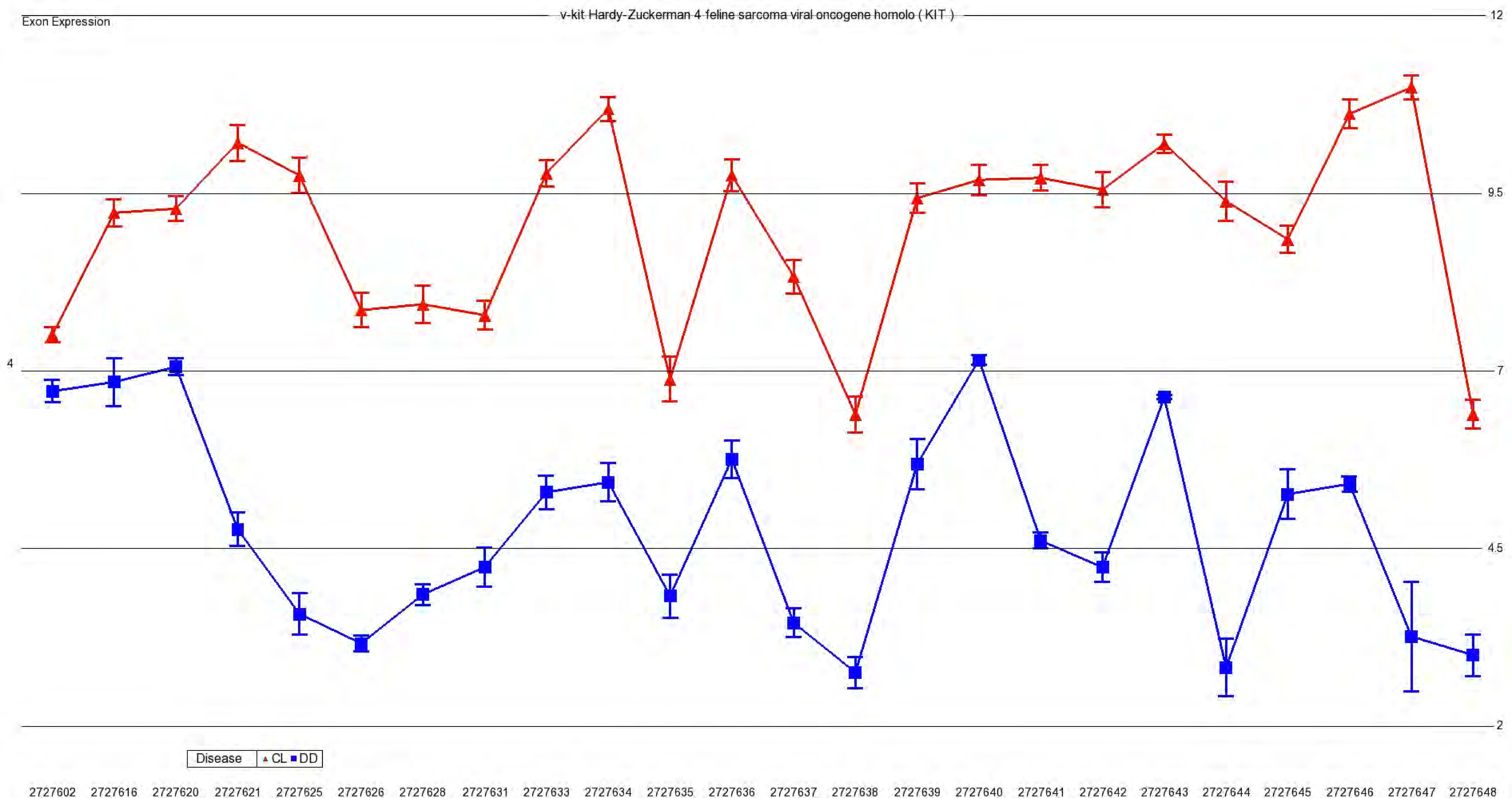

v-kit Hardy-Zuckerman 4 feline sarcoma viral oncogene homolo (KIT)
Exon Expression
12
9.5
7
4.5
2
4
Disease
CL
DD
2727602
2727616
2727620
2727621
2727625
2727626
2727628
2727631
2727633
2727634
2727635
2727636
2727637
2727638
2727639
2727640
2727641
2727642
2727643
2727644
2727645
2727646
2727647
2727648

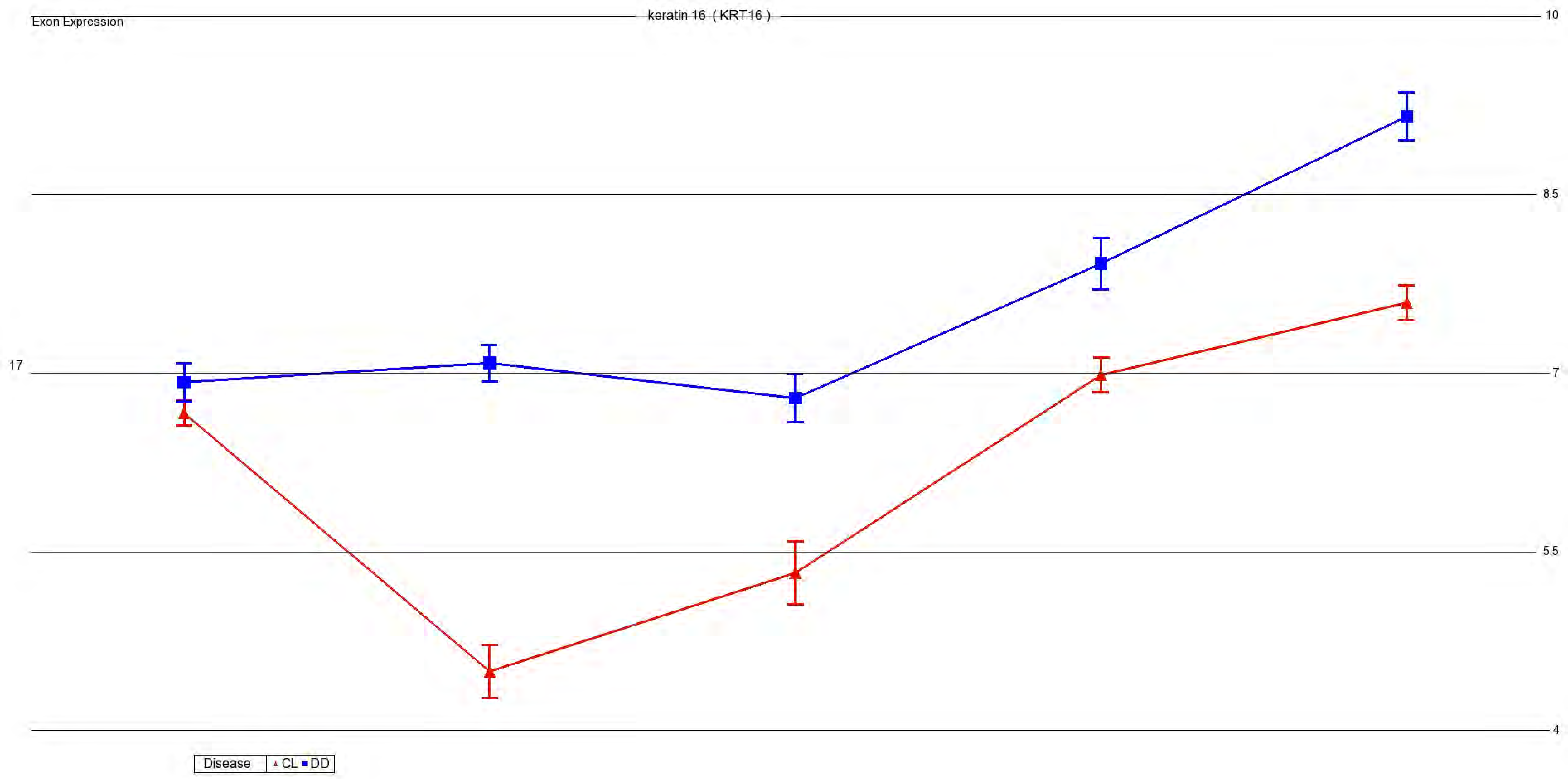
keratin 16 (KRT16)
Exon Expression
10
8.5
17
7
5.5
4
Disease
CL
DD

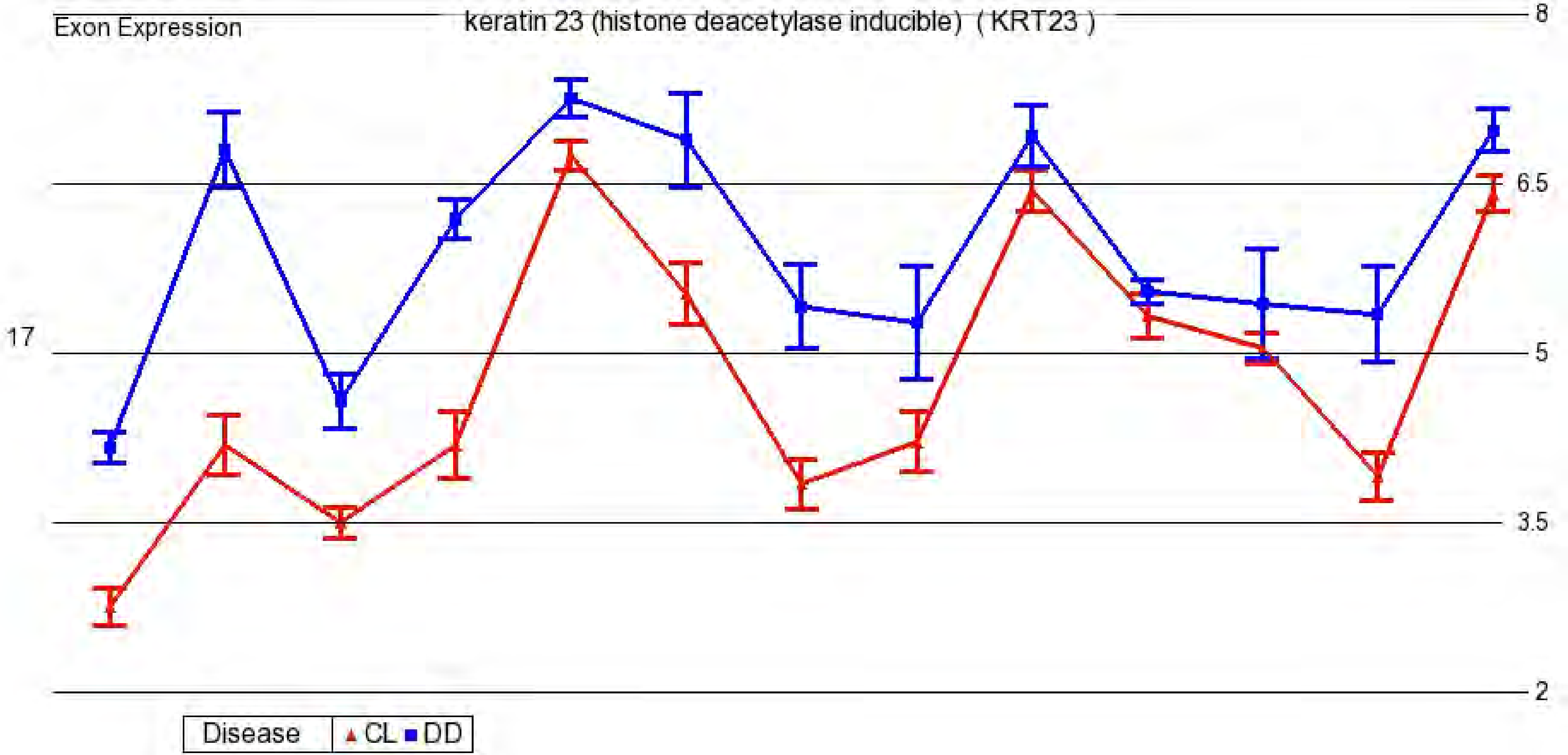

Exon Expression
keratin 23 (histone deacetylase inducible) (KRT23)
17
8
6.5
5
3.5
2
Disease
CL
DD

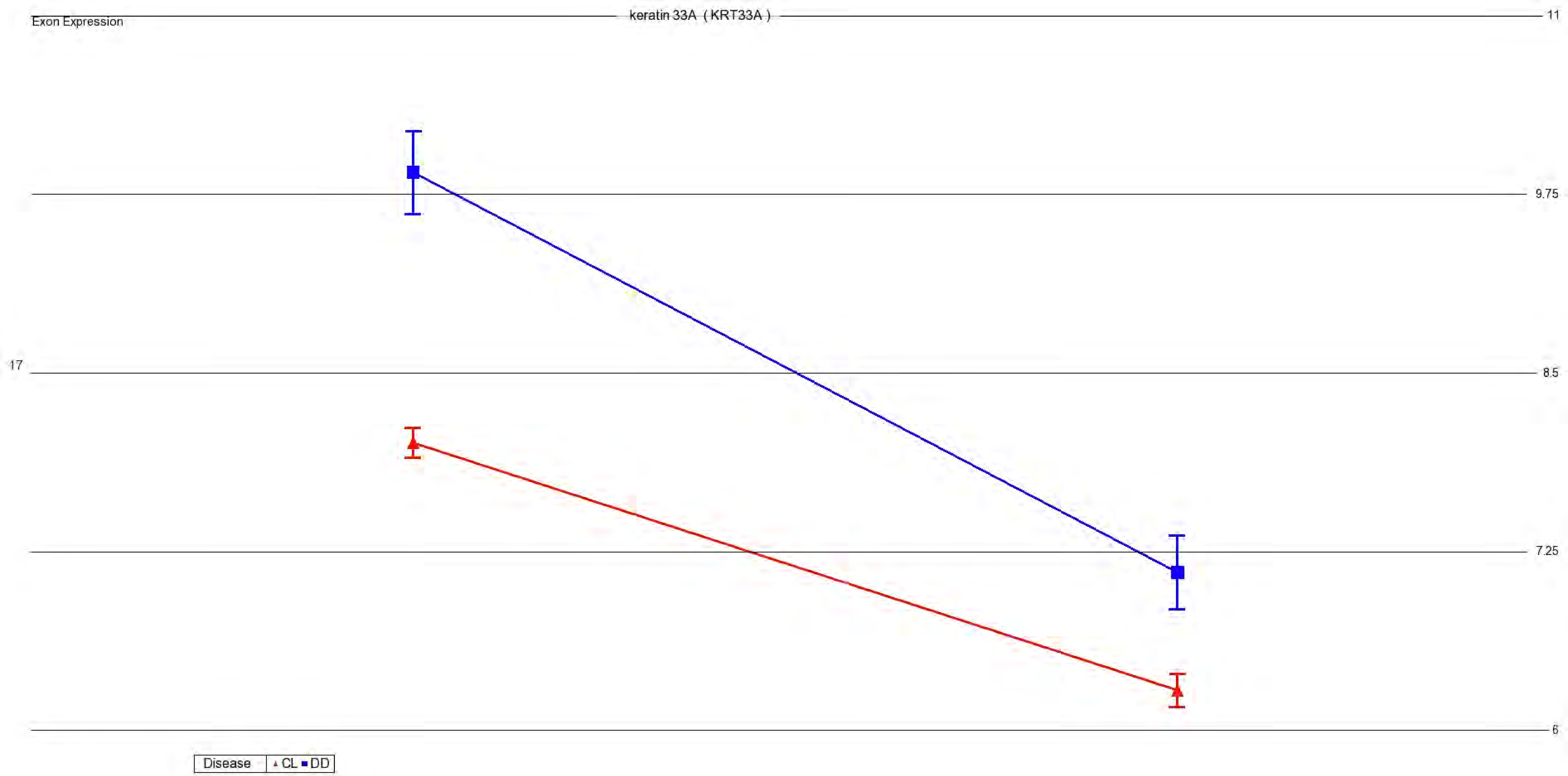

keratin 33A (KRT33A)
Exon Expression
11
9.75
8.5
7.25
6
17
Disease
CL
DD

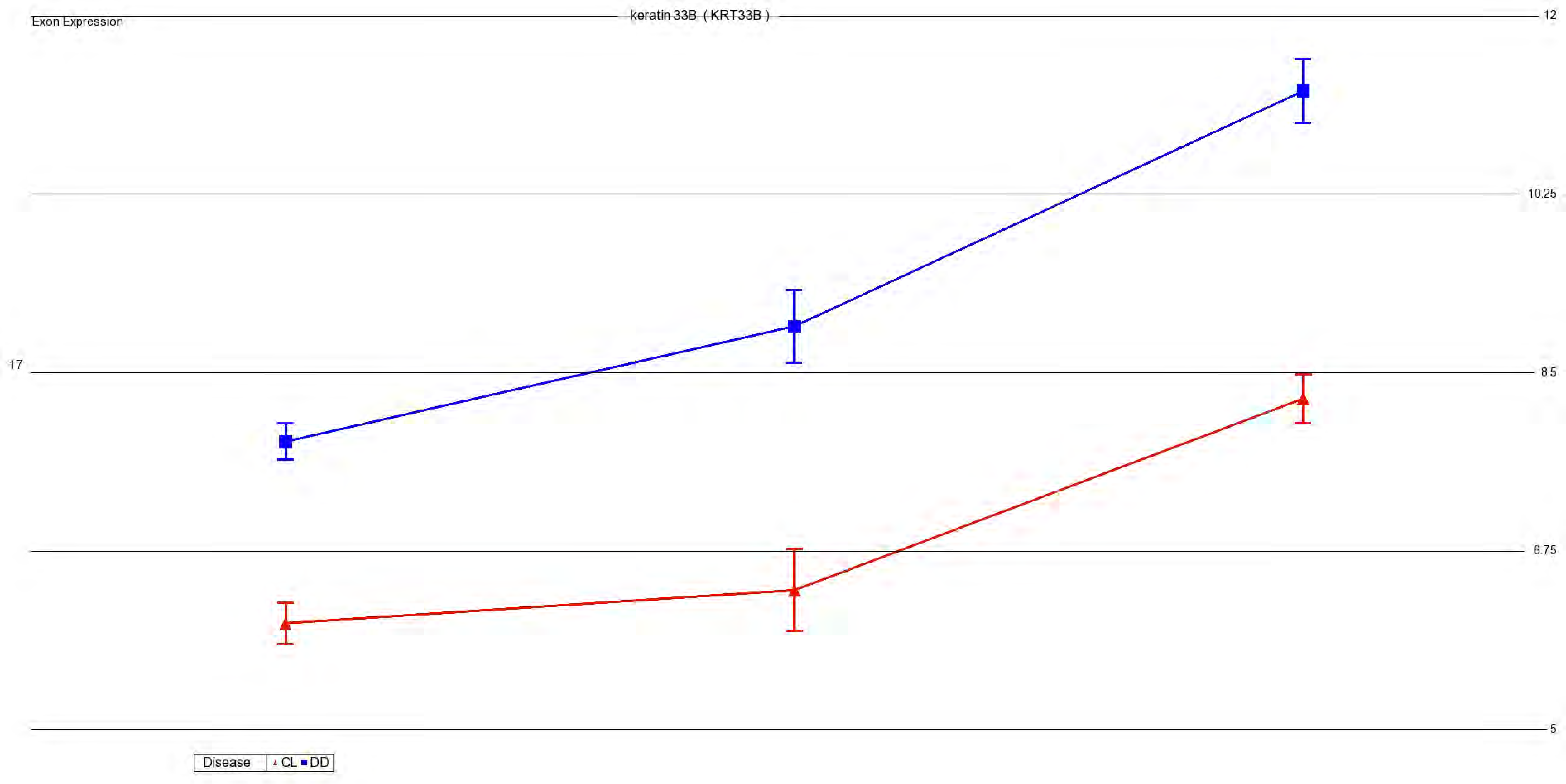
keratin 33B (KRT33B)
Exon Expression
12
10.25
17
8.5
6.75
5
Disease
CL
DD

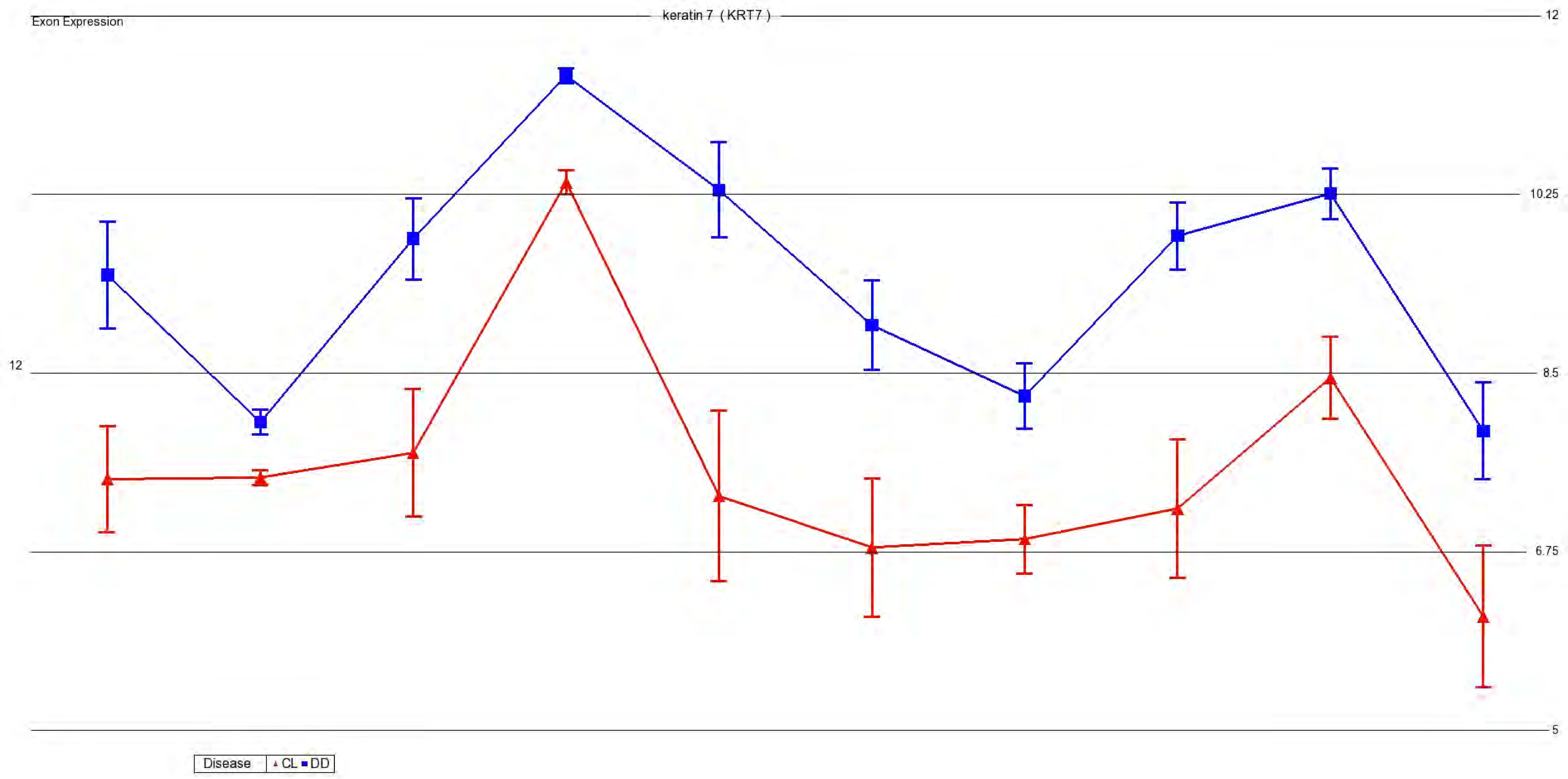
keratin 7 (KRT7)
Exon Expression
12
10.25
8.5
6.75
5
12
Disease
CL
DD

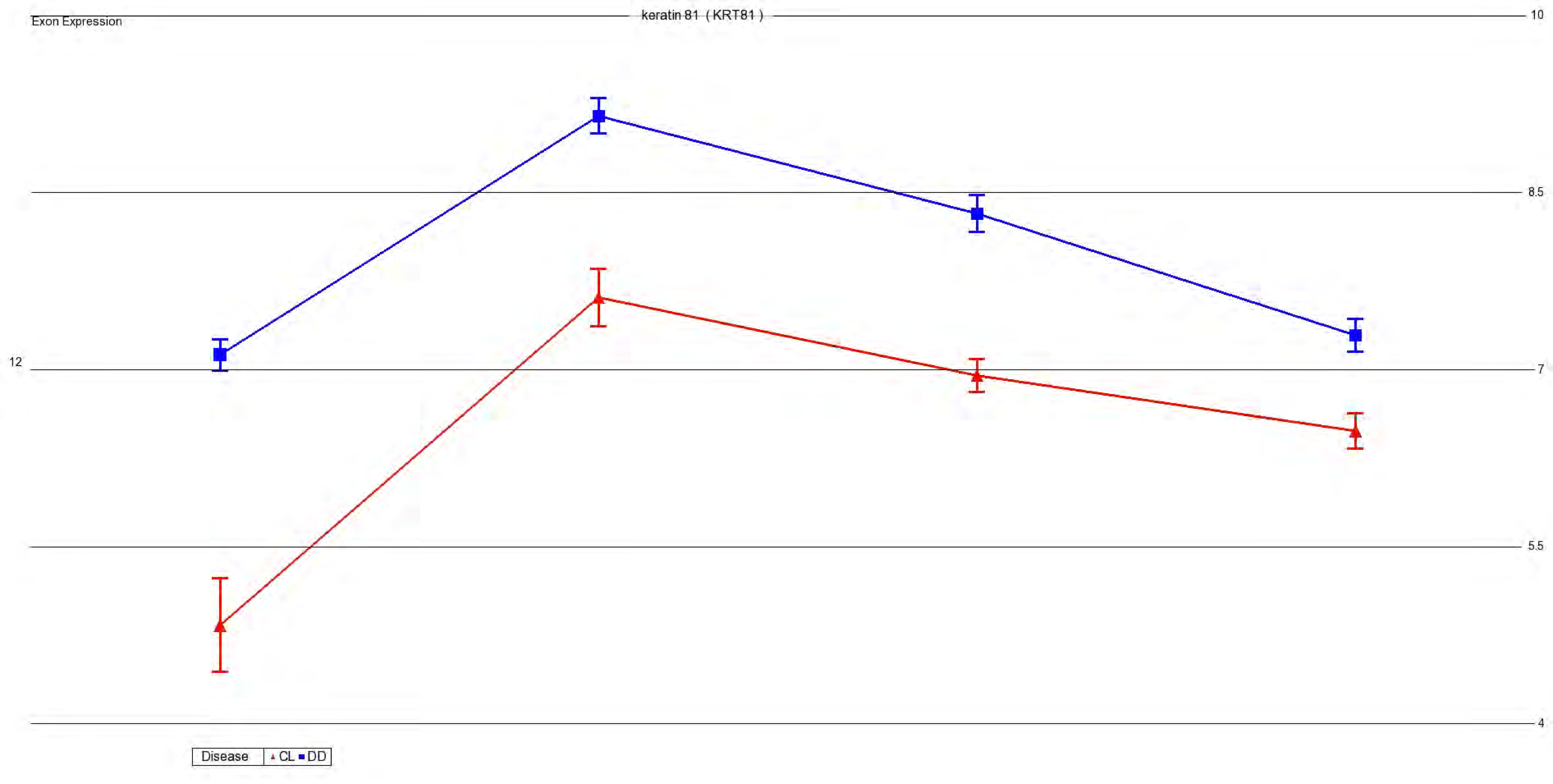
Exon Expression
keratin 81 (KRT81)
10
8.5
12
7
5.5
4
Disease
CL
DD

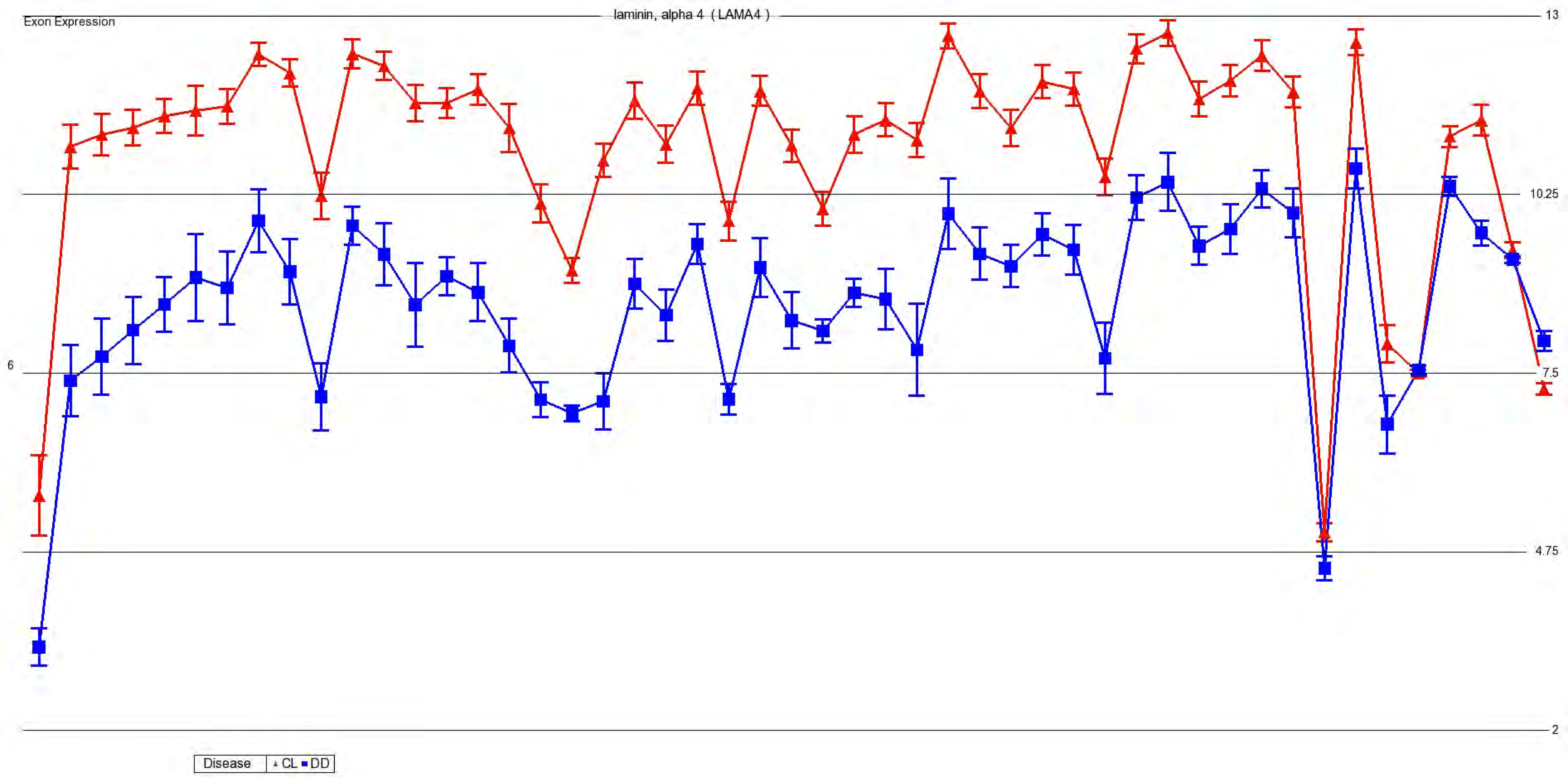
laminin, alpha 4 (LAMA4)
Exon Expression
13
10.25
7.5
4.75
2
6
Disease
CL
DD

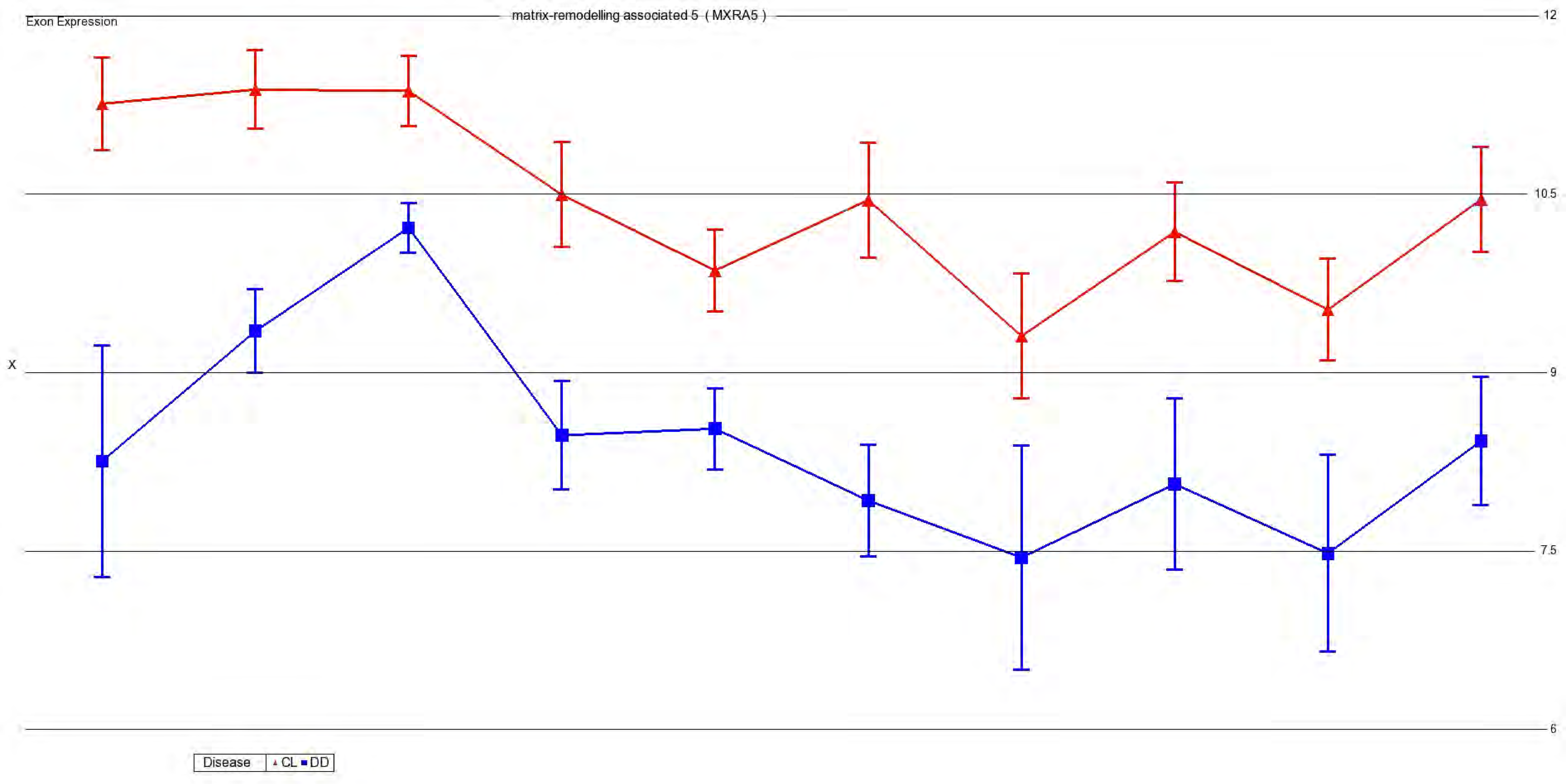

matrix-remodelling associated 5 (MXRA5)
Exon Expression
X
12
10.5
9
7.5
6
Disease
CL
DD

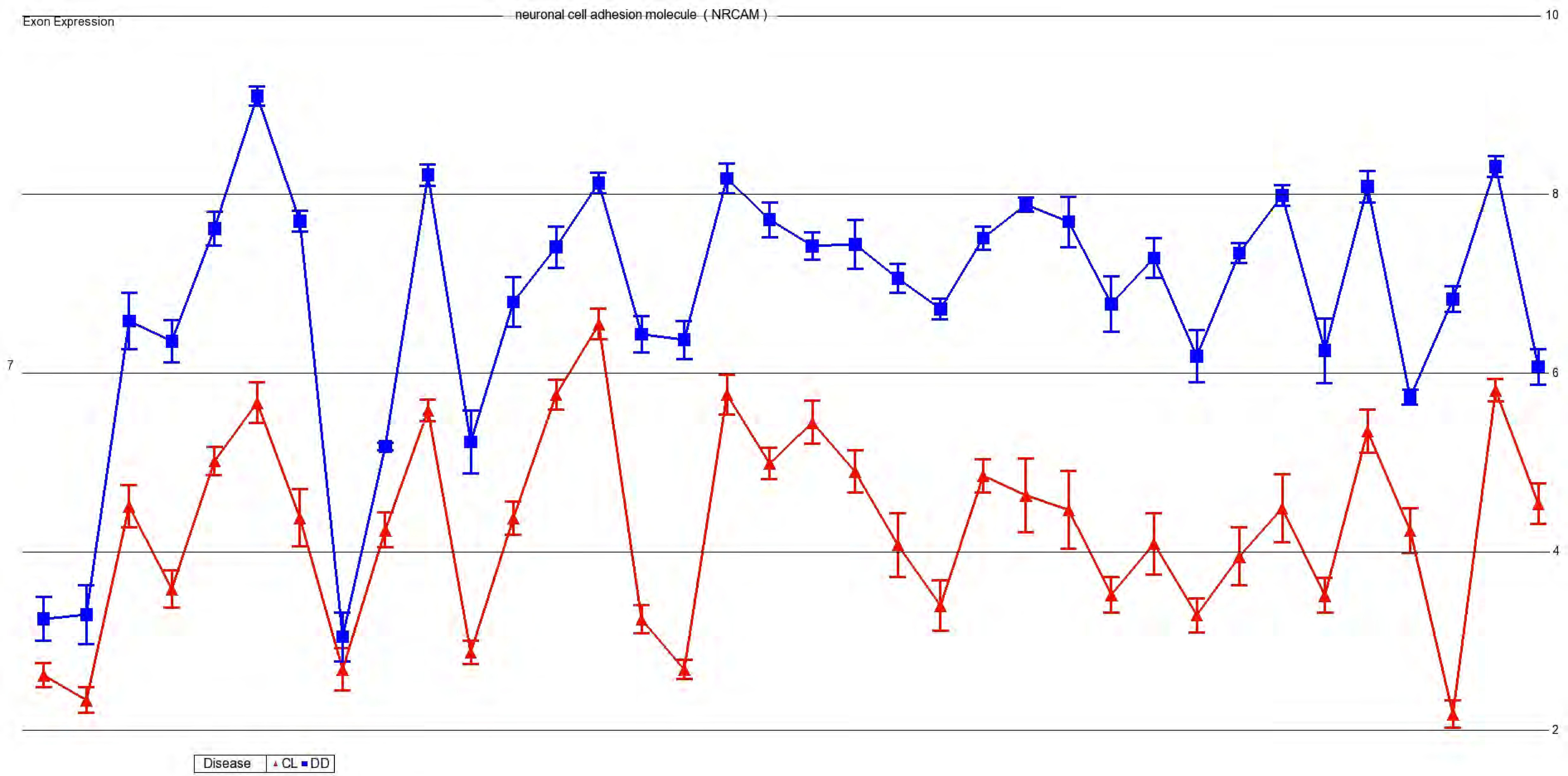
neuronal cell adhesion molecule (NRCAM)
Exon Expression
10
8
7
6
4
2
Disease
CL
DD

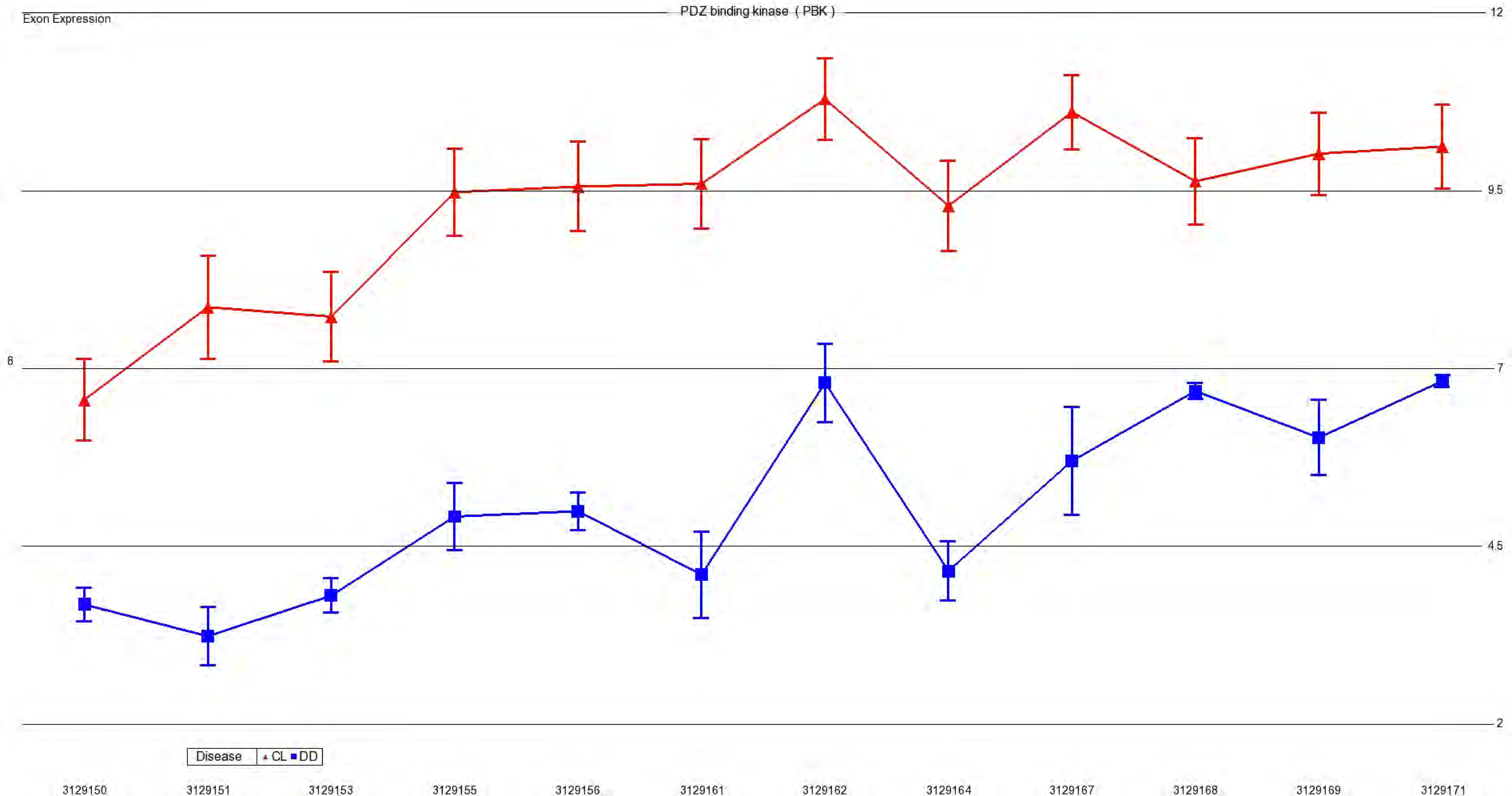
PDZ binding kinase (PBK)
Exon Expression
12
9.5
8
7
4.5
2
Disease
CL
DD
3129150
3129151
3129153
3129155
3129156
3129161
3129162
3129164
3129167
3129168
3129169
3129171

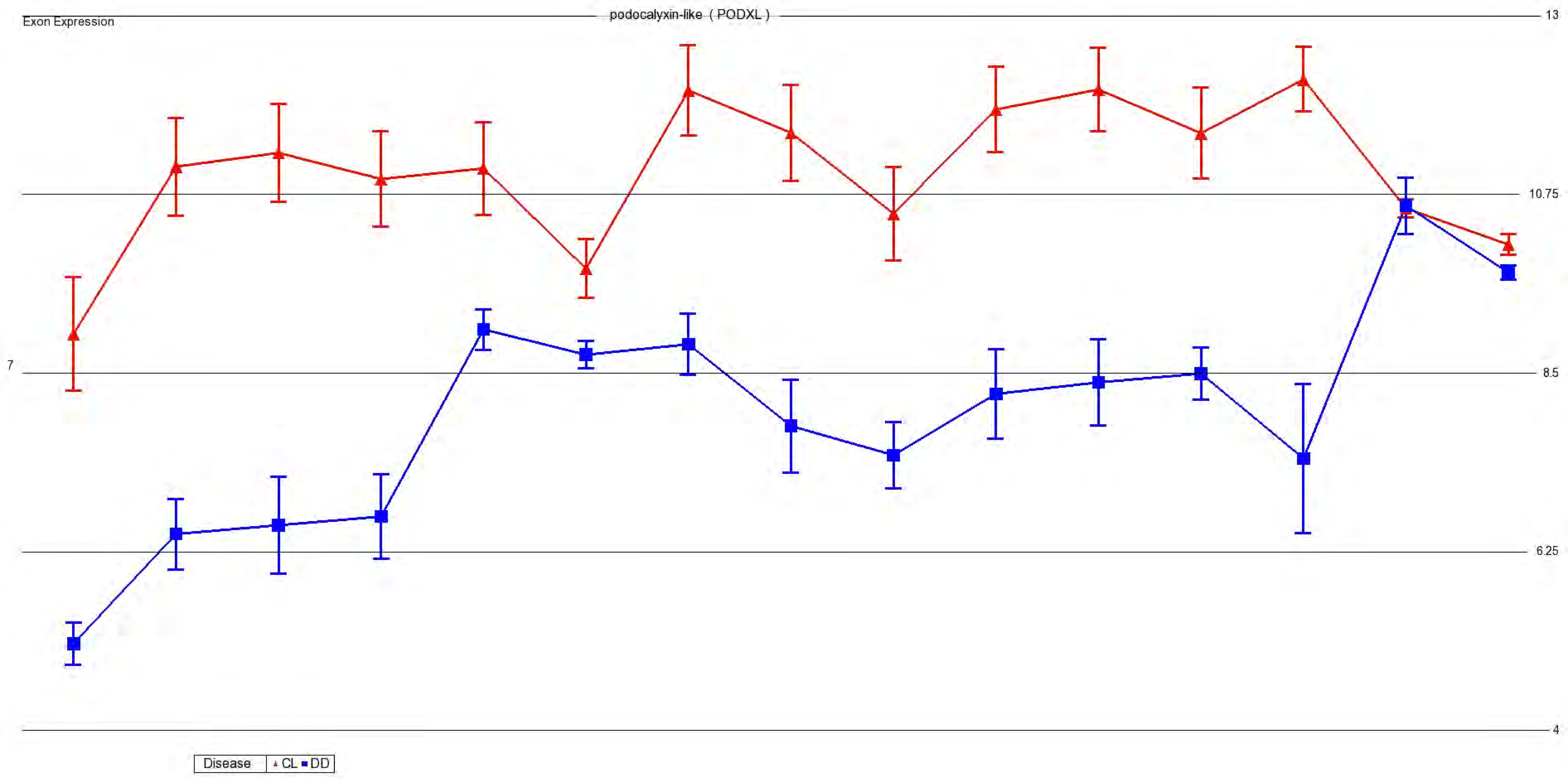

podocalyxin-like (PODXL)
Exon Expression
13
10.75
8.5
6.25
4
7
Disease
CL
DD

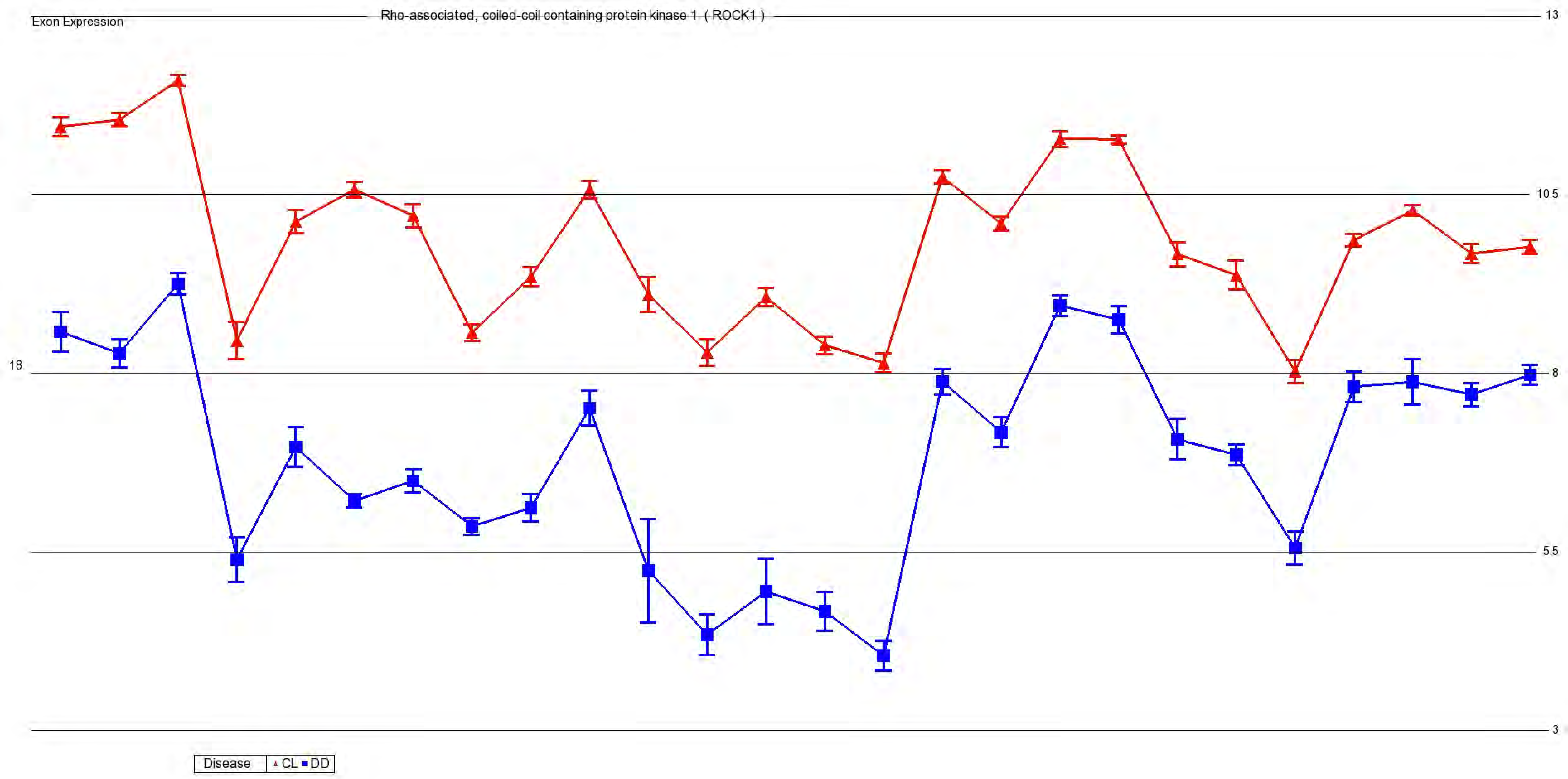

Rho-associated, coiled-coil containing protein kinase 1 (ROCK1)
Exon Expression
13
10.5
8
5.5
3
18
Disease
CL
DD

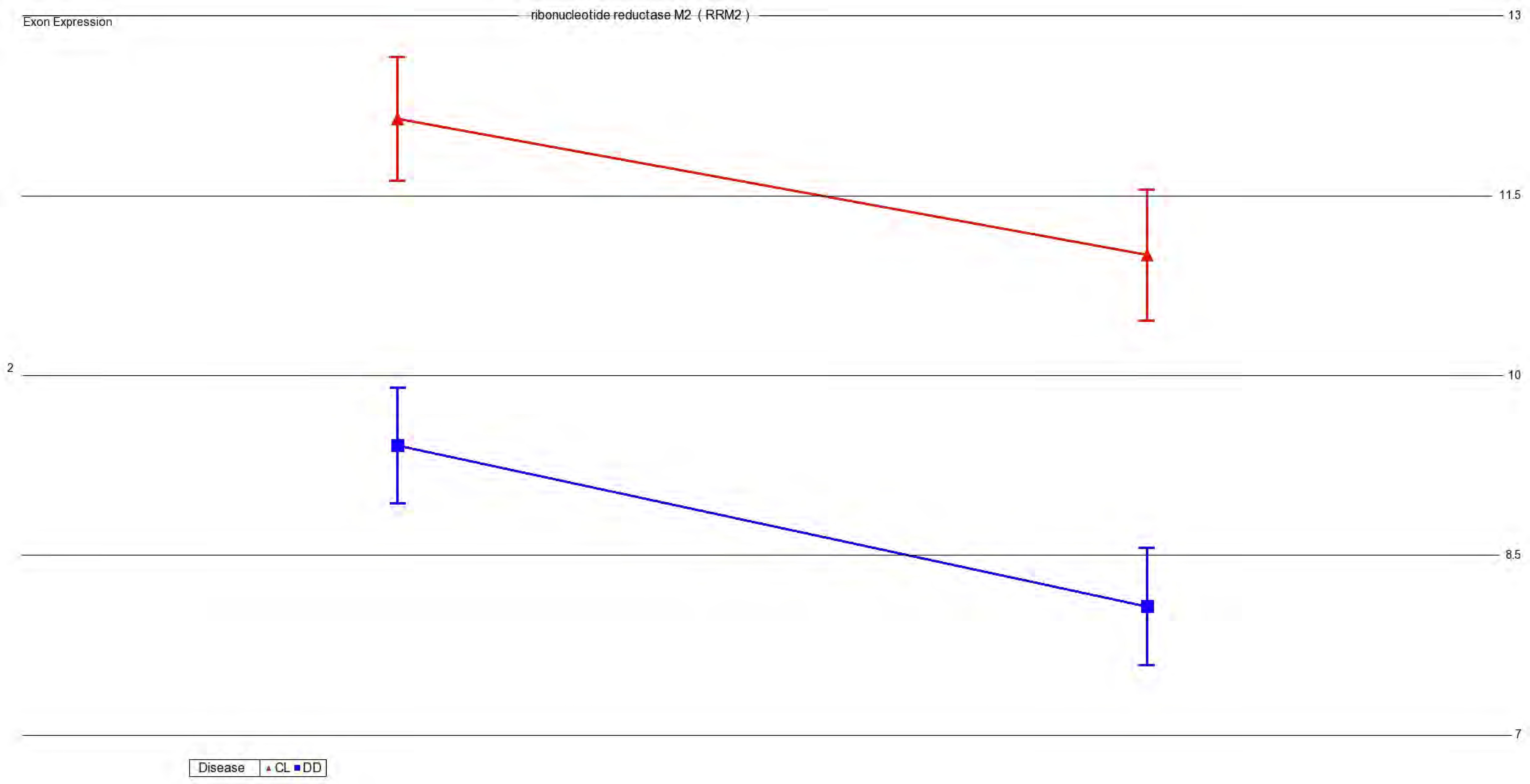

ribonucleotide reductase M2 (RRM2)
Exon Expression
13
11.5
2
10
8.5
7
Disease
CL
DD

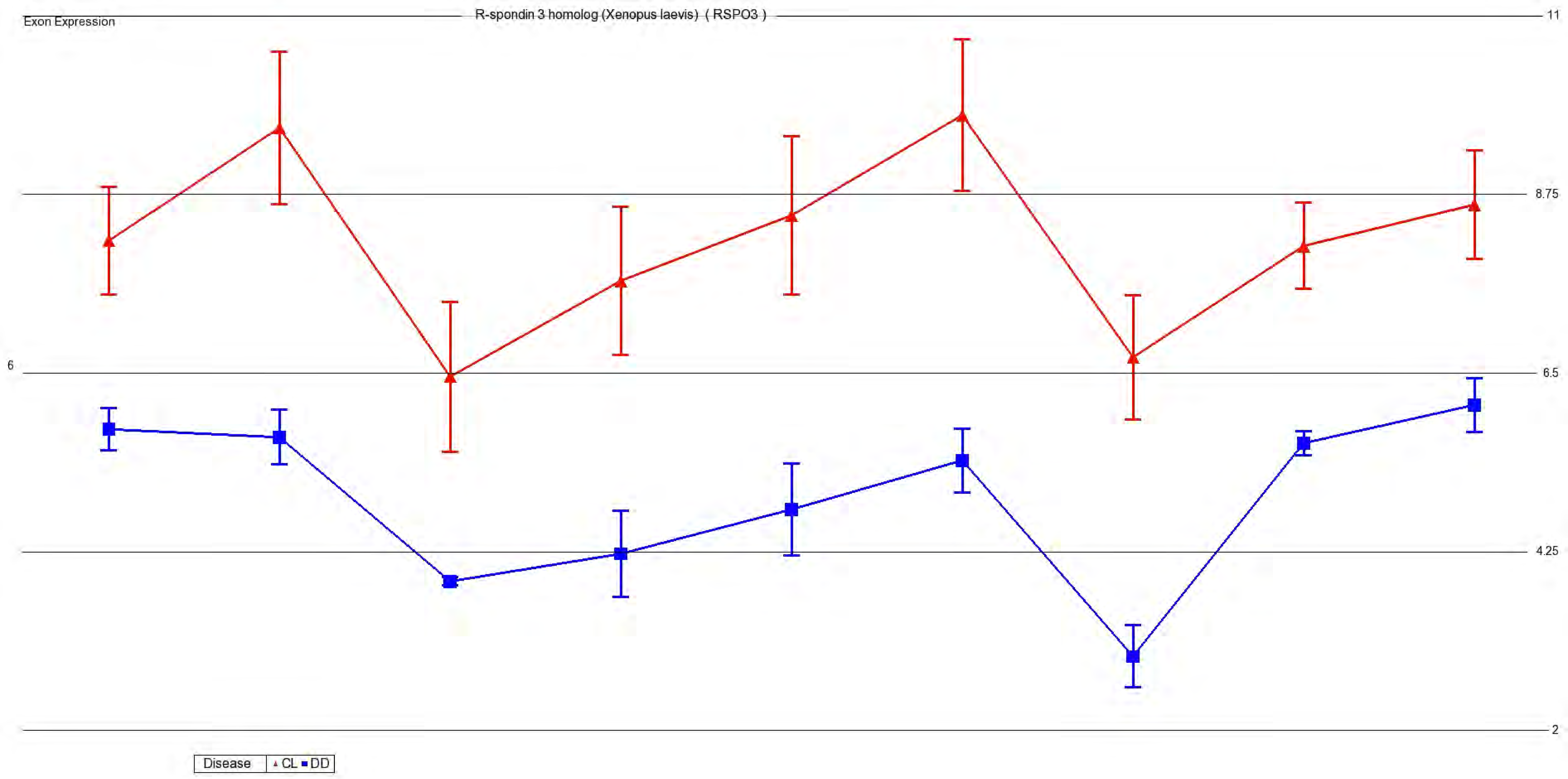

R-spondin 3 homolog (Xenopus laevis) (RSPO3)
Exon Expression
11
8.75
6
6.5
4.25
2
Disease
CL
DD

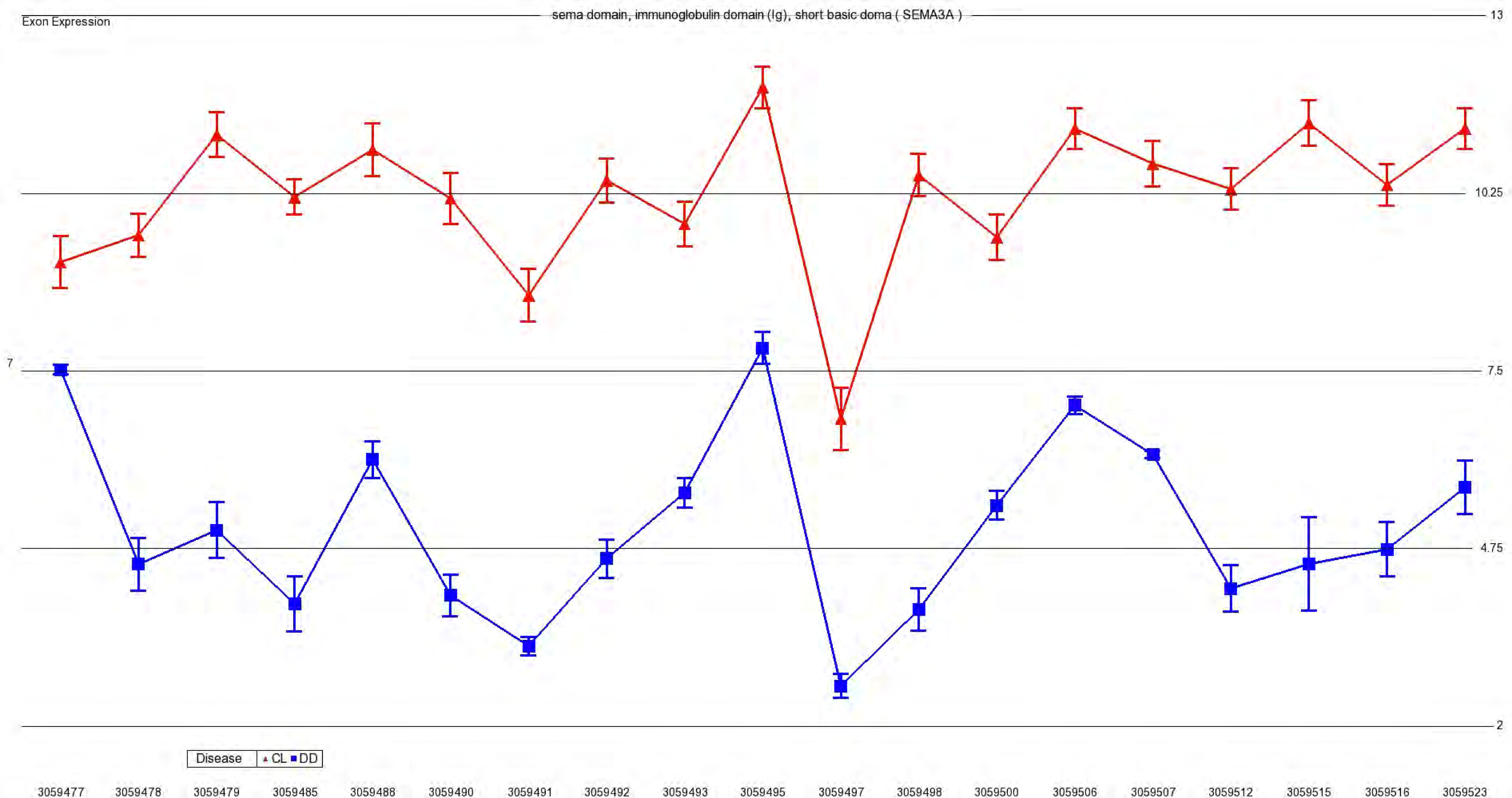

sema domain, immunoglobulin domain (Ig), short basic doma (SEMA3A)
Exon Expression
13
10.25
7.5
7
4.75
2
Disease
CL
DD
3059477
3059478
3059479
3059485
3059488
3059490
3059491
3059492
3059493
3059495
3059497
3059498
3059500
3059506
3059507
3059512
3059515
3059516
3059523

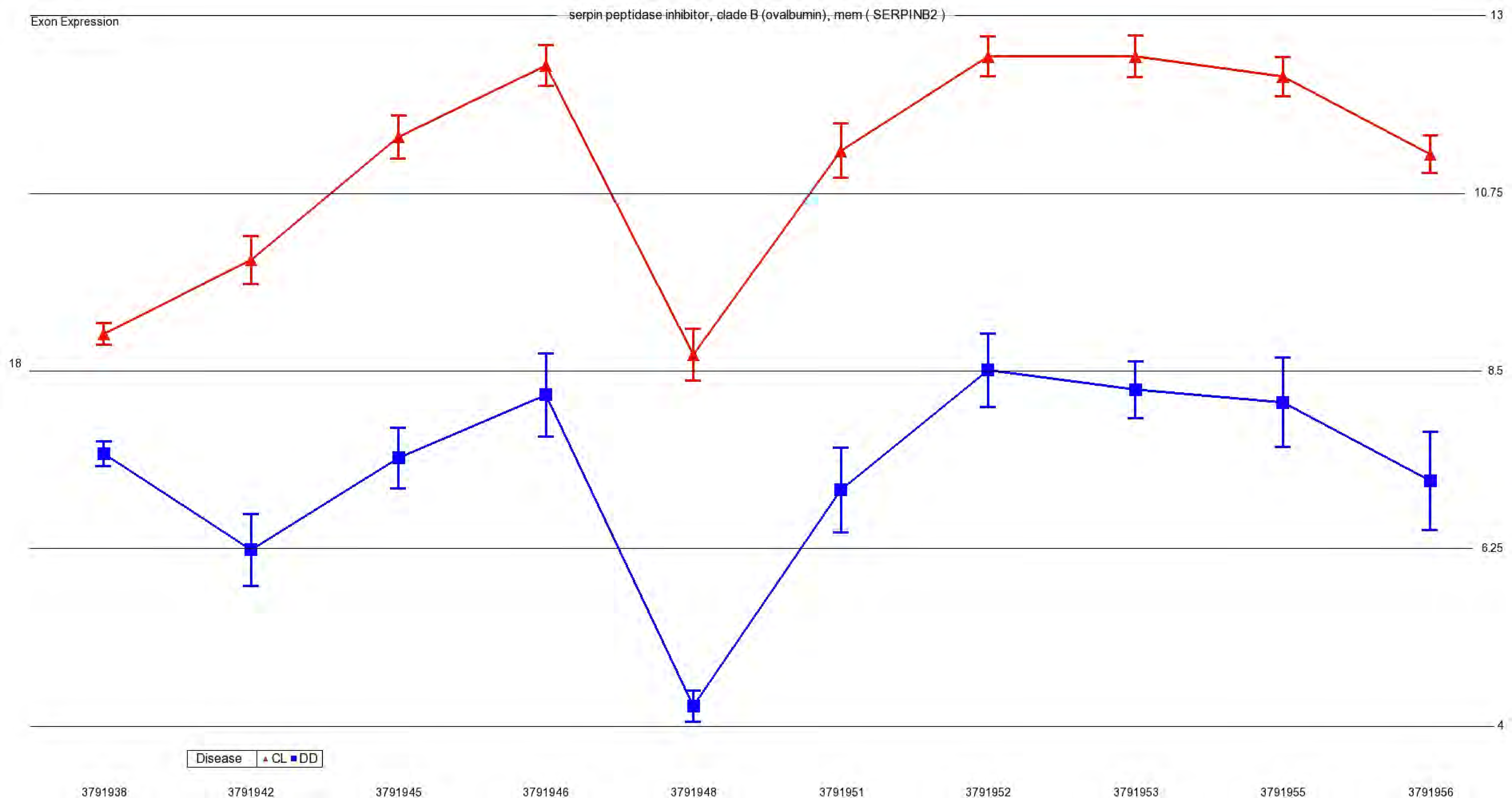
serpin peptidase inhibitor, clade B (ovalbumin), mem (SERPINB2)
Exon Expression
13
10.75
8.5
6.25
4
18
Disease
CL
DD
3791938
3791942
3791945
3791946
3791948
3791951
3791952
3791953
3791955
3791956

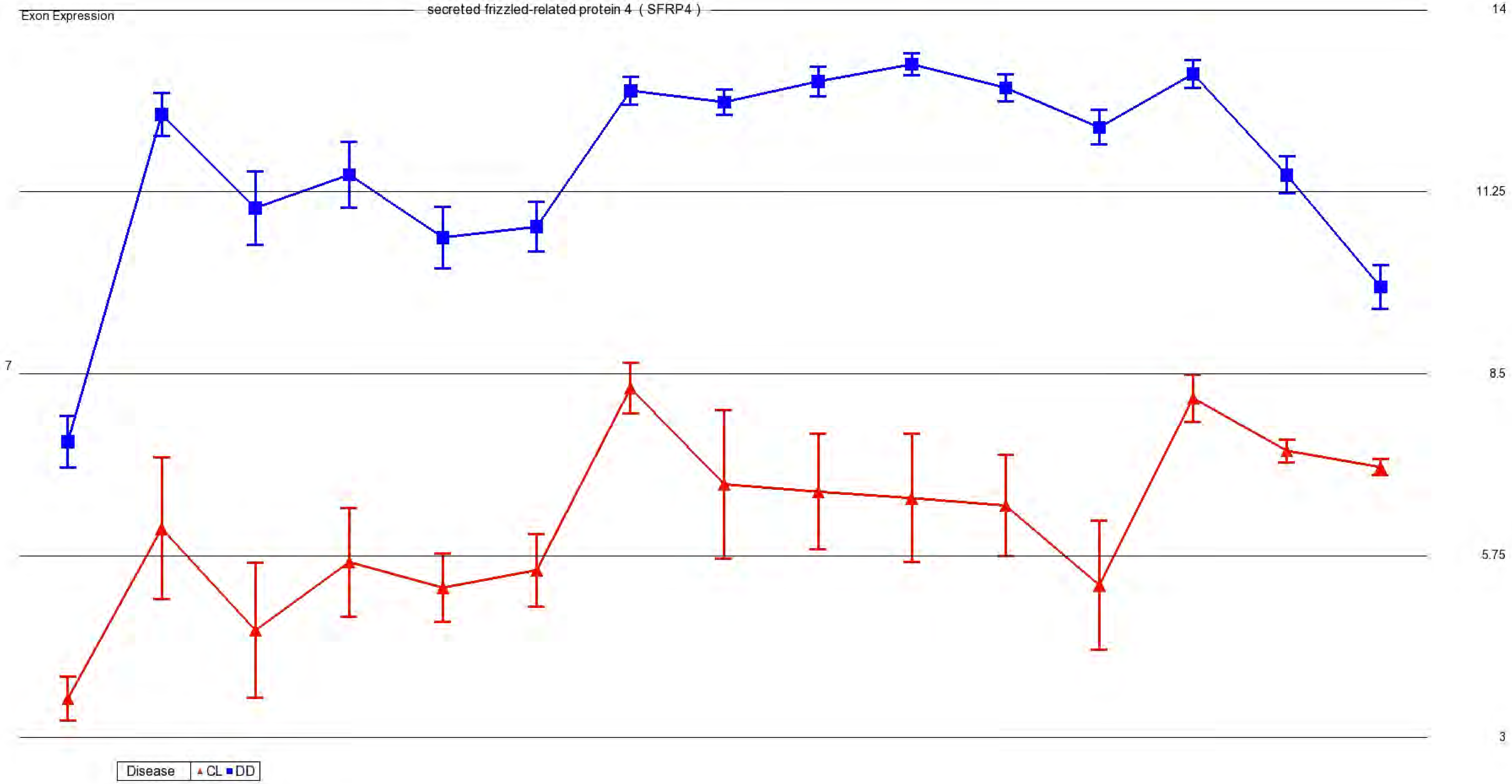

Exon Expression
secreted frizzled-related protein 4 (SFRP4)
14
11.25
7
8.5
5.75
3
Disease
CL
DD

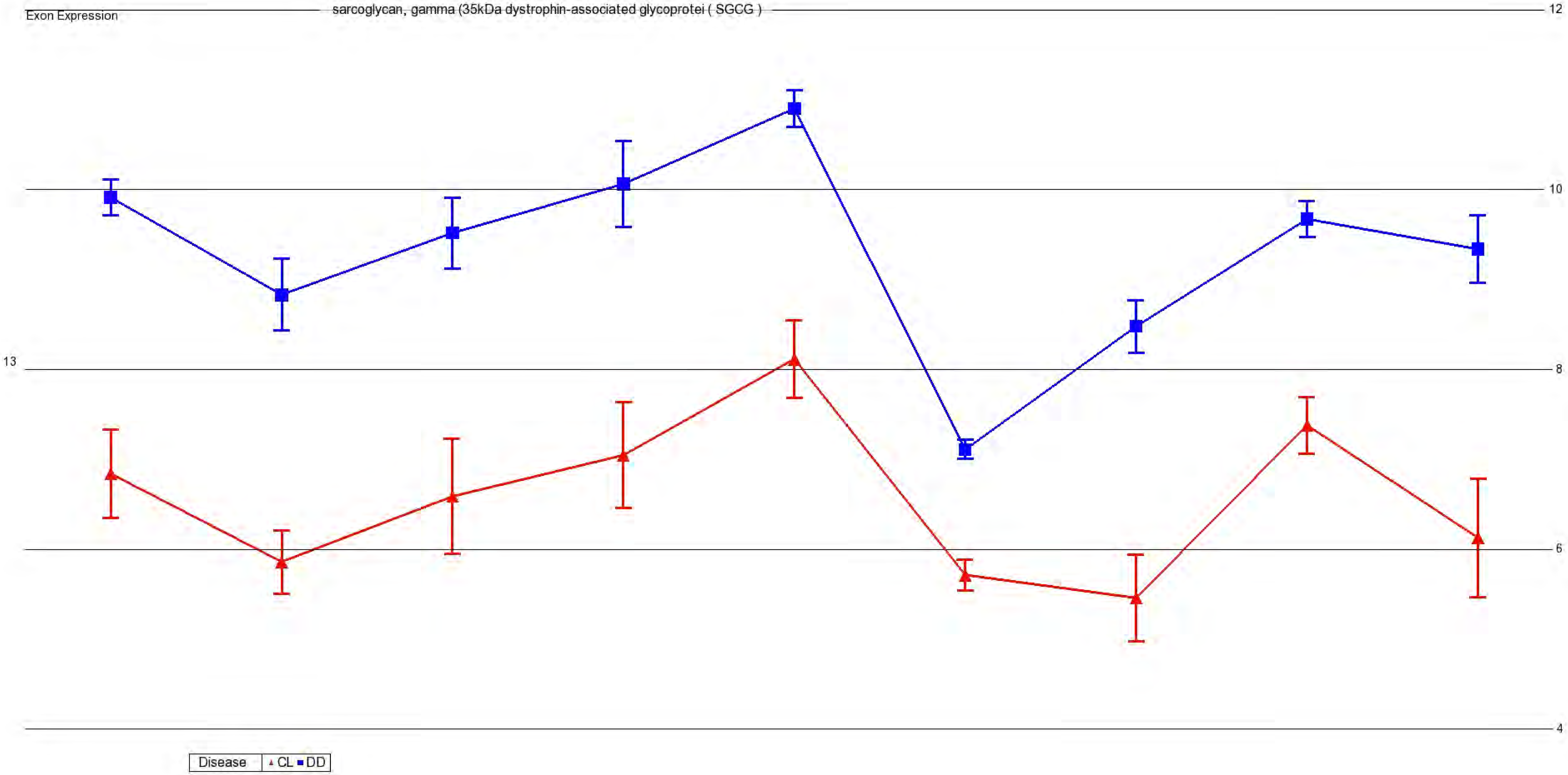

Exon Expression
sarcoglycan, gamma (35kDa dystrophin-associated glycoprotei (SGCG)
12
10
13
8
6
4
Disease
CL
DD

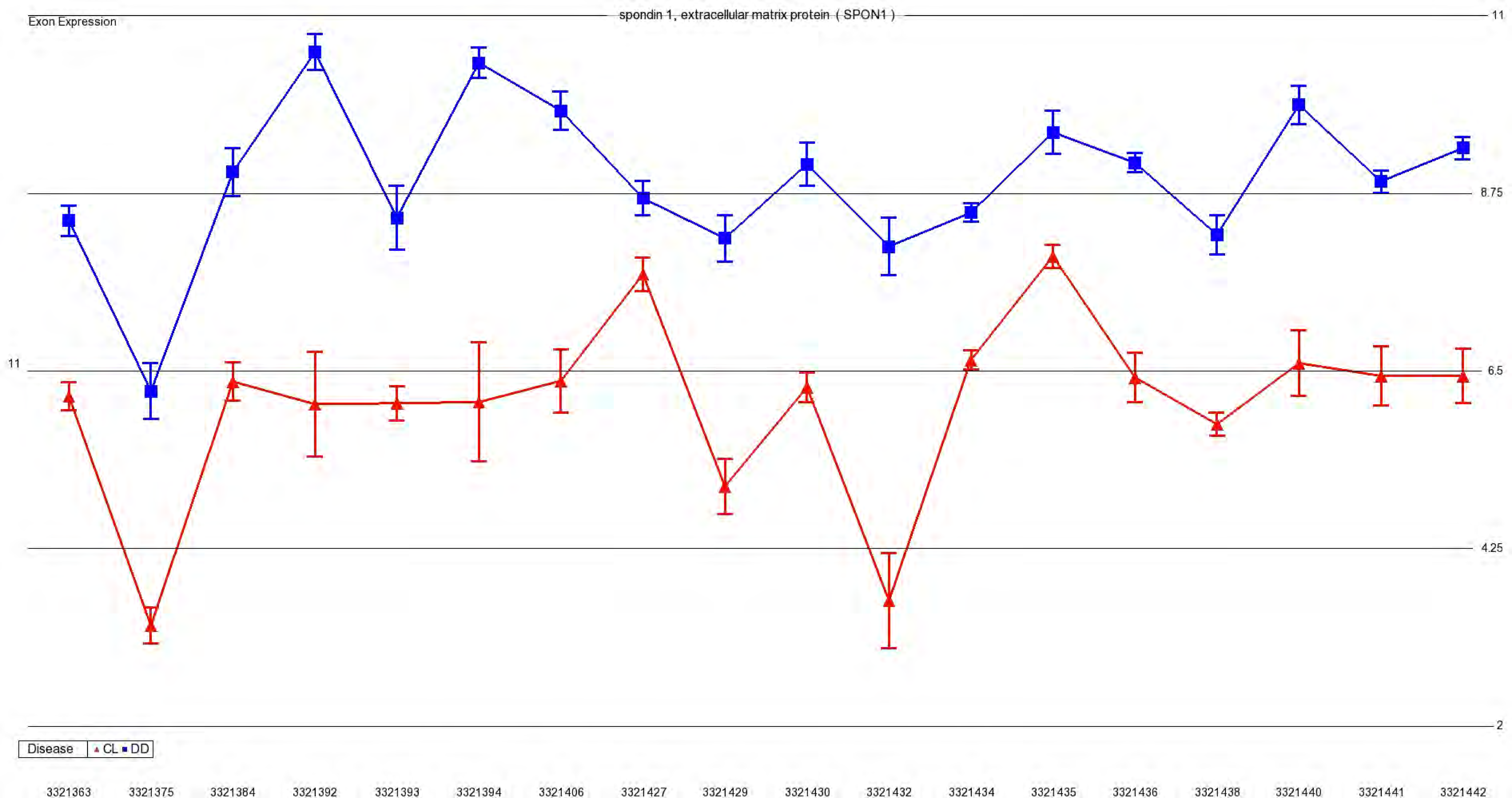

spondin 1, extracellular matrix protein (SPON1)
Exon Expression
11
8.75
6.5
4.25
2
11
Disease
CL
DD
3321363
3321375
3321384
3321392
3321393
3321394
3321406
3321427
3321429
3321430
3321432
3321434
3321435
3321436
3321438
3321440
3321441
3321442

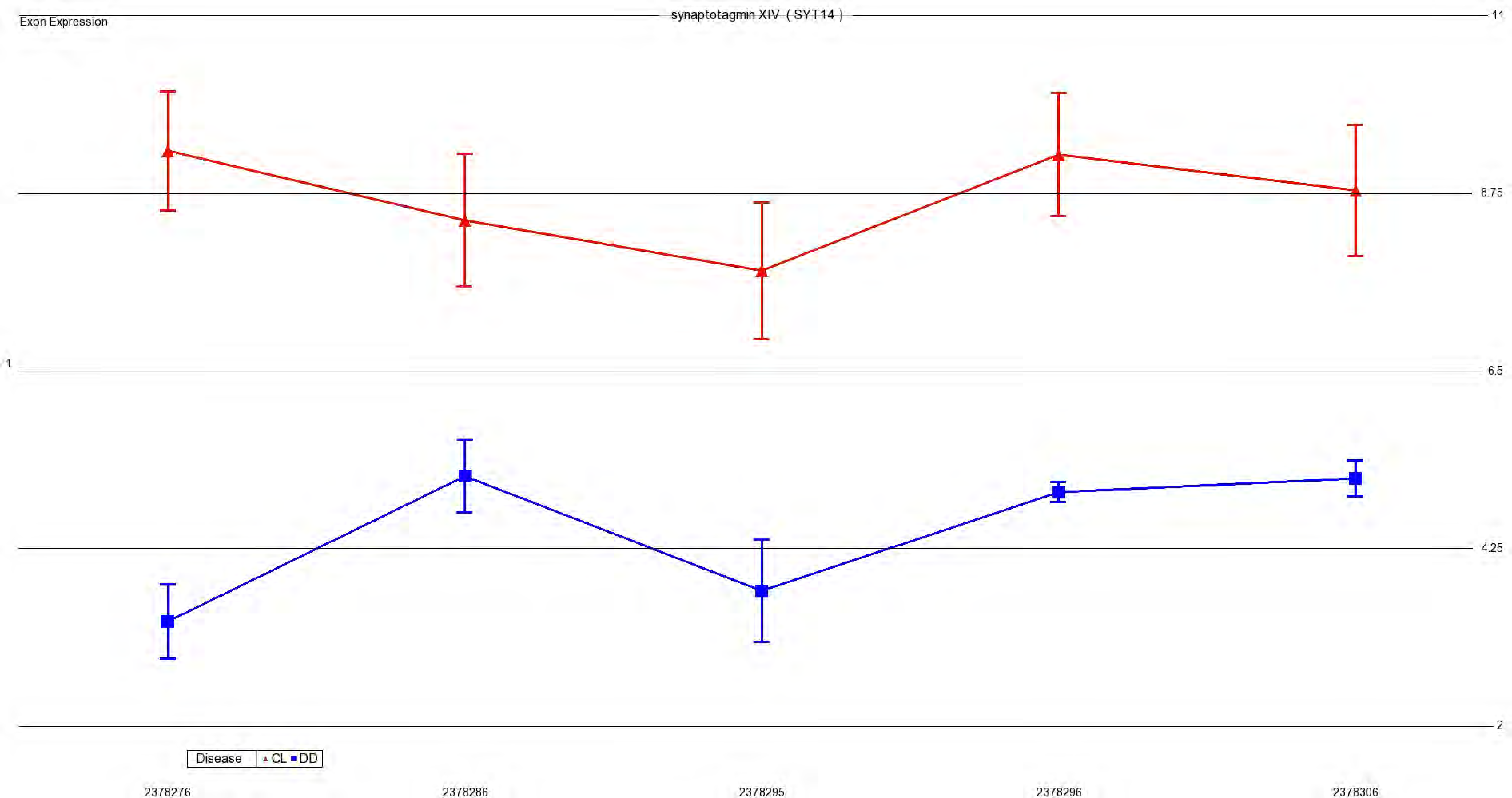
synaptotagmin XIV (SYT14)
Exon Expression
11
8.75
6.5
4.25
2
1
Disease
CL
DD
2378276
2378286
2378295
2378296
2378306

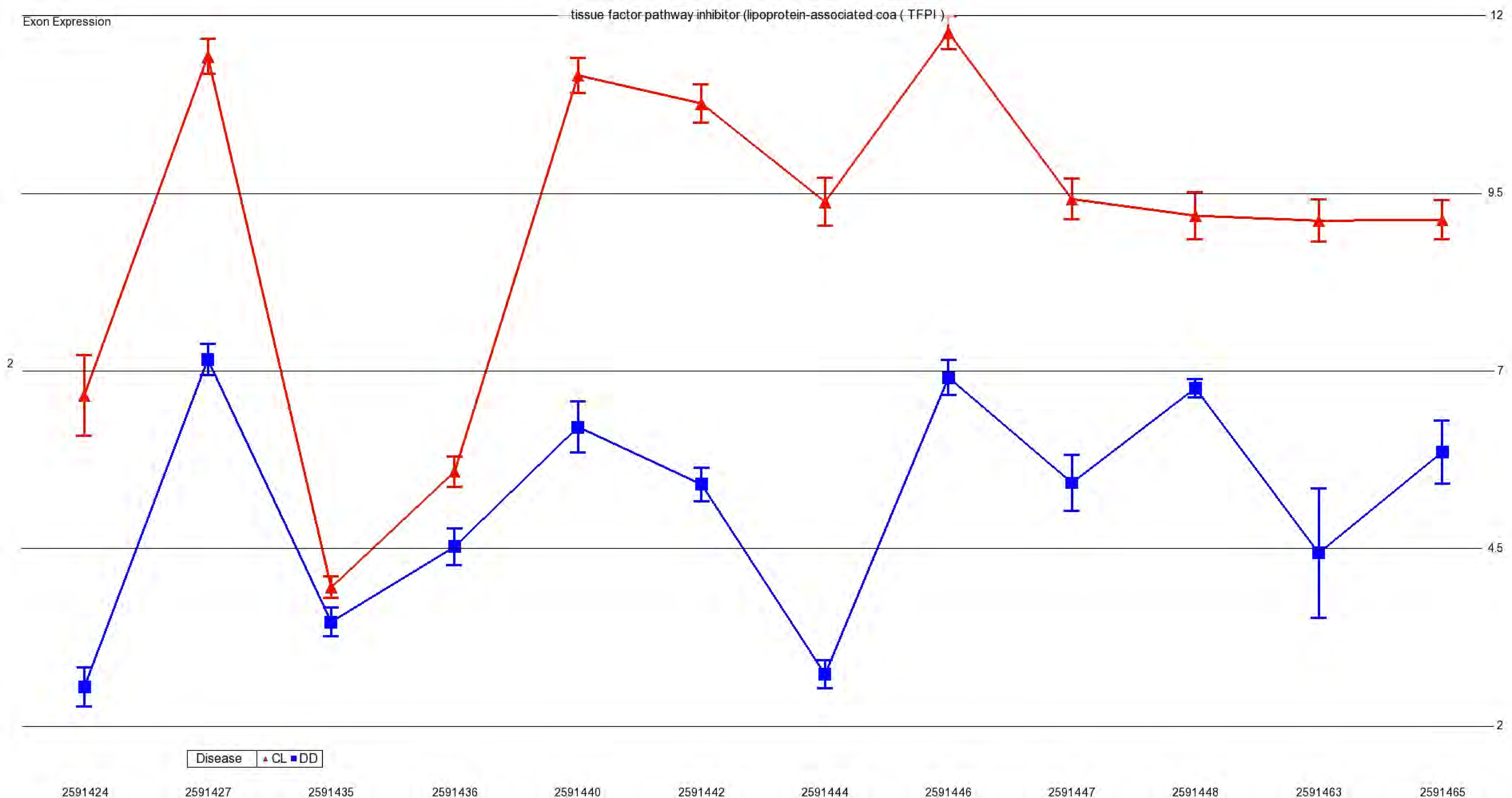

tissue factor pathway inhibitor (lipoprotein-associated coa (TFPI)
Exon Expression
12
9.5
7
4.5
2
2
Disease
CL
DD
2591424
2591427
2591435
2591436
2591440
2591442
2591444
2591446
2591447
2591448
2591463
2591465

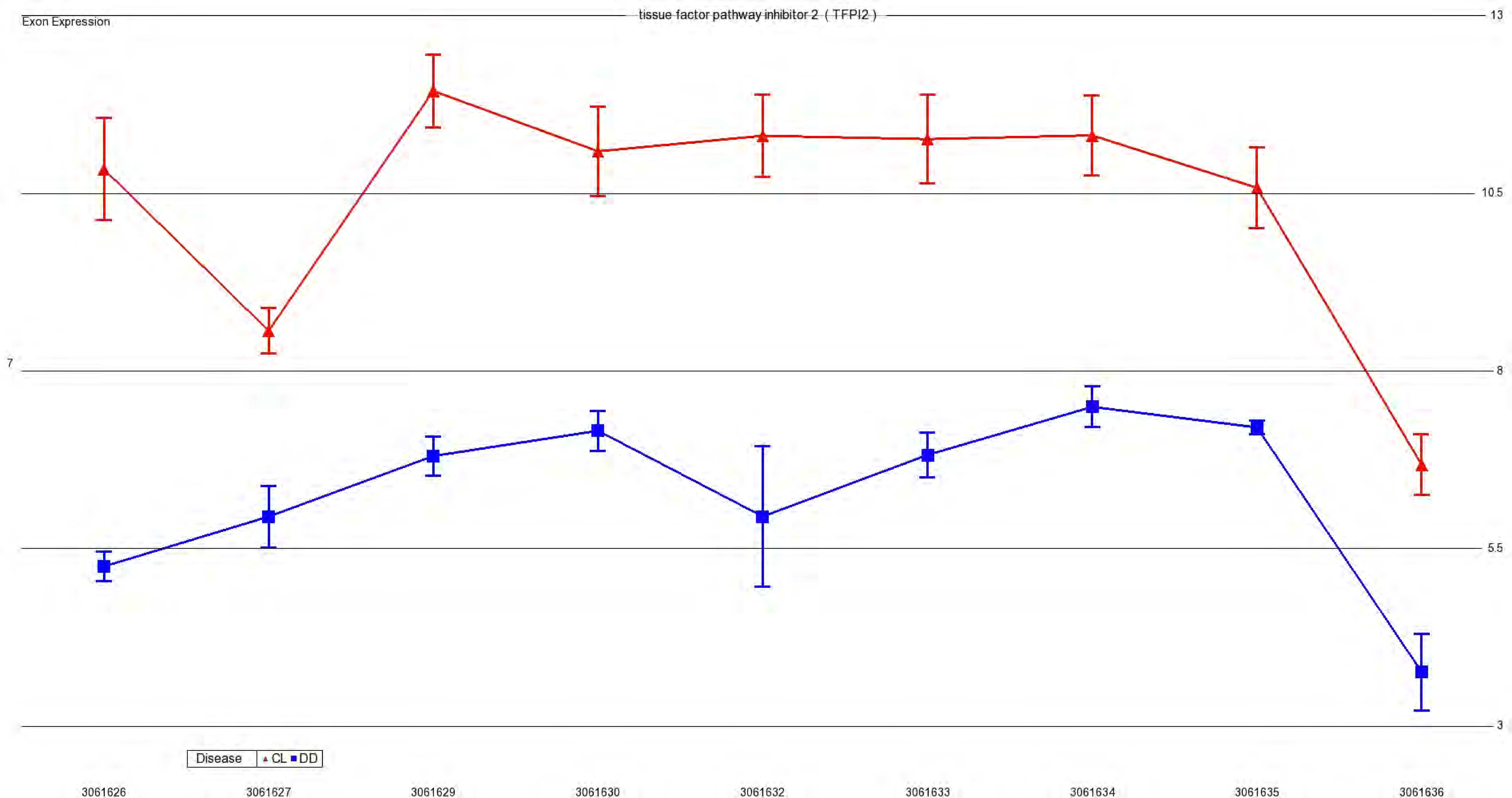
tissue factor pathway inhibitor 2 (TFPI2)
Exon Expression
13
10.5
8
5.5
3
7
Disease
CL
DD
3061626
3061627
3061629
3061630
3061632
3061633
3061634
3061635
3061636

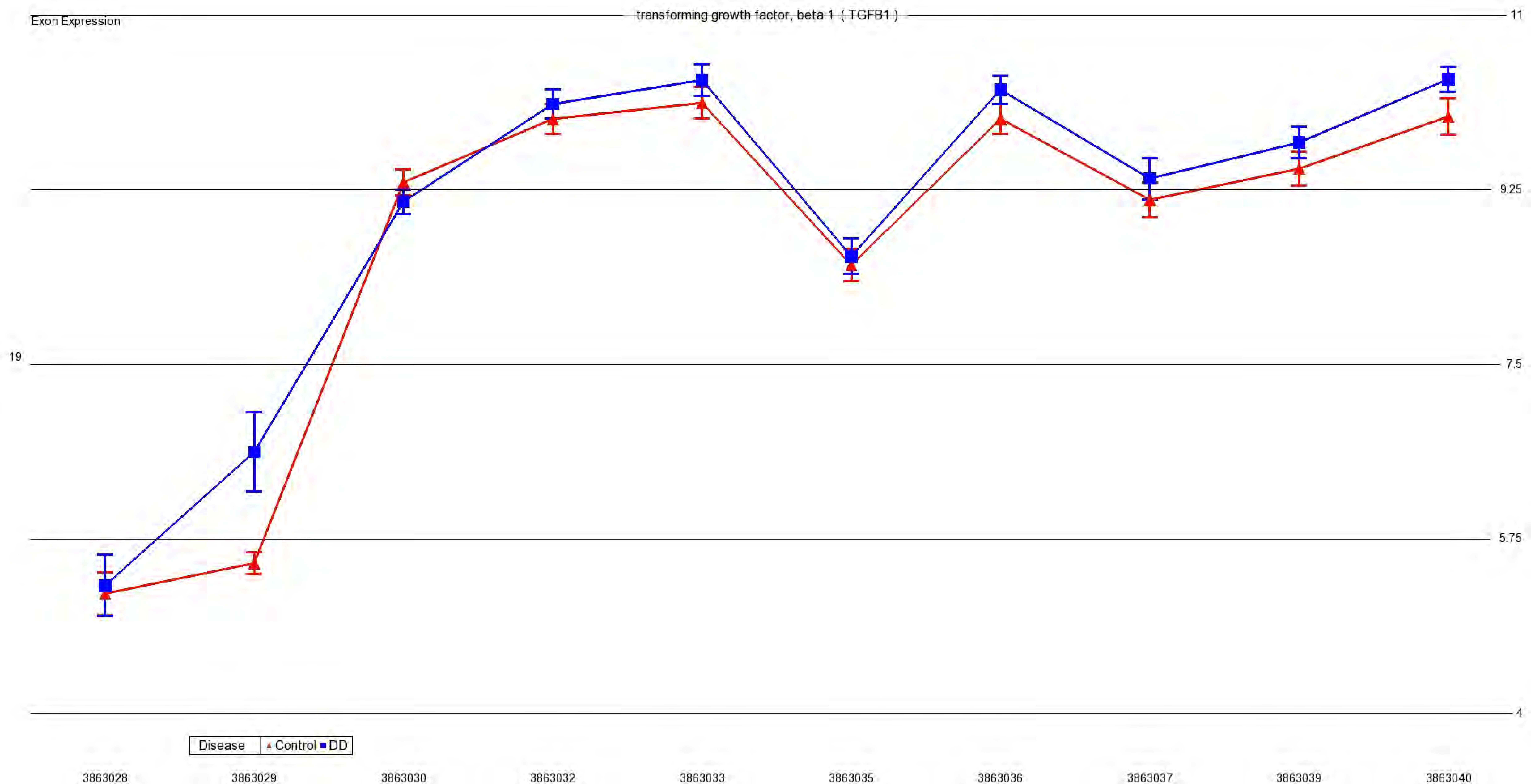

transforming growth factor, beta 1 (TGFB1)
Exon Expression
19
11
9.25
7.5
5.75
4
Disease
Control
DD
3863028
3863029
3863030
3863032
3863033
3863035
3863036
3863037
3863039
3863040

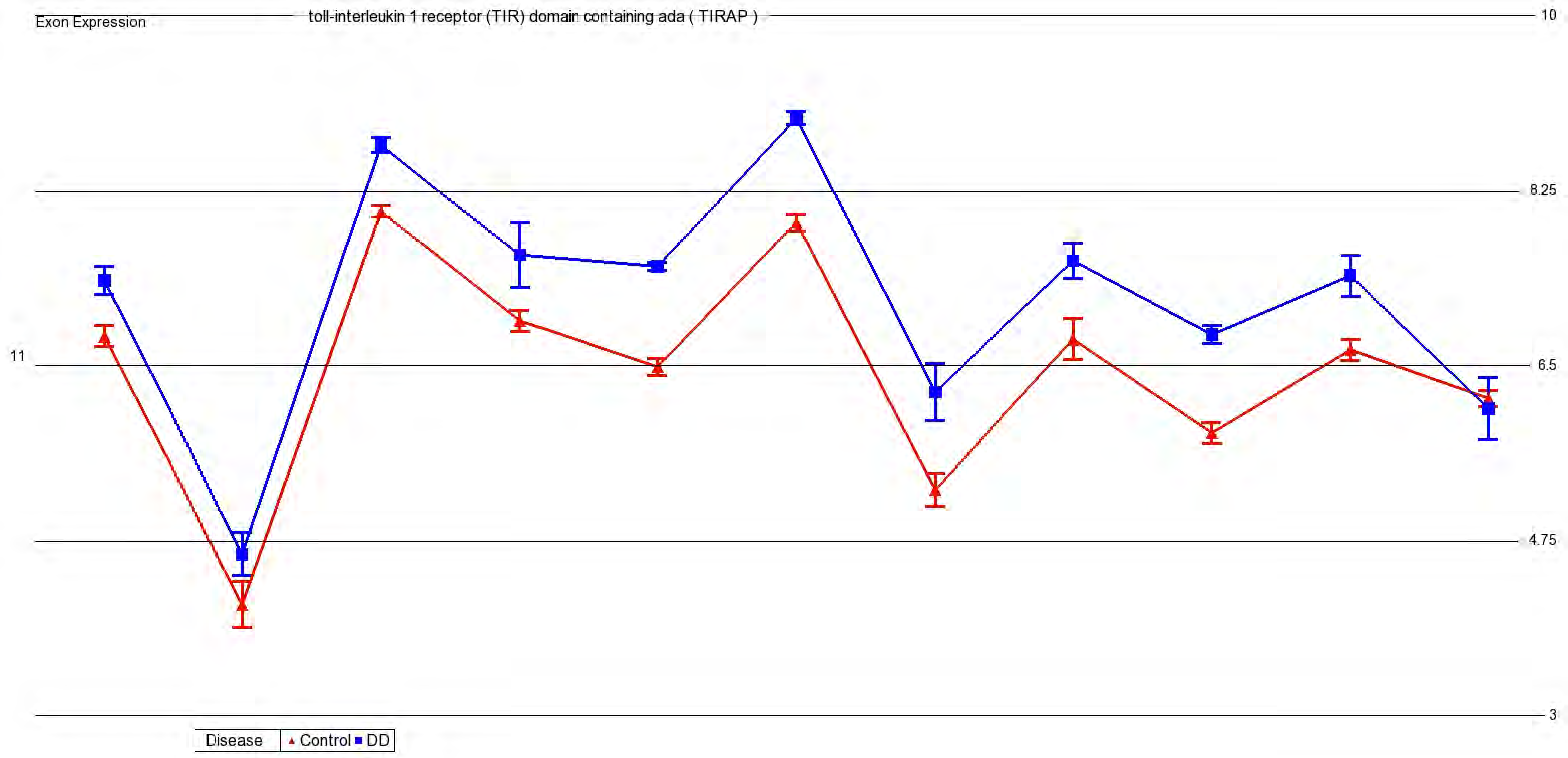
Exon Expression
toll-interleukin 1 receptor (TIR) domain containing ada (TIRAP)
10
8.25
11
6.5
4.75
3
Disease
Control
DD

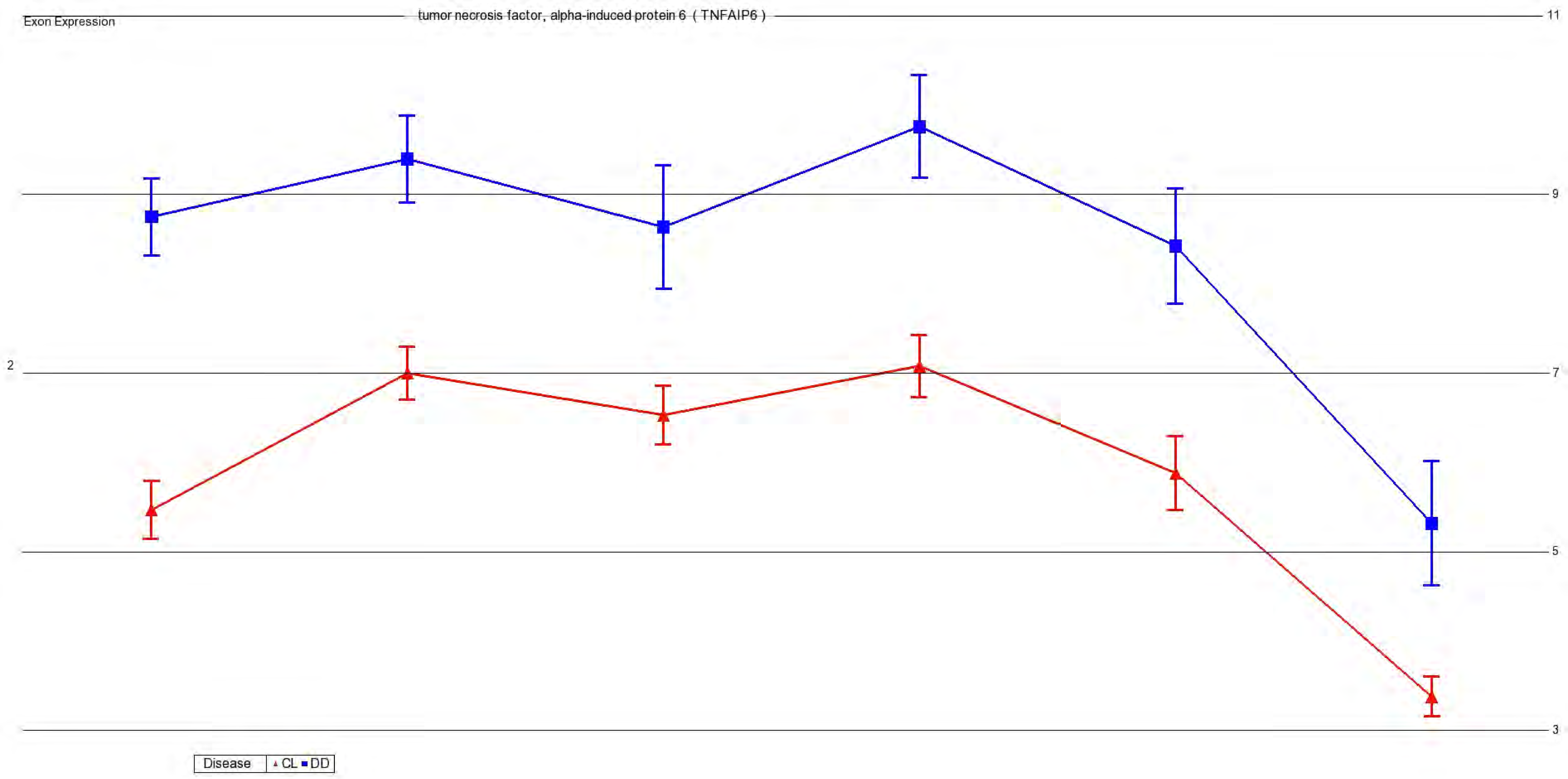
Exon Expression
tumor necrosis factor, alpha-induced protein 6 (TNFAIP6)
11
9
2
7
5
3
Disease
CL
DD

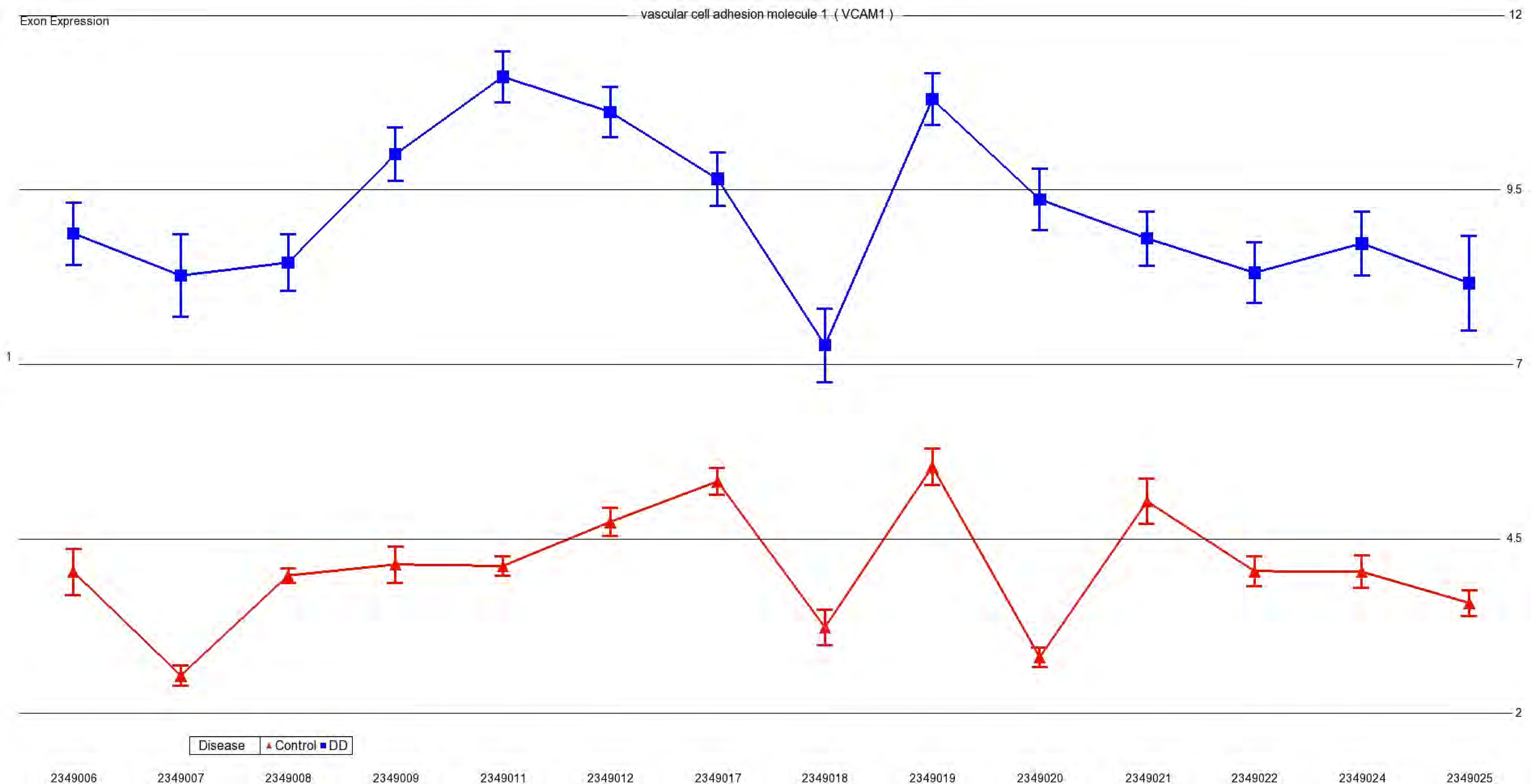
vascular cell adhesion molecule 1 (VCAM1)
Exon Expression
12
9.5
1
7
4.5
2
Disease
Control
DD
2349006
2349007
2349008
2349009
2349011
2349012
2349017
2349018
2349019
2349020
2349021
2349022
2349024
2349025
