# Supplementary figures and images for: Genome-Wide Analysis Using Exon Arrays Demonstrates an Important Role for Expression of Extra-Cellular Matrix, Fibrotic Control and Tissue Remodelling Genes in Dupuytren's Disease
Source: PLoS One. 2013 Mar 12;8(3):e59056. doi: 10.1371/journal.pone.0059056 (PMC3595223; doi:10.1371/journal.pone.0059056)

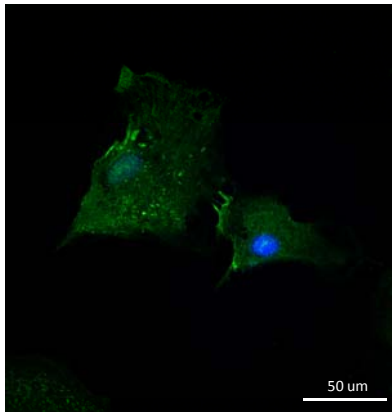

Control 1

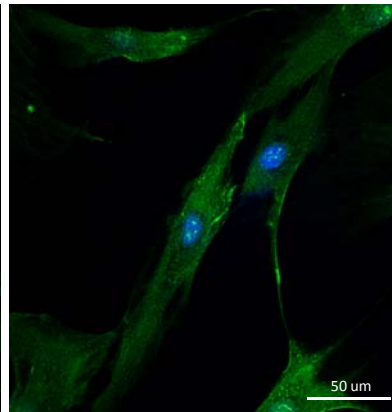

Control 2

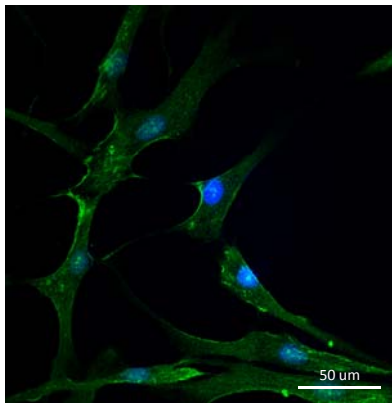

DD1

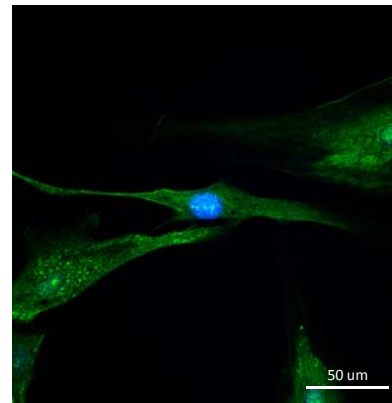

DD2

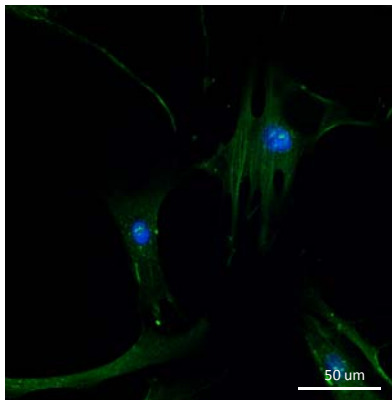

DD3

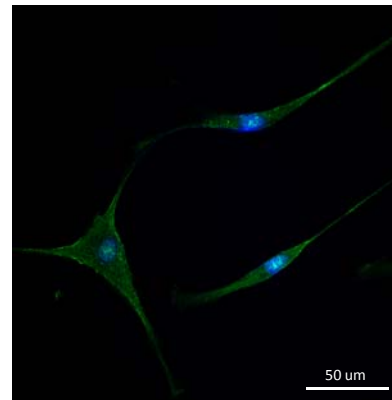

DD4

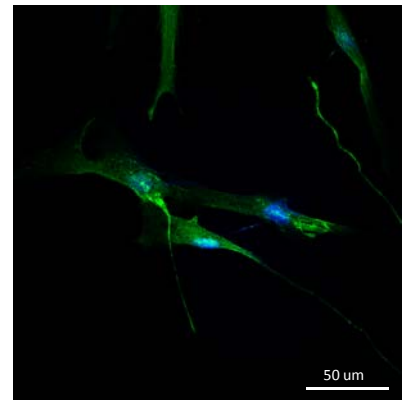

DD5

Figure S2

Supplement: Figure S2 — αSMA staining of primary fibroblast cells. Two control primary fibroblast cell lines, Control 1 and control 2, and 5 primary fibroblasts from DD patients were stained with αSMA (green) and counterstained with chromatin staining Hoechst (blue). Bar in picture is 50 micrometers long. (PDF) [file pone.0059056.s002.pdf]
